# News credibility labels have limited average effects on news diet quality and fail to reduce misperceptions

**DOI:** 10.1126/sciadv.abl3844

**Published:** 2022-05-06

**Authors:** Kevin Aslett, Andrew M. Guess, Richard Bonneau, Jonathan Nagler, Joshua A. Tucker

**Affiliations:** 1 Center for Social Media and Politics, New York University, New York, NY, USA.; 2 Department of Politics, Princeton University, Princeton, NJ, USA.; 3 Department of Biology, New York University, New York, NY, USA.; 4 Wilf Family Department of Politics, New York University, New York, NY, USA.

## Abstract

As the primary arena for viral misinformation shifts toward transnational threats, the search continues for scalable countermeasures compatible with principles of transparency and free expression. We conducted a randomized field experiment evaluating the impact of source credibility labels embedded in users’ social feeds and search results pages. By combining representative surveys (*n* = 3337) and digital trace data (*n* = 968) from a subset of respondents, we provide a rare ecologically valid test of such an intervention on both attitudes and behavior. On average across the sample, we are unable to detect changes in real-world consumption of news from low-quality sources after 3 weeks. We can also rule out small effects on perceived accuracy of popular misinformation spread about the Black Lives Matter movement and coronavirus disease 2019. However, we present suggestive evidence of a substantively meaningful increase in news diet quality among the heaviest consumers of misinformation. We discuss the implications of our findings for scholars and practitioners.

## INTRODUCTION

The internet and social media have drastically decreased the cost of disseminating information by reducing reliance on traditional gatekeepers. As a consequence of this openness and availability, news and information sources have flourished from a variety of ideological and cultural perspectives. The resulting cacophony has encouraged participation by previously underrepresented voices and enabled criticism of dominant authorities. At the same time, it has intersected with existing political divisions in ways that have contributed to pathologies in American political discourse including the spread of misinformation (*1*–*5*), disagreements about basic facts related to governance and policy (*6*, *7*), and lowered trust in established media (*8*). Of particular concern is the possibility that these problems are interlinked: As political divisions widen, partisan media alienate people from authoritative sources, which could make it more difficult to counteract potentially corrosive—and in the case of public health during a pandemic, life-threatening (*9*)—misinformation.

**Fig. 1.**
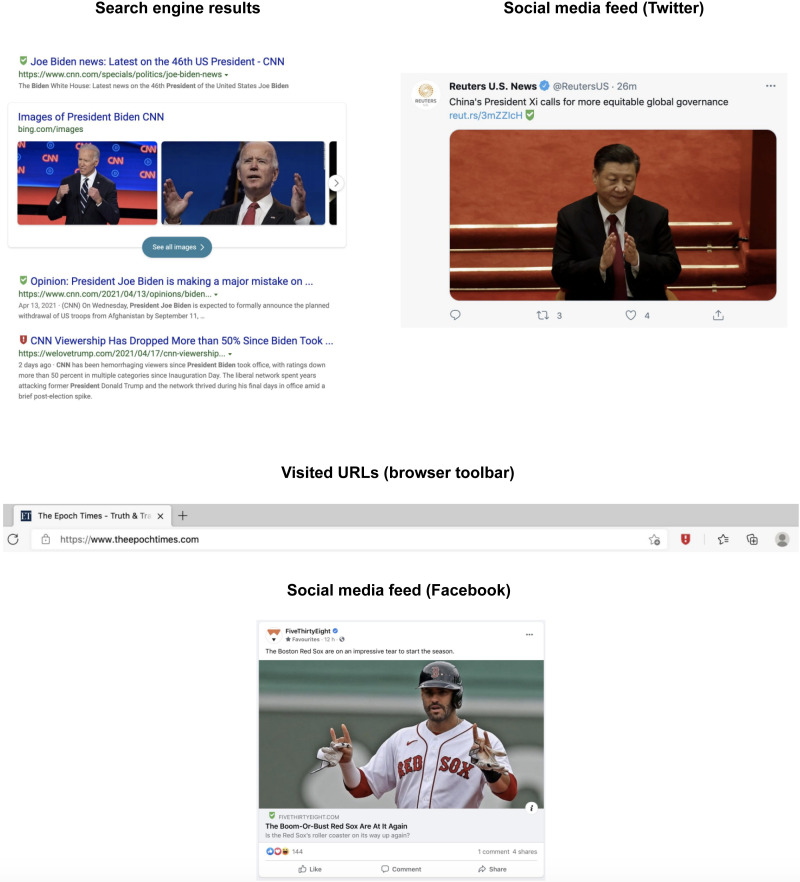
This figure displays how users are exposed to NewsGuard source labels in internet users’ SERPs, social feeds, and visited URLs.

Over the past several years, scholars, technologists, and policy-makers have proposed a number of solutions intended to reduce exposure to misleading information. These range from relatively intrusive measures such as algorithmic downranking to subtle warnings and labels targeted at specific factual claims (*10*, *11*) and to general efforts to boost digital media literacy skills (*12*, *13*). A key challenge in these efforts is how to balance the strength of an intervention with potential negative externalities in the form of unintended spillover effects (*14*, *15*) or limits on individual autonomy and freedom of expression. With this tension in mind, we focus on simple feedback in the form of informational labels designed to educate people about the quality of sources that they consume and view in their search or social media feeds (*16*). This approach builds on humans’ tendency to rely on cognitive shortcuts and heuristics, which, depending on context, can be relatively informative (*17*, *18*) or potentially distorting (*19*). In addition to being scalable relative to fact-checks, source labels are relevant for a broad array of publishers across the spectrum of reliability, rather than merely those designated as purveyors of misinformation. In this study, we build on recent innovations for rigorously evaluating online tools (*20*, *21*). In an online field experiment, we randomly encouraged participants to install a prominent web browser extension, NewsGuard, which embeds straightforward source-level indicators of news reliability into users’ search engine results pages (SERPs), social feeds, and visited URLs. Different “shield” symbols are placed in feed to provide visual summaries of sources’ quality. A green shield indicates a reliable source (examples include CNN, Fox News, and *The Washington Post*), a red shield indicates an unreliable source (examples include Gateway Pundit, Epoch News, and Daily Kos), a gray shield indicates a source with user-generated content (such as YouTube, Wikipedia, and Reddit), and a gold shield represents satire (such as *The Onion*, *Babylon Bee*, and *The Daily Mash*). The user can click on the shield to see an overlay of more detailed information about the reliability of the news domain in question. Figure 1 displays how users are exposed to NewsGuard source labels.

Prior research investigates the ability of expert source ratings to affect the believability of claims encountered online, including on health websites and social media (*22*, *23*). Theoretically, these ratings provide credible information to users about the “functional” dimension of a source’s reputation or its objective performance in a well-defined set of criteria as assessed by experts. Although other dimensions of reputation may not be as responsive to these judgments, experimental evidence suggests that these ratings, when shown alongside a mock news article, can influence the perceived truthfulness of the article’s claim (*22*, *24*). NewsGuard provides a particularly comprehensive set of source ratings produced according to transparent criteria. Deploying these source ratings as visible labels across a user’s search and social feeds provides an opportunity to test the effectiveness of expert ratings as a general solution to online misinformation.

Other attempts to leverage source-level information have been mixed. Although experimental demonstrations of source label effects on selective exposure are well known (*25*), studies have failed to convincingly show that these cues affect susceptibility to misinformation (*26*, *27*). Part of the problem may be that people lack sufficient knowledge about many sources (especially smaller, more unreliable ones that are rarely consumed) to be able to make useful inferences solely on the basis of a publisher’s name or logo. Expert ratings of the kind that NewsGuard provides potentially remedy this shortcoming by supplementing source cues with additional, independently verified information (see a Gallup report for survey data from a sample of NewsGuard users) (*28*).

A large, related literature on source credibility examines the attributes of sources that make them more believable (*29*, *30*). Researchers have sought to apply these insights to the problem of online misinformation, although a recent meta-analysis found that source credibility–based interventions were among the weakest of those studied for correcting misinformation (*31*). The treatments considered differ in two important respects from the source reliability labels that we study. First, they do not typically involve news publishers; “source credibility” conceptualizes messengers broadly to encompass institutions, highly esteemed individuals, or other potential trustworthy sources of information in society. Second, these studies specifically test the extent to which credibility can boost the efficacy of corrections to individual pieces of misinformation rather than display, as NewsGuard does, expert ratings alongside any article (sans correction).

Existing work suggests relationships between exposure to misinformation and various well-known pathologies. Misperceptions are the most commonly studied phenomenon (*6*), which can result from direct exposure to online misinformation (*32*, *33*). In this setting, however, participants are not exposed to specific misinformation in a controlled way. More generally, scholars have suggested reactions to online “fake news,” and the epistemic chaos that it engenders, in the form of heightened cynicism toward politics overall (*1*) and lower trust in traditional media (*34*, *35*). In addition, studies have posited that exposure to misinformation can increase affective polarization (*36*–*38*) because such hyperbolic, misleading content is often engineered to fuel disdain for the partisan outgroup. Given these findings, we examine whether source reliability ratings can counteract these effects. This could occur via two mechanisms. First, by reducing the likelihood that individuals click on misleading or unreliable sources of information, these ratings would lower exposure to misinformation that can promote corrosive attitudes toward politics and society. Second, repeated exposure to labels alongside headlines of varying believability could induce a learning effect. Given prior evidence, it is likely that individuals will either encounter mainly reliable sources or a mix of the two. In the former case, positive ratings could boost news sources’ credibility in general, while the latter case creates an opportunity for improved discernment between reliable and unreliable news sources (*23*). This also allows for a virtuous cycle in which people are gradually exposed to less inflammatory content about the political outgroup.

Our main hypotheses thus test whether in-feed source reliability labels shift downstream news and information consumption from unreliable sources known for publishing misleading or false content to more reliable sources [hypothesis 1 (H1)], increase trust in mainstream media and reliable sources (H2), and mitigate phenomena associated with democratic dysfunction (affective polarization and political cynicism) (H3). We also consider three research questions for which our a priori expectations were less clear. First, past research suggests that certain kinds of interventions can reduce people’s beliefs in both accurate and inaccurate information (*11*, *12*), so we examine whether respondents encouraged to install the NewsGuard extension were more or less likely to believe popular false and true stories that spread during the treatment period. We were not able to preregister this research question because we selected the items as close to fielding as possible. We include the results because they are more directly comparable to studies evaluating the effects of interventions designed specifically to reduce misperceptions. Second, we explore whether downstream effects are observable on other outcomes such as trust in institutions, belief that fake news is a problem in general, and belief that fake news is a problem in the mainstream media. Third, we explore whether any of the identified effects are greater among subgroups found in prior research to more frequently engage with online misinformation. These groups include those who use social media sites more frequently, have low levels of digital literacy, consume more news, and already visit more online publishers of untrustworthy news. Results from all of our preregistered analyses can be found in the Supplementary Materials, sections SC to SH.

Combining panel survey data and individual-level web visit data, we find in preregistered analyses that in-browser contextual source labels (i) do not measurably shift participants’ online consumption from unreliable sources known for publishing misleading or false content to more reliable sources, (ii) fail to reduce average belief in widely circulated inaccurate claims, and (iii) do not alter trust in the media generally. Our estimates—especially for survey-based outcomes—are well powered, and we can rule out even very small intent-to-treat (ITT) effects (Cohen’s *d <* 0.07). Our null findings on changes in participants’ information diet quality are somewhat noisier, although we can still rule out small effects (Cohen’s *d <* 0.09) according to standard benchmarks. Our noisier estimates on behavior are themselves informative, because they reflect the well-established reality that people with diets consisting overwhelmingly of untrustworthy news sources are a relatively small subset of the population (*39*). Interventions designed to improve news quality through dynamic feedback may therefore need to focus their efforts on these individuals and tailor the information they provide accordingly. Consistent with this—and in light of our original null findings—we undertake supplementary analyses (not preregistered) and find that the treatment does appear to improve the average reliability score of the news consumed by participants at the lowest decile of pretreatment online news diet quality.

## RESULTS

**Fig. 2.**
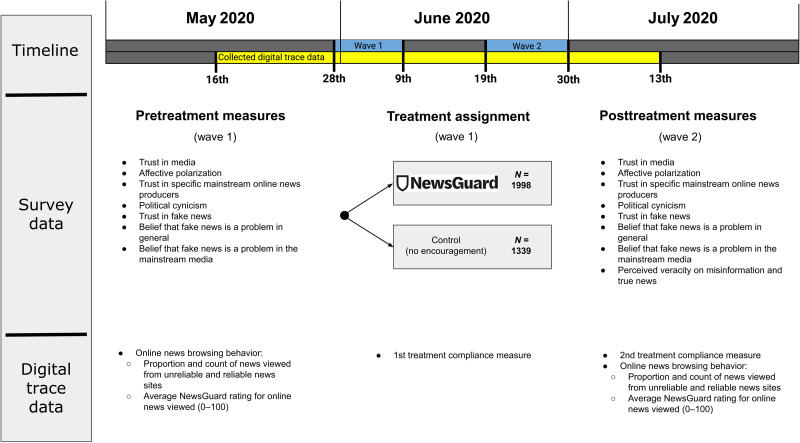
This figure displays a timeline of our data collection surrounding our two-wave online panel survey from 28 May to 30 June 2020 (wave 1: May 28 to June 9, *n* = 3862; wave 2: June 19 to 30, *n* = 3337).

To measure the effect of these source labels, we fielded a two-wave online panel survey from 28 May to 30 June 2020 (wave 1: May 28 to June 9, *n* = 3862; wave 2: June 19 to 30, *n* = 3337) that included a randomized incentive to install the NewsGuard web extension at the beginning of the first wave. Enough respondents were recruited that we are confident that we could detect even a small standardized effect size (9% of an SD) among respondents for whom we have behavioral data and the larger survey sample. Results from our power analyses can be found in section SJ. Figure 2 presents an overview of the study design. In addition to studying survey-based outcomes, we analyze linked digital trace data to measure the quality of news consumption of a subset of our participants. We create five distinct measures of news diet quality (details can be found in Materials and Methods) over three time periods: (i) the period before a respondent was assigned treatment in the wave 1 survey, (ii) the 3- to 4-week period from treatment assignment (May 28 to June 9) to June 30, and (iii) the nearly 2-week period from July 1 to 13. Testing the effect of this treatment on news consumption during the third period was not a part of our original set of preregistered hypotheses, but rather a preregistered research question. Using the measurement of news consumption in the third period, we leverage the exogenous disabling of NewsGuard’s free capabilities on July 1 to determine whether the behavioral effects of this intervention decay after its features are no longer available (*40*) or whether the intervention has more durable effects similar to other informational nudges (*41*).

In this section, we primarily report the covariate-adjusted estimates of an ITT effect measuring differences between the control and treatment groups on outcomes of interest. In the Supplementary Materials (section SC), we report the covariate-adjusted estimates of complier average causal effects (CACE), the effect among those who installed the browser extension as a result of the randomized treatment. To verify treatment compliance, we developed an automated script linked in the survey that measured whether participants in the treatment and control groups had installed and activated the NewsGuard extension on their web browsers twice: directly after the treatment was assigned in wave 1 and in the last week of the treatment period. Ninety-five percent of respondents in the treatment group passed the first compliance check, and 80% passed both the first and second compliance check (about 1% of those in the control group already had the NewsGuard extension installed and activated). For most demographic characteristics, we find no statistically significant evidence that respondents who comply are very much different than those who did not comply; moreover, for demographic characteristics where we find a statistically significant difference, the magnitudes of these differences are very small. More details on how compliance was estimated, compliance rates, and how compliers differed from noncompliers can be found in Materials and Methods.

Because a lack of statistically significant coefficients does not necessarily imply effects of a negligible magnitude, we use equivalence testing to rule out any meaningful effects in all of the following models (*42*–*44*). Conservatively, negligible effects are defined as those smaller than 20% of an SD (*45*) of the population on a pretreatment measure of a variable, although others have advocated for higher thresholds (*44*, *46*). We thus calculate standardized effects for each estimate shown. The magnitudes of these standardized effects never rise above 10% of an SD, thus remaining far below the most conservative established threshold, rejecting the hypothesis of even a small effect on the outcome variable. In addition to these standardized effect sizes, we also estimate the minimum detectable effects for covariate-adjusted ITT models reported in the main text in section SJ. Assuming power of 0.80 and that statistically significant effects must cross the *P* value threshold of 0.05, the models can, at a minimum, detect effects larger than 6.5% of an SD for all of the attitudinal measures and any effect larger than 8.9% for all of the behavioral measures. Our design is thus well powered to detect small effects of the intervention (although this is generally not the case for interactions).

**Fig. 3.**
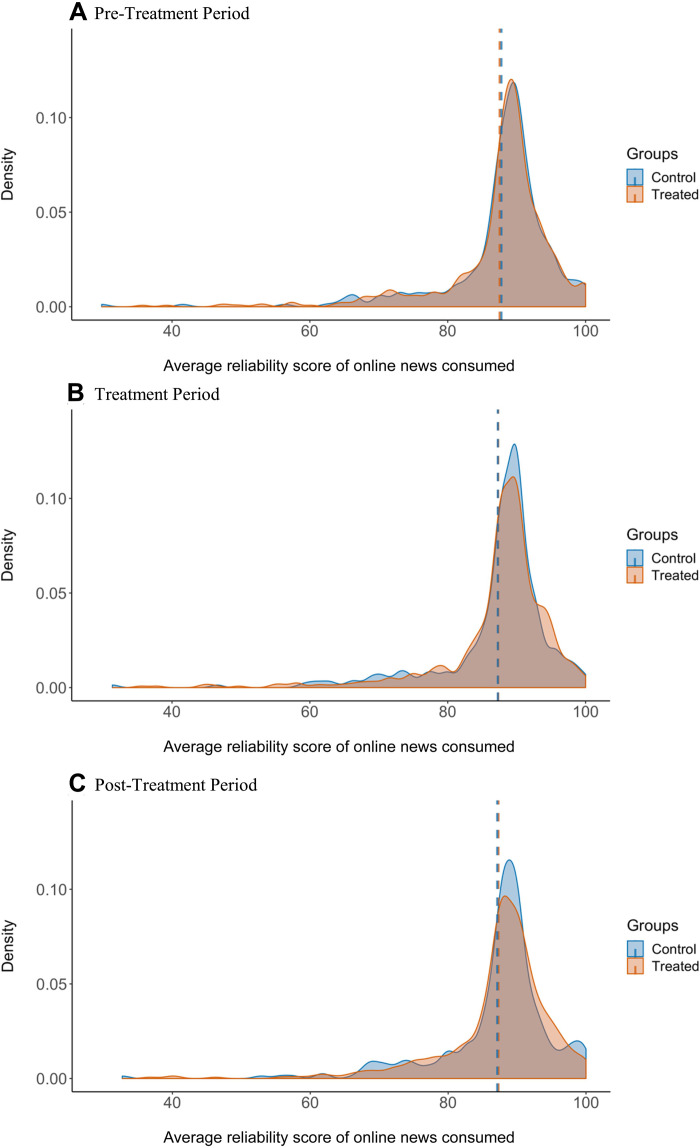
This figure presents the distribution of the average reliability scores of online news consumption among respondents in the treatment and control groups, with vertical dashed lines indicating the mean reliability score in each group. (**A**) Distributions during the pretreatment period. (**B**) Distributions during the treatment period. (**C**) Distributions during the posttreatment period.

In our sample of respondents for whom we collect behavioral news consumption data (*n* = 968), most did not visit an unreliable news site during the 2- to 3-week pretreatment period (more than 65%) and only less than 12% of our sample’s news diet consisted of at least 5% of visits to news sites deemed unreliable by NewsGuard. Only 1.5% of respondents’ news diets had an average NewsGuard reliability score below 60 (the threshold for reliability). This distribution appears consistent across the treatment and control groups. Figure 3 (A to C) presents the distribution of average online reliability scores of respondents in both the control and treatment groups over the three time periods of interest. In section SM, we present the distributions of the other four behavioral measures in each time period. The long tail of low-quality online news consumption is in line with what work has reported (*39*), although the relatively rare prevalence of visits to unreliable sites among our respondents creates a challenge in terms of statistical power when estimating treatment effects on the behavioral measures focused on the consumption of unreliable news (the proportion of unreliable news and the count of unreliable news) among the full population. For this reason, we also test the effect of the treatment on the subset of respondents who consume the most low-quality news. Given that the average NewsGuard reliability score of respondents’ news diets was 87.6 of 100 and 67.4% of news viewed was considered reliable (scores of 60 or above), there is plausibly sufficient variation for detecting heterogeneous effects on overall news diet quality by quantiles of these pretreatment scores (NewsGuard includes news that does not include a rating, such as sites with user-generated news or satire, but neither are considered reliable or unreliable).

**Fig. 4.**
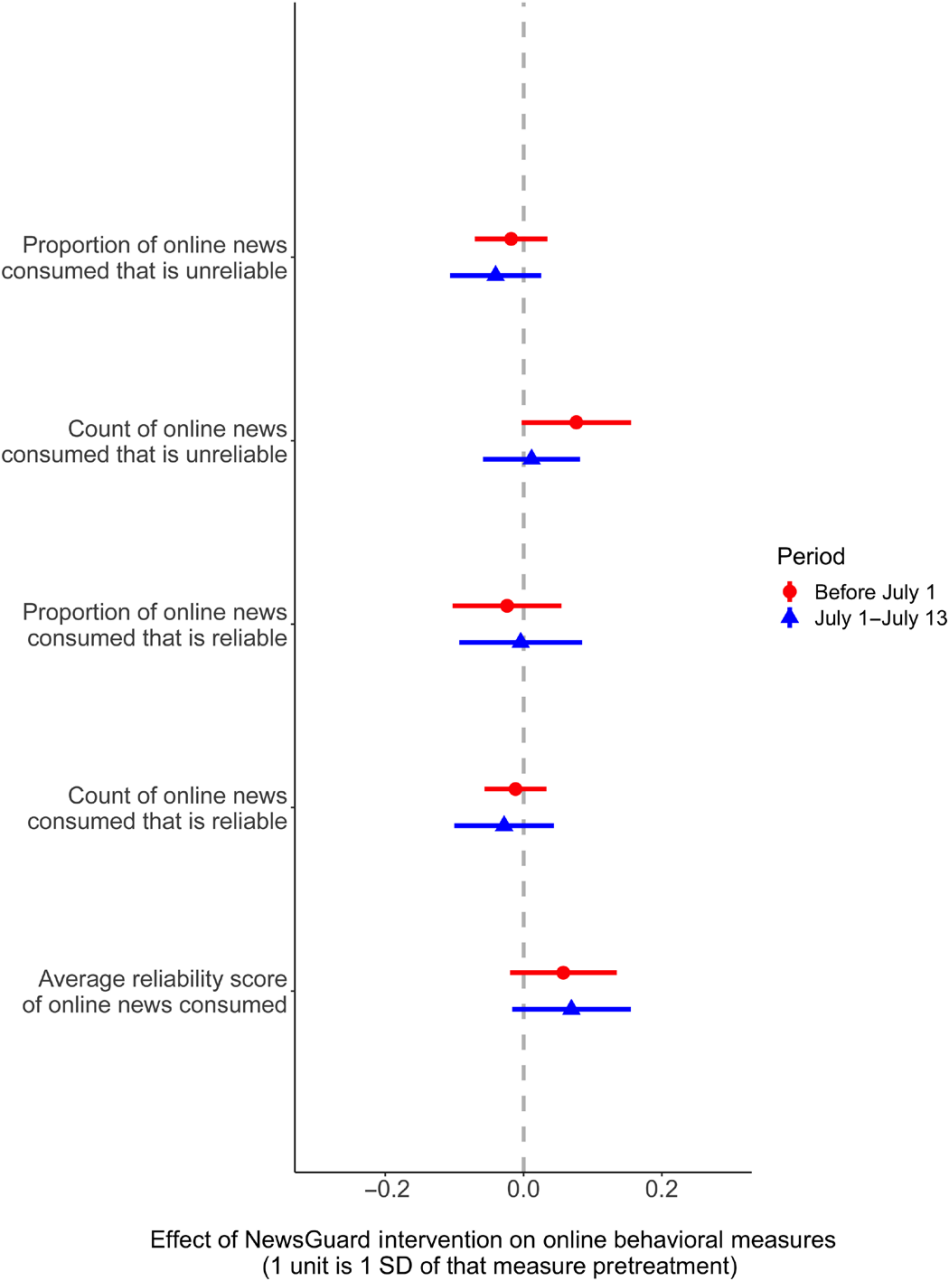
This figure presents estimates of the effect of the intervention (ITT, with 95% confidence intervals) on our preregistered online behavioral measures in the two periods after treatment assignment: before July 1 when the NewsGuard extension was freely available and the 2-week period between July 1 and 13 when the NewsGuard extension was disabled. The effect is reported in SDs of that measure (pretreatment).

Contrary to H1, we do not find that randomized exposure to in-browser source reliability information shifts online consumption of news away from unreliable publishers. We present the treatment effect estimates for each preregistered behavioral outcome in Fig. 4. As the figure indicates, we do not find statistically significant decreases in the proportion of news consumed from unreliable sources or in the count of unreliable online news consumed, either during the period when NewsGuard was installed by those in the treatment group or in the 2 weeks after NewsGuard became a pay service. We also do not find that the intervention measurably shifts individuals toward reliable news: We do not observe statistically significant increases in the proportion of news consumed from reliable sources, in the count of visits to these sources, or in the average reliability score of online news consumed in either time period because of the treatment. In addition to an absence of statistically significant effects, the estimated magnitudes are extremely small: All of the reported effect sizes constitute less than a 0.08 change in the SD of the pretreatment measure of that variable.

**Fig. 5.**
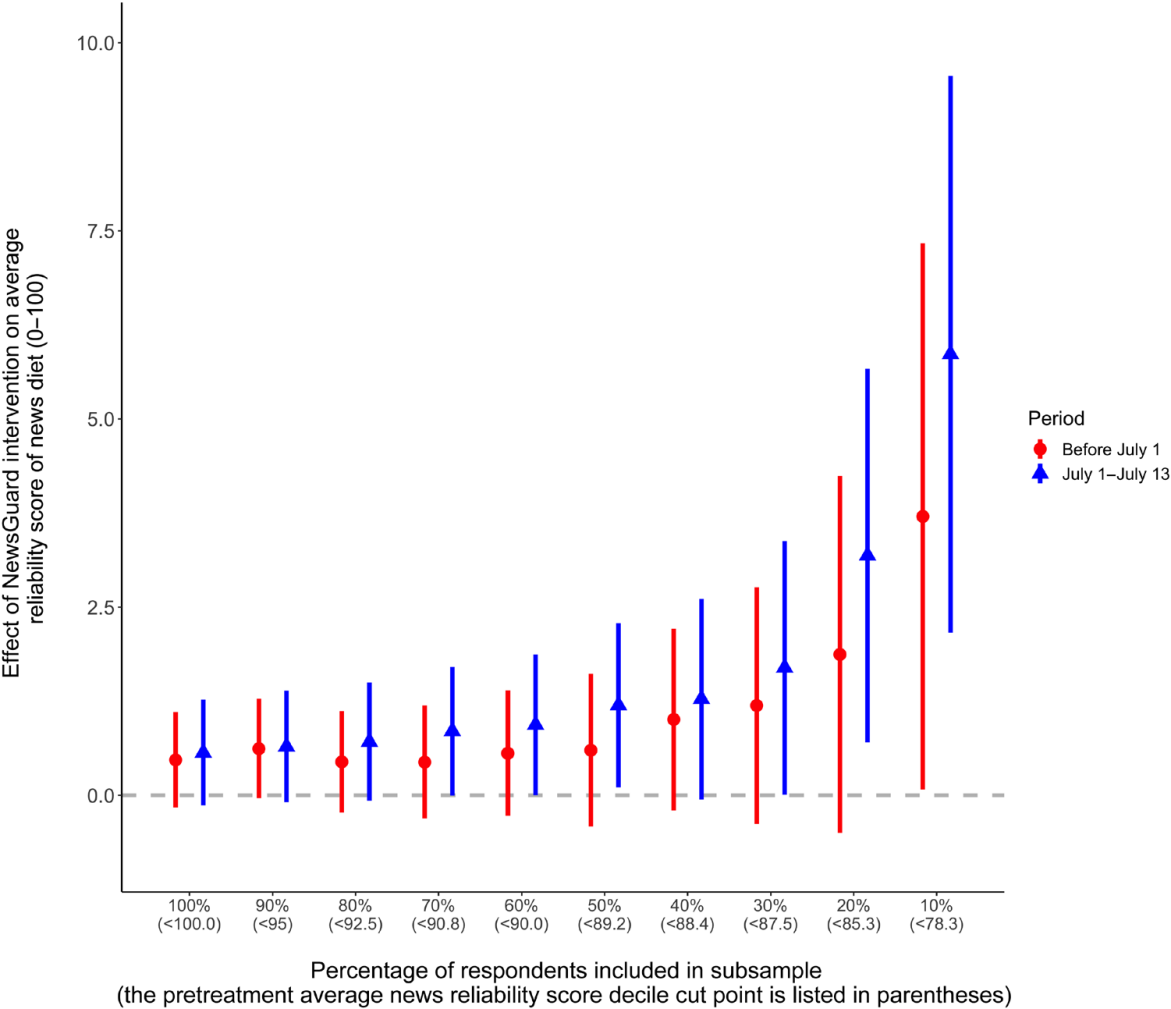
This figure presents estimates of the effect of the intervention on the average NewsGuard reliability score of respondents’ news diets (with 95% confidence intervals). This is estimated for subsets of the sample with pretreatment reliability scores below successive decile cut points.

Given these results, we conducted a series of non-preregistered supplementary analyses to better understand the seemingly negligible impact of the news quality labels on news consumption. To determine whether the treatment affects the time spent on unreliable or reliable news websites, we weight each variable by duration, but this does not measurably change the results (details can be found in section SC). We also measured these variables strictly as referrals from social media sites and search engines and find no statistically significant treatment effects (details can be found in section SC). Last, we divide the sample according to pretreatment deciles of respondents’ average news reliability scores (details for why we chose this specification can be found in Materials and Methods). We find relatively strong and statistically significant treatment effects among those in the lowest decile of news diet reliability (Fig. 5): Relative to the average pretreatment value, we estimate a 5.4% increase in the treatment period and a 8.6% increase beginning July 1 in the average reliability score of news consumed (full results for each behavioral measure across each decile can be found in section SK). The effect appears to be weaker when measured between treatment assignment and July 1 than in the 2 weeks after the extension’s functionality ended, which is consistent with an overtime learning effect as respondents received more feedback about the reliability of online sources in their search results and social feeds. Although suggestive (and likely underpowered), these subgroup effects are consistent with the intervention being mainly effective among those who consume the greatest amount of content from relatively unreliable sources, which can be seen in the tails of the bottom two panels in Fig. 3. We also tested whether treatment effects were detectable among respondents that are more likely to view unreliable online news (older respondents, those with lower levels of digital literacy, and those that self-identify as Republican), but we do not report any statistically significant effects on the quality of online news consumption among these groups.

The next hypothesis predicted that source reliability feedback would increase trust in the media and reliable sources (H2). We find that the treatment does not increase trust in media or in specific reliable news sources. Our estimates of the treatment effect on trust in media and in selected specific sources deemed reliable by NewsGuard are not statistically distinguishable from zero, and the reported effect sizes are less than a 0.05 change in the SD of the pretreatment measure of that variable. A figure depicting the effect of the intervention on our preregistered attitudinal measures can be found in section SK.

We also find no support for our final hypothesis, which predicted that exposure to the treatment could help to alleviate pathologies such as affective polarization and political cynicism associated with consuming, believing, and sharing news from unreliable sources (H3), which, in retrospect, is perhaps expected because we did not find an effect for the treatment on consuming or believing news from unreliable sources. Research Question 1 (RQ1) asked whether source reliability information affects belief in misinformation and accurate claims. To answer this question, all respondents were asked to judge the veracity of five widely circulated statements about the Black Lives Matter (BLM) movement and five similarly well-circulated statements about coronavirus disease 2019 (COVID-19) using a four-point scale in wave 2. Of the five statements about each topic, three were false and two were true. By taking the mean of the perceived veracity measure for the three false statements about each topic, we created a measure of belief in misinformation in the BLM movement and COVID-19, and we likewise created a measure of belief in true information using the other two items. The intervention had no effect on belief in misinformation about the BLM movement and COVID-19, and it did not measurably affect belief in the true statements (reported effect sizes are all less than a 0.03 change in the SD).

Our second research question asked whether exposure to the intervention leads to effects on other outcomes such as trust in institutions, belief that fake news is a problem in general, and belief that fake news is a problem in the mainstream media. We do not find that the intervention measurably affected these outcomes (reported effect sizes are all less than a 0.03 change in the SD). Last, RQ3 asked whether any effects are moderated by specific characteristics (proportion of news consumed that is unreliable, partisanship of news diet, online news consumption, social media use, digital literacy, and belief in scientific misinformation), but we find no other consistent evidence of effect heterogeneity. Results from models measuring the effect of the moderators we preregistered hypotheses about (all moderators we tested are listed in section SA.2) can be found in sections SE and SF.

## DISCUSSION

Despite previous research that reported generally positive assessments (*28*) of browser-based tools designed to reduce users’ reliance on misinformation, evidence from a preregistered randomized field experiment among a large representative sample of Americans reveals that the particular intervention studied here—providing dynamic, in-feed source reliability labels—does not measurably improve news diet quality or reduce misperceptions, on average, among the general population. Our estimates, based on both survey and behavioral data collected over an extended period, are precise and rule out even modest effect sizes by conventional standards.

Although we are able to measure pre- and posttreatment news consumption “in the wild” as well as treatment compliance at the point of installation, we do not capture the contents of participants’ browser experience—what they see but do not click on. This means that we did not directly observe how often users encountered NewsGuard’s labels in the course of browsing their feeds and perusing search results pages during the study period. We can estimate how often they were exposed to these labels by counting the number of visits to online news sites rated by NewsGuard (which trigger a source quality label in the browser bar when the extension is installed), Google search results pages, and time spent on Facebook or Twitter during the pretreatment period; we present distributions of these measures in section SJ. We are most interested in respondents’ time spent on SERPs and social media feeds because users scrolling through these pages are likelier to be exposed to the credibility ratings for multiple news sites as opposed to one at a time. This should theoretically translate to greater potential for the labels’ effectiveness than discrete visits to online news sites rated by NewsGuard, although we find that most of our respondents rarely visit Google SERPs for long periods of time. Specifically, the median time spent on Google SERPs per day in the pretreatment period is only a few seconds, and the mean is 1.6 min. This is consistent with evidence that users typically gravitate toward top-ranked search results (*47*). The relative rarity of exposure to credibility ratings in these contexts may partly explain the negligible effect of this intervention on the quality of online news consumption and downstream attitudes.

Our results speak to a large body of work on heuristics, cognitive processing, and reputation theory (*22*, *30*, *48*). The NewsGuard shield icons provide expertise cues, which have been shown since the work of Hovland *et al*. to be associated with increased source credibility (*29*). However, in a more partisan age in which attitudes toward news sources are strongly correlated with partisanship (*49*), relatively subtle contextual information may not be a sufficiently powerful prod to shift perceptions of source credibility. Our findings also relate to an emerging body of research on accuracy motives among social media users and attempts to encourage discernment in news-sharing behavior via “accuracy nudges” (*50*). Although real-world experimentation points to the efficacy of these interventions, our null findings raise the possibility that accuracy mechanisms may operate differently for publicly observable sharing behavior than for private consumption. For example, sharing false information may come with a perceived reputational cost that is not associated with low-quality news consumption (*51*).

Although we do not uncover statistically significant average treatment effects—potentially because of limited exposure to low-credibility sites to begin with (*52*)—we present suggestive evidence of a substantively meaningful boost in news quality among the heaviest consumers of misinformation in our sample (who comprise a small proportion of respondents, approximately 10%, consistent with prior research on fake news exposure). In both the treatment period (before July 1) and in the posttreatment period (beginning July 1), we observed the quality of news diets among the lowest 10% of our sample increase by 5.4 and 8.6% from their pretreatment levels, respectively. For the period beginning July 1, we even observe a statistically significant increase in news reliability among those in the bottom 20% of pretreatment news consumption quality.

NewsGuard is not the only web extension that offers expert ratings, but it is likely the most comprehensive and transparent. Other web extensions, such as Nobias and Media Bias/Fact Check, do provide similar levels of coverage online but report ideological bias rather than reliability. Other extensions such as Newstrition and The Factual provide article and source-level ratings, but these only appear in social media feeds and not in search engine results. Given these considerations, it seems likely that other extensions would not produce larger treatment effects than those we estimate for NewsGuard.

Our findings illustrate the challenge of studying interventions arguably aimed at a specific (and often difficult-to-sample) population, namely, the heaviest consumers of misinformation. Although we chose to optimize our sampling for maximal treatment compliance, this came with a trade-off in the form of lower prevalence of the target behavior. Future research should consider the costs and benefits of other strategies, such as oversampling from the desired population to increase statistical precision. Our results are best interpreted as population-level estimates of the treatment effect, which remains a parameter of great interest to policy-makers, platforms, and designers of digital tools alike.

## MATERIALS AND METHODS

### NewsGuard extension and ratings

To produce credibility ratings, NewsGuard employs a team of trained journalists and editors to review and rate news and information websites on the basis of nine journalistic criteria. The criteria assess basic practices of reliability and transparency. On the basis of a site’s performance on these nine criteria, it is assigned a reliability rating from 0 to 100. The criteria are the following: “Does not repeatedly publish false content,” “Gathers and presents information responsibly,” “Regularly corrects or clarifies errors,” “Handles the difference between news and opinion responsibly,” “Avoids deceptive headlines,” “Website discloses ownership and financing,” “Clearly labels advertising,” “Reveals who’s in charge, including any possible conflicts of interest,” and “The site provides names of content creators, along with either contact or biographical information.”

Online domains with score of 60 or higher are considered reliable (green shield), while scores below 60 are considered unreliable. More than 41% of the more than 5000 news domains rated received a red suspect rating. Reassuringly, the NewsGuard list contains most of the fake news publishers identified by Allcott *et al*. (*53*); 88% of the online news domains in this list are rated as unreliable by NewsGuard. In addition, 99% of the mainstream online news domains identified by Microsoft Project Ratio are rated as reliable by NewsGuard. A histogram of NewsGuard scores for most online news domains can be found in section SB. Over the course of 2020, NewsGuard rated 2144 additional news domains and partnered with the World Health Organization to report misinformation as well as flagged 371 websites that spread misinformation about COVID-19 in the first months of the pandemic. NewsGuard can be installed on all major web browsers (Safari, Microsoft Edge, Mozilla Firefox, Internet Explorer, and Google Chrome) as well as Android and iOS mobile phones. Normally, the NewsGuard extension costs $2.99 per month, but it is available for free (and bundled) with Microsoft Edge as well as to more than 200 million potential users worldwide through assorted partnerships. Currently, NewsGuard is offered for free to 30 million British Telecom internet and mobile customers, students through TurnItIn, and patrons at more than 750 libraries (including the Chicago Public Library).

### Data, sample, and measures

We conducted a two-wave online panel survey of U.S. adults through the survey company YouGov in the summer of 2020 that included an encouragement to install NewsGuard in the first wave. Respondents were selected by YouGov’s matching and weighting algorithm to approximate the demographic and political attributes of the U.S. population (32% college graduates, 45% male, median age of 50 years old; 46% identify as Democrats and 36% as Republicans). We also oversampled members of the YouGov Pulse panel, who voluntarily provide behavioral data on their online information consumption (*n* = 968) (see section SB for demographic details). Pulse panelists confidentially share visit-level data on domains and URLs of web activity, including estimated duration and time stamps, on registered desktop/laptop and mobile devices. Data from Pulse panelists in our sample composed of 11,903,134 observations collected from laptop and desktop computers via the Reality Mine app. Here, we present measures using desktop data only, given that respondents could only install NewsGuard on their desktops at the time the study ran. In Supplementary Materials section SN, we replicate Fig. 4 but use only mobile data that we collected. Secure transactions and passwords are not collected or shared with researchers, and YouGov performs a scrub of personally identifying information before delivering the data. Other work has thoroughly validated Pulse data (*39*, *54*).

The main outcome of interest is news consumed by our study participants from publishers of low-quality news sources. Using Pulse data, we calculated five distinct measures of news diet quality: the average NewsGuard reliability score for websites visited, proportion and counts of unreliable (NewsGuard score < 60) news sites visited, and proportion and counts of reliable news sites visited. We are also interested in the effect of this intervention on the dependent variables specified in our other hypotheses and research questions, including the perceived accuracy of true and false news stories, trust in media, and other possible downstream effects. We are also interested in whether effects on these variables are higher within certain subgroups, such as those who use social media more or those who have lower levels of digital literacy. Details on all of these variables are available in section SA.

### Treatment and compliance

At the beginning of the wave 1 survey, respondents were asked whether they would be willing to install an extension to their web browser, which was intended to minimize differences between the treatment and control group and in compliance. We then randomly assigned respondents in wave 1 to be encouraged to install the NewsGuard web extension. We do not find that those in the treatment and control groups were statistically different across income, race, partisanship, education, and gender (sample demographic details are presented in section SB). Those in the treatment group were slightly younger (by 2 years) and had slightly higher levels of digital literacy than the control group, but the magnitudes of these differences are small. We also found recontact rates in the treatment group and the control group to be similar (14.1% in the control group compared to 13.2% in the treatment group). We do not find notable differences in demographic characteristics between those in the control and treatment groups who did not complete wave 2. We also do not find that treatment compliance rates differ between those who completed wave 2 and those who did not (53% of respondents in the treatment group who attrited installed the NewsGuard extension, while 57% of respondents in the treatment group who did not attrite installed the extension). Details are presented in section SB. On the basis of wave 2 survey data, 94% of participants in the treatment group who installed NewsGuard felt neutral or positive toward the extension, and 41% liked the extension “a little” or “a lot.”

We define “compliance” as successfully installing and activating the NewsGuard extension (as a result of the encouragement), which we validate via a script linked at the beginning of the wave 1 survey and during the last week of the treatment period. Respondents click the verification link in both compliance checks, and they are redirected to a separate page in which we can verify whether the NewsGuard extension has been installed and is active. We record their unique ID and the result of the compliance check so we can match it to their survey responses. We did not ask early respondents of our wave 2 survey to complete the compliance check during the survey. Rather, we waited until the last week of the treatment period and sent them an email asking them to click on the verification link. Respondents who filled out the wave 2 survey in the last week of the treatment period were asked to click on the verification link at the end of their wave 2 survey. This gives us two separate compliance measures that we can use for more than 92% of our respondents. This compliance check failed about 4% of the time because of random browser or survey issues that do not appear biased in any identifiable direction. Given this, we only collected first and second compliance check data for 92% of our respondents. Notably, we find little difference in the characteristics of respondents who would successfully take the treatment (for the whole treatment period) if encouraged (“compliers”) and those who would not take the treatment if encouraged (“never-takers”). We find no statistically significant evidence that respondents who comply are very much different than those who did not comply along the dimensions of age, partisanship, gender, or race. Compliers are more likely to hold a postsecondary degree, report a higher income level, and score higher on our digital literacy scale than never-takers, but the magnitudes of these differences are small (details comparing these two groups can be found in section SB). Among the respondents for whom we have web data, compliers score higher on our digital literacy scale and consumed a higher proportion of unreliable news domains than never-takers in the pretreatment period, but, again, the magnitudes of these differences are small.

On July 1, the NewsGuard extension became a pay service with a monthly subscription fee of $2.99. At that point, those who did not sign up to purchase the extension continued to encounter shield icons next to news stories, but the color no longer reflected credibility and contextual information was no longer accessible. Given the disabling of NewsGuard’s free capabilities on July 1, respondents were effectively treated for 3 to 4 weeks. We preregistered that this disabling of NewsGuard was a part of our research design. We stated in our preregistration that if we were able to collect digital trace data after this date, then we would test whether the NewsGuard treatment caused subjects to be more likely to visit sources deemed reliable by NewsGuard or less likely to visit sources deemed unreliable by NewsGuard in the period after the treatment ended.

### Analysis

Our preregistered primary analyses are an ITT model and two CACE models using two different compliance measures, one that measures compliance solely at the beginning of the treatment period and another that measures compliance both at the beginning and end of the treatment period. We report both unadjusted (differences in means) and covariate-adjusted estimates of treatment effects for each dependent variable of interest (we use HC2 robust SEs in all analyses and report *P* values from two-tailed *t* tests). For covariate-adjusted models, we selected covariates for inclusion using lasso regressions run separately for each dependent variable. The list of pretreatment variables for possible inclusion as covariates can be found in section SA. One concern with using an ITT model is that it will understate the true effect of an intervention when some respondents do not comply with the encouragement, but we report relatively high levels of compliance (discussed at length in the previous section). Given high levels of compliance, we report the covariate-adjusted ITT effect here and the CACE using both measures of compliance in the Supplementary Materials.

For one of our non-preregistered supplementary analyses, we divide the sample according to pretreatment deciles of respondents’ average news reliability scores. We do not report, as a primary specification, multiplicative interaction models. As Hainmueller *et al*. (*55*) explain, models of this kind assume a constant change across values of the moderator and require sufficient common support. Given the discussion of the skew in NewsGuard scores, it is unlikely that our data provide common support between the treatment and moderator. In addition, as we show below, it is unlikely that any effect heterogeneity is linear. For these reasons, the visual display of our exploratory results is preferable to models that impose additional, implausible assumptions. We note that these points also apply to all of our preregistered moderation analyses given the above-noted skew.

## Supplementary Materials

### This PDF file includes:

Sections SA to SN Figs. S1 to S17 Tables S1 to S108 References
