# Supplementary material for: News credibility labels have limited average effects on news diet quality and fail to reduce misperceptions
Source: Sci Adv. 2022 May 6;8(18):eabl3844. doi: 10.1126/sciadv.abl3844 (PMC9075792; doi:10.1126/sciadv.abl3844)
Supplement: Supplementary file 1 — Sections SA to SN Figs. S1 to S17 Tables S1 to S108 References [file sciadv.abl3844_sm.pdf]

Science Advances
AAAS

Supplementary Materials for

News credibility labels have limited average effects on news diet quality and fail to reduce misperceptions

Kevin Aslett*, Andrew M. Guess, Richard Bonneau, Jonathan Nagler, Joshua A. Tucker

*Corresponding author. Email: kma412@nyu.edu

Published 6 May 2022, *Sci. Adv.* **8**, eabl3844 (2022)
DOI: 10.1126/sciadv.abl3844

This PDF file includes:

Sections SA to SN
Figs. S1 to S17
Tables S1 to S108
References

Supplementary Methods and Materials: News credibility labels have limited average effects on news diet quality and fail to reduce misperceptions

Contents

SA Study design **3**
SA.1 Dependent Variables . . . 3
SA.2 Moderators . . . 7
SA.3 List of pre-treatment covariates for possible inclusion . . . 10

SB Descriptive Statistics **11**
SB.1 Descriptive statistics for sample by treatment and control groups . . . 11
SB.2 Descriptive statistics for sample by treatment and control groups by attrition . . . 12
SB.3 Descriptive statistics for sample by compliance status . . . 13
SB.4 Descriptive Statistics for NewsGuard Ratings . . . 15

SC Results From All Covariate-Adjusted Models **16**
SC.1 Behavioral Measures (Before July 1st) . . . 16
SC.2 Behavioral Measures (After July 1st) . . . 18
SC.3 Behavioral Measures - Weighted by Duration (Before July 1st) . . . 20
SC.4 Behavioral Measures - Weighted by Duration (After July 1st) . . . 22
SC.5 Behavioral Measures - Referrals from Social Media Sites and Search Engines (Before July 1st) 24
SC.6 Behavioral Measures - Referrals from Social Media Sites and Search Engines (After July 1st) 26
SC.7 Attitudinal Measures . . . 28

SD Results From All Covariate-Unadjusted Models **42**
SD.1 Behavioral Measures (Before July 1st) . . . 42
SD.2 Behavioral Measures (After July 1st) . . . 44
SD.3 Behavioral Measures - Weighted by Duration (Before July 1st) . . . 46
SD.4 Behavioral Measures - Weighted by Duration (After July 1st) . . . 48
SD.5 Behavioral Measures - Referrals from Social Media Sites and Search Engines (Before July 1st) 50
SD.6 Behavioral Measures - Referrals from Social Media Sites and Search Engines (After July 1st) 52
SD.7 Attitudinal Measures . . . 54

SE Results From Covariate-Adjusted Model Testing Hypotheses About Moderators **58**

SF Results From Covariate-Unadjusted Model Testing Hypotheses Regarding Moderators **60**

SG NewsGuard Indicators of Source Reliability **62**

SH Minimum Detectable Effects for Covariate-adjusted ITT and CACE Models **63**

SI Effect of Intervention on Behavioral Measures Using Different Samples of Respondents **64**

SJ Distribution of Pre-Treatment Behavioral Measures **69**

SK Figure: Effect of Intervention on Attitudinal Measures **75**

SL Figure: Proportion of the average daily proportion of unreliable news viewed by the treatment group **76**

SM Balance Table and Density Plots **77**

SN Results From Covariate-Adjusted Models using Mobile Behavioral Data **82**

SA Study design

SA.1 Dependent Variables

(1) Belief in misinformation and true information:

We test whether those in the treatment group are less likely to believe three popular false statements about the Black Lives Matter movement and three popular false statements about Covid-19. We also test if the respondents were more likely to believe two popular true statements the Black Lives Matter movement and true popular statements about Covid-19. To construct this measure, we asked respondents to evaluate the accuracy of a number of headlines on the following 4-point scale:

Possible answers:
(A) Not at all accurate (1)
(B) Not very accurate (2)
(C) Somewhat accurate (3)
(D) Very accurate (4)
(E) Don't know (NA)

We used two sets of items. One set (Covid-19 misinformation/information) consisted of older, popular claims about Covid-19 circulated before (within 3 months of the beginning of the treatment period) and during the treatment period. The misinformation about Covid-19 was collected from NewsGuard's own newsletter, which documented these false claims as among the most popular at the time. The accurate information was chosen by the researchers from widely shared facts about Covid-19.

The second set consisted of popular claims about the Black Lives Matter movement that started to be spread during the treatment period. The misinformation about the Black Lives Matter was selected by the researchers looking through popular claims about Black Lives Matter fact-checked by one of the following fact-checking organizations: Snopes, Politifact, Reuters, FactCheck.org, and the Associated Press. True statements were chosen by the researchers from popular news during that period.

It is possible that participants were more likely exposed to the Black Lives Matter information/misinformation relative to the Covid-19 information/misinformation, but we have no reason to believe that the claims about Covid-19 weren't circulating during the treatment period.

All of the pieces of misinformation were rated as false by at least one third-party fact-checking organization. The order of the headlines was randomized within wave for each respondent. All of the headlines are listed below:

(1.1) Information about the Black Lives Matter movement (True and False news)

We asked our respondents in the wave 2 survey: Below are statements people have been making about the Black Lives Matter movement and related protests. Please indicate whether you believe the following statements are accurate or not.

(1) Many of these protesters were paid to attend the protest. (False)
A number of fact-checks debunked this popular false claim (click on the hyperlinks to be directed to the URL):
Snopes ; Politifact ; Reuters

(2) The 75-year old man shown on video being pushed to the ground by police in Buffalo, New York was a member of Antifa. (False)
A number of fact-checks debunked this popular false claim (click on the hyperlinks to be directed to the URL):

Politifact ; Factcheck ; AP News

(3) No active police officer deaths have been connected to violence during protests. (True)
The true information can be found here (click on the hyperlinks to be directed to the URL):
Politifact

(4) Donald Trump mocked George Floyd by saying "I can't breathe" at a recent rally. (False)
A number of fact-checks debunked this popular false claim (click on the hyperlinks to be directed to the URL):
Politifact ; Reuters

(5) In response to the Black Lives Matter movement, NASCAR has banned Confederate flags at all races. (True)
The true information can be found here (click on the hyperlinks to be directed to the URL):
CNN ; NPR

(1.2) Information about Covid-19 (True and False news)

Below are statements people have been making about Covid-19 (the novel coronavirus). Please indicate whether you believe the following statements are accurate or not.

(1) Covid-19 is being spread by 5G cell phone technology. (False)
A number of fact-checks debunked this popular false claim (click on the hyperlinks to be directed to the URL):
Reuters ; Fullfact ; BBC

(2) The Bill and Melinda Gates Foundation is planning to implement a mandatory Covid-19 vaccine program that utilizes microchips under a patient's skin. (False) A number of fact-checks debunked this popular false claim (click on the hyperlinks to be directed to the URL):
Factcheck ; Factcheck ; Nature

(3) A new loss of taste or smell is a symptom of Covid-19. (True)
The true information can be found here (click on the hyperlinks to be directed to the URL):
Mayo Clinic ; Healthline

(4) The Chinese government created Covid-19 as a bioweapon. (False)
A number of fact-checks debunked this popular false claim (click on the hyperlinks to be directed to the URL):
Factcheck ; Buzzfeed News

(5) Covid-19 can be spread by people who do not show symptoms. (True)
The true information can be found here (click on the hyperlinks to be directed to the URL):
Nebraskamed ; CDC

(2) Trust in media:

Media Trust Index (Wave 1: $Media\_Trust\_Index_i$ **; Wave 2:** $Media\_Trust\_Index\_2_i$**):** Measures the respondent's trust in media using the summed value assigned to the respondent's answer to the next three questions. To create an index for media trust we sum the values assigned to each respondent's answer for each question. The sum is assigned to the following variables: $Media\_Trust\_Index_i$ (wave 1) and $Media\_Trust\_Index\_2_i$ (wave 2):

Some people think that by criticizing leaders, news organizations keep political leaders from doing their job. Others think that such criticism is worth it because it keeps political leaders from doing things that should not be done. Which position is closer to your opinion?

(A) Stops political leaders from doing their job (0)
(B) Keep political leaders from doing things that shouldn't be done (1)

In presenting the news dealing with political and social issues, do you think that news organizations deal fairly with all sides, or do they tend to favor one side?

(A) Deal fairly with all sides (1)
(B) Tend to favor one side (0)

Based on what you know, how often do you believe the nation's major news organizations fabricate news stories?

(A) All the time (0)
(B) Most of the time (0.25)
(C) About half the time (0.5)
(D) Once in a while (0.75)
(E) Never (1)

The first question is pulled from the Pew American Trends Panel survey (*55*). The second two questions are taken from (*56*).

(3) Trust in specific mainstream online news producers

This question measures the level of trust in the following media sources. The value assigned to each respondent is in parentheses next to the answer. Higher values denotes higher levels of trust.

How much, if at all, do you trust the information you get from:
(1) Fox News
(2) CNN
(3) MSNBC
(4) CBS
(5) ABC
(6) NBC

Possible Answers:
(A) A lot (4)
(B) Some (3)
(C) Not too much (2)
(D) Not at all (1)

(4) Affective polarization

Measures affective polarization by taking the absolute value of the difference between the feeling thermometers of each political party taken during each wave. Equation: $dem\_feeling\_thermometer_i$ - $rep\_feeling\_thermometer_i$. Below is the question asked to create this measure.

Feeling Towards Democratic and Republican Party (Wave 1: $dem\_feeling\_thermometer_i$**,** $rep\_feeling\_thermometer$ **Wave 2:** $dem\_feeling\_thermometer\_2_i$, $rep\_feeling\_thermometer\_2_i$**):** Measure the respondent's the positive feeling towards each party on a thermometer scale of 0 (negative) to 100 (positive). This is one measure

of affective polarization. The value of the thermometer is assigned to each respective variable.

(1) How would you rate the Democratic Party?

(2) How would you rate the Republican Party?

This method is the same method used to calculate affective polarization in the ANES survey.

(5) Political cynicism

Measures the respondent's level of political cynicism. The value assigned to each respondent is in parentheses next to the answer. Higher values denotes higher levels of political cynicism.

Do you think that quite a few of the people running the government are crooked, not very many are, or do you think hardly any of them are crooked?
(A) Quite a few (3)
(B) Not very many (2)
(C) Hardly any (1)
(D) Don't Know (NA)

This questions is the same used by (*55*) and ANES.

(6) Trust in institutions

Trust: Trust in institutions is measured using four questions from the ANES survey that measures trust. For each question a respondent is assigned a value between 0 and 1 dependent on their answer. The values they are assigned for each answer are in parentheses.

How much of the time do you think you can trust each of the following groups to do what is right?
Groups: "The federal government in Washington D.C." ; "Law enforcement" ; "The media" ; "People in general"
Possible Answers: "Almost never" (0) ; "Some of the time" (0.33) ; "Most of the time" (0.67) ; "Almost Always" (1.0)

How much do public officials care what people like you think?
(A) None at all (0)
(B) A little (0.25)
(C) A moderate amount (0.50)
(D) A lot (0.75)
(E) A great deal (1.0)

Do you think the government today has:
(A) Too little power (0)
(B) About the right amount of power (0.5)
(C) Too much power. (1.0)

How much can people like you affect what the government does?
(A) None at all (0)
(B) A little (0.25)
(C) A moderate amount (0.50)

(D) A lot (0.75)
(E) A great deal (1.0)

$Trust\_Score_i$ is the average value each respondent received.

This method of measurement is the same as that used in the ANES and (*56*).

(7) Belief that fake news is a problem in general

We assign a value to the variables, $Fake\_news\_problem_i$ (Wave 1) and $Fake\_news\_problem\_2_i$ (Wave 2), depending on the value to the answer they choose for the question below.

How much of a problem do you think made-up news and information is in the country today?

(A) 4: A very big problem (4)
(B) 3 (3)
(C) 2 (2)
(D) 1, Not a problem at all (1)

(8) Belief that fake news is a problem in the mainstream media.

We assign a value to the variables, $Fake\_news\_MM_i$ (Wave 1) and $Fake\_news\_MM\_2_i$ (Wave 2), depending on the value to the answer they choose for the question below.

President Trump often accuses several major news outlets of being "fake news." Do you think "fake news" from the mainstream media is a real problem, or do you think the mainstream media are generally reliable and report the facts as best they can?

(A) 5: "Fake news" is a serious problem affecting the mainstream media. (5)
(B) 4 (4)
(C) 3 (3)
(D) 2 (2)
(E) 1: Mistakes by the mainstream media are rare, and I generally find what they report to be credible. (1)

SA.2 Moderators

(1) Social media use

We measure the respondent's activity on Twitter and Facebook. We ask the following question:

How frequently do you: (1) Look at Twitter
(2) Post things on Twitter
(3) Look at Facebook
(4) Post things on Facebook

Possible responses: (A) At least 10 times a day (7)
(B) Several times a day (6)
(C) About once a day (5)
(D) 3 to 6 days a week (4)

(E) 1 to 2 days a week (3)
(F) Every few weeks (2)
(G) 1 to 2 days a week (1)
(H) Never (0)
(I) Don't Know (NA)

We create an index for social media frequency by taking the average of the values assigned to the variables $facebook\_post\_frequency_i$, $twitter\_post\_frequency_i$, $twitter\_view\_frequency_i$, and $facebook\_view\_frequency_i$. The average value of these variables are assigned to the variable $Social\_Media\_Freq_i$.

(2) Digital literacy

Digital literacy is measured using the following two grid questions.

The first grid question asks for respondent's familiarity with the following terms on a five point scale (1 representing no understanding and 5 representing full understanding):
(1) Phishing
(2) JPG
(3) Cache
(4) Malware
(5) RSS
(6) Hashtag

The second grid question asks respondents' agreement with the following statements on a scale of -4 = Strongly Disagree to 4 = Strongly Agree:
(1) I prefer to ask friends how to use any new technological gadget instead of trying to figure it out myself.
(2) I feel like information technology is a part of my daily life.
(3) Using information technology makes it easier to do my work.
(4) I often have trouble finding things that I've saved on my computer.

Note: The value for one and four are reverse coded.

By summing all of the values we can create a digital literacy score that is assigned to the variable: DL_Score_i for each respondent. We take the inverse to determine if the effect size increased with lower levels of digital literacy.

(3) Online news consumption

The number of non-successive domains with NewsGuard scores before the respondent took the wave 1 survey (from May 15th to the first day of wave 1). The sum of non-successive domains with NewsGuard scores after the treatment.

(4) Partisan news diet

Using web tracking data from before individuals are treated we analyze web tracking data. We average the partisanship scores from domains visited to create a measure of news diet partisanship. The partisanship scores are derived from (*57*) and each domain visited if in this list is given a partisanship score. If it is not in the list it is not used. The average partisanship score is assigned to the variable $partisanship\_new\_diet_i$.

(5) Prior visits to online publishers of fake news

The sum of the $domain\_unreliable\_dummy_j$ scores assigned to domains visited by the respondent divided by number of non-successive domains with NewsGuard scores before the respondent took the wave 1 survey.

(6) Those who use the Safari web browser

A dummy variable that is assigned "1" if the browser the respondent is using the Safari browser to take the survey and possibly download the extension (Yes: 1 ; No: 0).

SA.3 List of pre-treatment covariates for possible inclusion

Our list of pre-treatment covariates for possible inclusion are: gender ($Female_i$ dummy), education level ($Education_i$), age (Age_i), age squared (Age_i^2), 7-point party id ($Party\_ID_i$), race/ethnicity ($Ethnicity_i$), 7-point ideology ($Con\_Ideology_i$), media trust ($Media\_Trust\_Index_i$), social media use ($Social\_Media\_Freq_i$), trust in information from newspapers ($trust\_newspapers_i$), trust in information from social media ($trust\_socmed_i$), news consumption ($trad\_news\_network_i$, $trad\_news\_cable_i$, $trad\_news\_print_i$, $trad\_news\_public_i$, $trad\_news\_talk_i$, $trad\_news\_desk_i$, $trad\_news\_mobile_i$), browser used, pre-treatment version of the DV (if available and not already listed), logged number of visits to domains with NewsGuard scores (if DV is a Pulse count).

SB Descriptive Statistics

SB.1 Descriptive statistics for sample by treatment and control groups

Table S1: Below, we present the summary statistic for those who completed both waves and those who only completed the first wave.

Group	Observ.	Age	Dig. Lit.	Income	Gender (Prop. Female)	Race (Prop. White)	Education	Ideology
Control and did not take the Wave 2 survey	220	48.99	48.21	6.23	0.61	0.76	3.86	-0.39
Control and took both surveys	1339	56.73	47.11	6.64	0.54	0.80	4.02	-0.19
Treated and did not take the Wave 2 survey	304	48.39	47.11	6.55	0.58	0.79	4.00	-0.46
Treated and took both surveys	1998	54.55	47.92	6.44	0.56	0.80	4.04	-0.28

SB.2 Descriptive statistics for sample by treatment and control groups by attrition

Table S2: Below, we present the summary statistic for groups that completed both waves and those that only completed one wave. 14.1% of respondents in the control group did not take the second wave survey. 13.2% of respondents in the treatment group did not take the second wave survey.

Group	Observ.	Age	Dig. Lit.	Income	Gender (Prop. Female)	Race (Prop. White)	Education	Ideology
Control and we do not have digital trace data	1035	55.51	46.73	6.81	0.55	0.78	4.00	3.76
Control and we have digital trace data	356	60.07	48.17	6.19	0.51	0.86	4.06	3.94
Treated and we do not have digital trace data	1386	52.83	48.35	6.50	0.54	0.78	4.02	3.67
Treated and we have digital trace data	612	58.44	46.96	6.30	0.60	0.86	4.06	3.81

SB.3 Descriptive statistics for sample by compliance status

In the following figures, we show estimated means along various dimensions for respondents who would install the NewsGuard web browser extension if and only if they are assigned to receive it ("compliers") and those who would not under any circumstances ("never-takers") computed following the procedure in (*58*). Points show estimated means for the complete sample, respondents who would take the treatment only if encouraged to install it ("compliers"), and respondents who would not take the treatment even if encouraged to install it ("never-taker") computed following (*58*). All variables are rescaled to the 0 to 1 interval. Lines display 95% confidence intervals based on bootstrapped standard errors. Asterisks on variable names indicates that the mean values for compliers and never-takers are significantly different ($p < .05$).

Figure S1: Profile of all compliers and never-takers

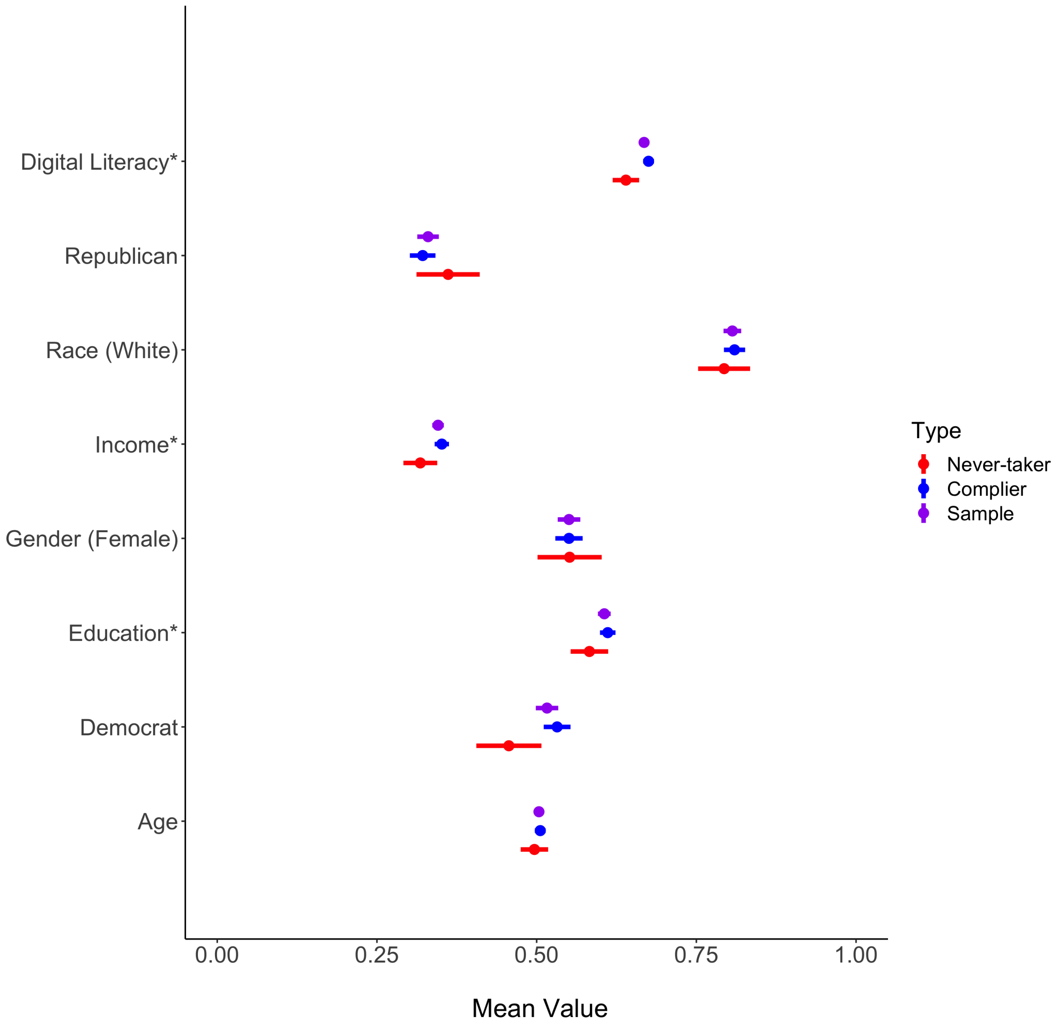

Figure S2: Profile of compliers and never-takers with whom we collect web-browsing data

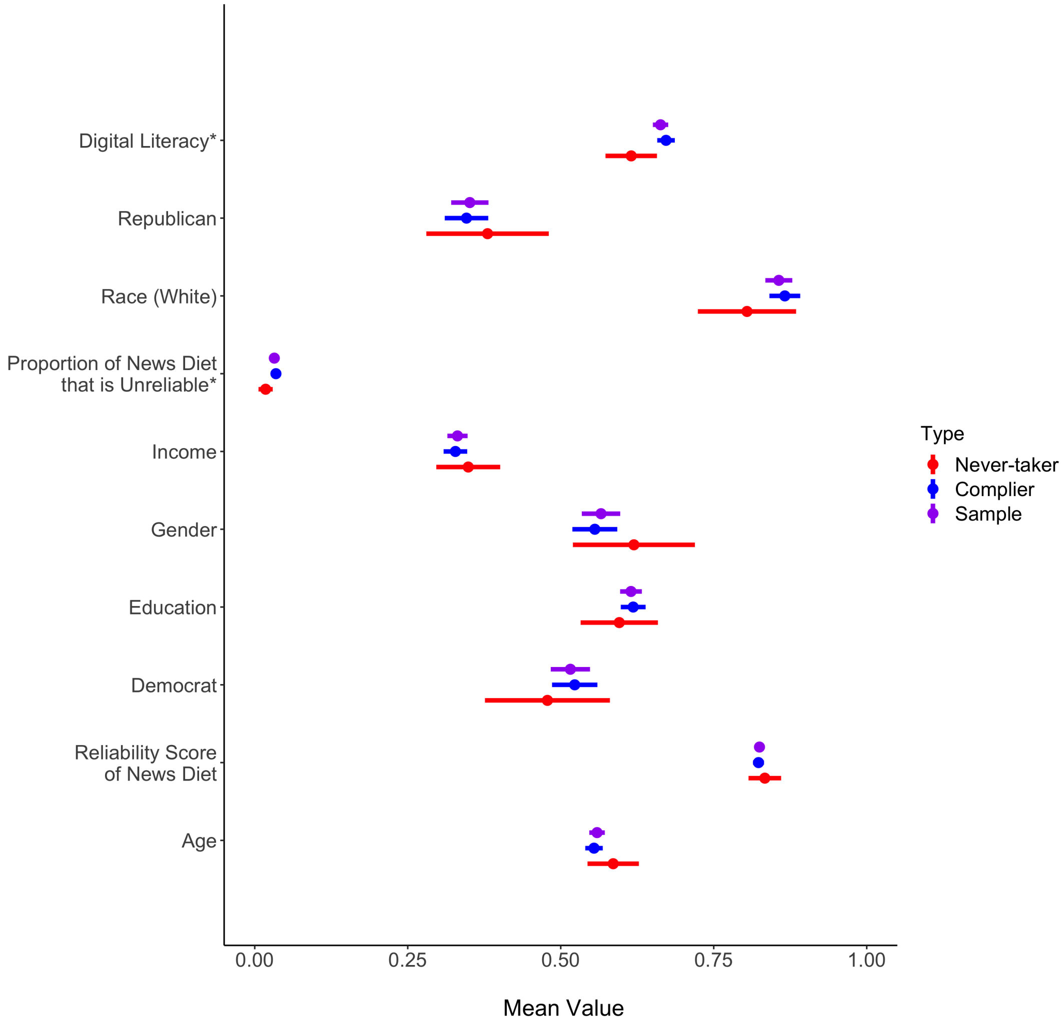

SB.4 Descriptive Statistics for NewsGuard Ratings

Below we provide a histogram of domain reliability scores represented in the NewsGuard database (i.e., not weighted by visits from participants in our sample):

Figure S3: Histogram of NewsGuard Scores

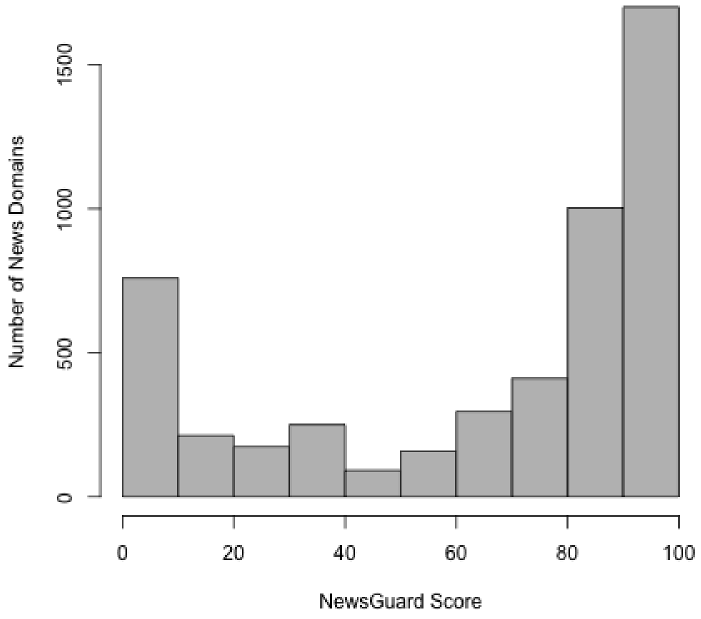

SC Results From All Covariate-Adjusted Models

For covariate-adjusted models, we selected covariates for inclusion using lasso with default options in glmnet and a seed set to 938. We ran this procedure separately for each dependent variable and use the same selected variables for both OLS (ITT) and 2SLS (CACE) models. Our list of pre-treatment covariates for possible inclusion can be found in section A3 of this supplementary methods and materials.

SC.1 Behavioral Measures (Before July 1st)

Table S3: Testing the Effect of the Intervention on Proportion of News Diet That is Unreliable with Covariate-Adjusted Models (HC2 Robust standard errors) (Before July 1st)

	Intent-To-Treat (ITT)	CACE (Model 1)	CACE (Model 2)
Treatment	−0.0019	−0.0022	−0.0025
	(0.0028)	(0.0030)	(0.0035)
Pre-Treatment Value	0.8631***	0.8642***	0.8571***
	(0.0508)	(0.0511)	(0.0533)
R^2	0.8212	0.8211	0.8137
Adj. R^2	0.8208	0.8207	0.8132
Num. obs.	821	806	786

$^{***}p < 0.001$; $^{**}p < 0.01$; $^{*}p < 0.05$

Table S4: Testing the Effect of the Intervention on Proportion of News Diet That is Reliable with Covariate-Adjusted Models (HC2 Robust standard errors) (Before July 1st)

	Intent-To-Treat (ITT)	CACE (Model 1)	CACE (Model 2)
Treatment	−0.0075	−0.0065	−0.0060
	(0.0125)	(0.0132)	(0.0150)
Pre-Treatment Value	0.7992***	0.7958***	0.8034***
	(0.0226)	(0.0231)	(0.0229)
R^2	0.6614	0.6552	0.6662
Adj. R^2	0.6605	0.6544	0.6653
Num. obs.	821	806	786

$^{***}p < 0.001$; $^{**}p < 0.01$; $^{*}p < 0.05$

Table S5: Testing the Effect of the Intervention on Count of Unreliable News Consumed with Covariate-Adjusted Models (HC2 Robust standard errors) (Before July 1st)

	Intent-To-Treat (ITT)	CACE (Model 1)	CACE (Model 2)
Treatment	0.0409	0.0443	0.0500
	(0.0216)	(0.0227)	(0.0263)
Age-Squared	0.0000***	0.0000***	0.0000***
	(0.0000)	(0.0000)	(0.0000)
Pre-Treatment Value	0.8420***	0.8422***	0.8346***
	(0.0261)	(0.0266)	(0.0271)
Log of news viewed	0.0386***	0.0386***	0.0399***
	(0.0061)	(0.0062)	(0.0064)
R^2	0.7246	0.7214	0.7156
Adj. R^2	0.7233	0.7201	0.7142
Num. obs.	854	838	814

$^{***}p < 0.001$; $^{**}p < 0.01$; $^{*}p < 0.05$

Table S6: Testing the Effect of the Intervention on Count of Reliable News Consumed with Covariate-Adjusted Models (HC2 Robust standard errors) (Before July 1st)

	Intent-To-Treat (ITT)	CACE (Model 1)	CACE (Model 2)
Treatment	−0.0100	−0.0100	−0.0091
	(0.0194)	(0.0205)	(0.0234)
Age	0.0048***	0.0049***	0.0046***
	(0.0008)	(0.0008)	(0.0008)
Pre-Treatment Value	0.5013***	0.5019***	0.4968***
	(0.0367)	(0.0376)	(0.0382)
Log of news viewed	0.2079***	0.2075***	0.2109***
	(0.0170)	(0.0175)	(0.0179)
R^2	0.8668	0.8643	0.8650
Adj. R^2	0.8661	0.8636	0.8643
Num. obs.	854	838	814

$^{***}p < 0.001$; $^{**}p < 0.01$; $^{*}p < 0.05$

Table S7: Testing the Effect of the Intervention on Reliability Score of News Diet with Covariate-Adjusted Models (HC2 Robust standard errors) (Before July 1st)

	Intent-To-Treat (ITT)	CACE (Model 1)	CACE (Model 2)
Treatment	0.4702	0.5048	0.5649
	(0.3221)	(0.3346)	(0.3853)
Pre-Treatment Value	0.8184***	0.8227***	0.8213***
	(0.0357)	(0.0351)	(0.0367)
R^2	0.6676	0.6738	0.6657
Adj. R^2	0.6668	0.6730	0.6649
Num. obs.	821	806	786

$^{***}p < 0.001$; $^{**}p < 0.01$; $^{*}p < 0.05$

SC.2 Behavioral Measures (After July 1st)

Table S8: Testing the Effect of the Intervention on Proportion of News Diet That is Unreliable with Covariate-Adjusted Models (HC2 Robust standard errors) (After July 1st)

	Intent-To-Treat (ITT)	CACE (Model 1)	CACE (Model 2)
Treatment	-0.0043	-0.0043	-0.0031
	(0.0035)	(0.0037)	(0.0042)
Pre-Treatment Value	0.7979^{***}	0.7984^{***}	0.8222^{***}
	(0.0619)	(0.0622)	(0.0596)
R^2	0.7465	0.7462	0.7621
Adj. R^2	0.7459	0.7455	0.7615
Num. obs.	763	750	729

$^{***}p < 0.001$; $^{**}p < 0.01$; $^{*}p < 0.05$

Table S9: Testing the Effect of the Intervention on Proportion of News Diet That is Reliable with Covariate-Adjusted Models (HC2 Robust standard errors) (After July 1st)

	Intent-To-Treat (ITT)	CACE (Model 1)	CACE (Model 2)
Treatment	-0.0013	-0.0005	-0.0011
	(0.0142)	(0.0149)	(0.0168)
Pre-Treatment Value	0.7733^{***}	0.7727^{***}	0.7869^{***}
	(0.0247)	(0.0253)	(0.0247)
R^2	0.6084	0.6032	0.6201
Adj. R^2	0.6074	0.6021	0.6191
Num. obs.	763	750	729

$^{***}p < 0.001$; $^{**}p < 0.01$; $^{*}p < 0.05$

Table S10: Testing the Effect of the Intervention on Count of Unreliable News Consumed with Covariate-Adjusted Models (HC2 Robust standard errors) (After July 1st)

	Intent-To-Treat (ITT)	CACE (Model 1)	CACE (Model 2)
Treatment	0.0062	0.0062	0.0141
	(0.0192)	(0.0202)	(0.0233)
Pre-Treatment Value	0.7034^{***}	0.7080^{***}	0.7047^{***}
	(0.0291)	(0.0298)	(0.0306)
R^2	0.6546	0.6525	0.6498
Adj. R^2	0.6538	0.6517	0.6489
Num. obs.	846	829	805

$^{***}p < 0.001$; $^{**}p < 0.01$; $^{*}p < 0.05$

Table S11: Testing the Effect of the Intervention on Count of Reliable News Consumed with Covariate-Adjusted Models (HC2 Robust standard errors) (After July 1st)

	Intent-To-Treat (ITT)	CACE (Model 1)	CACE (Model 2)
Treatment	-0.0240	-0.0267	-0.0372
	(0.0314)	(0.0330)	(0.0373)
Pre-Treatment Value	0.5667^{***}	0.5662^{***}	0.5724^{***}
	(0.0489)	(0.0499)	(0.0512)
Log of news viewed	0.1426^{***}	0.1427^{***}	0.1447^{***}
	(0.0224)	(0.0229)	(0.0236)
R^2	0.7180	0.7146	0.7228
Adj. R^2	0.7169	0.7136	0.7217
Num. obs.	811	797	774

$^{***}p < 0.001$; $^{**}p < 0.01$; $^{*}p < 0.05$

Table S12: Testing the Effect of the Intervention on Reliability Score of News Diet with Covariate-Adjusted Models (HC2 Robust standard errors) (After July 1st)

	Intent-To-Treat (ITT)	CACE (Model 1)	CACE (Model 2)
Treatment	0.5667	0.5644	0.5934
	(0.3577)	(0.3722)	(0.4201)
Pre-Treatment Value	0.7583^{***}	0.7705^{***}	0.7845^{***}
	(0.0428)	(0.0423)	(0.0402)
R^2	0.6095	0.6206	0.6288
Adj. R^2	0.6085	0.6196	0.6278
Num. obs.	763	750	729

$^{***}p < 0.001$; $^{**}p < 0.01$; $^{*}p < 0.05$

SC.3 Behavioral Measures - Weighted by Duration (Before July 1st)

Table S13: Testing the Effect of the Intervention on Proportion of News Diet That is Unreliable with Covariate-Adjusted Models (HC2 Robust standard errors) (Before July 1st) – Duration Weighted

	Intent-To-Treat (ITT)	CACE (Model 1)	CACE (Model 2)
Treatment	−0.0048	−0.0049	−0.0056
	(0.0036)	(0.0038)	(0.0045)
Pre-Treatment Value	0.8476***	0.8481***	0.8406***
	(0.0521)	(0.0522)	(0.0545)
R^2	0.7541	0.7537	0.7433
Adj. R^2	0.7535	0.7531	0.7427
Num. obs.	821	806	786

$^{***}p < 0.001$; $^{**}p < 0.01$; $^{*}p < 0.05$

Table S14: Testing the Effect of the Intervention on Proportion of News Diet That is Reliable with Covariate-Adjusted Models (HC2 Robust standard errors) (Before July 1st) – Duration Weighted

	Intent-To-Treat (ITT)	CACE (Model 1)	CACE (Model 2)
Treatment	0.0013	0.0027	0.0007
	(0.0151)	(0.0158)	(0.0182)
Pre-Treatment Value	0.7521***	0.7475***	0.7483***
	(0.0233)	(0.0237)	(0.0244)
R^2	0.5892	0.5824	0.5805
Adj. R^2	0.5882	0.5814	0.5795
Num. obs.	821	806	786

$^{***}p < 0.001$; $^{**}p < 0.01$; $^{*}p < 0.05$

Table S15: Testing the Effect of the Intervention on Count of Unreliable News Consumed with Covariate-Adjusted Models (HC2 Robust standard errors) (Before July 1st) – Duration Weighted

	Intent-To-Treat (ITT)	CACE (Model 1)	CACE (Model 2)
Treatment	−263.1468	−286.1545	−330.9852
	(159.9564)	(171.2872)	(199.9503)
Pre-Treatment Value	0.9670***	0.9680***	0.9683***
	(0.2059)	(0.2069)	(0.2076)
R^2	0.7408	0.7411	0.7410
Adj. R^2	0.7402	0.7405	0.7404
Num. obs.	891	872	848

$^{***}p < 0.001$; $^{**}p < 0.01$; $^{*}p < 0.05$

Table S16: Testing the Effect of the Intervention on Count of Reliable News Consumed with Covariate-Adjusted Models (HC2 Robust standard errors) (Before July 1st) – Duration Weighted

	Intent-To-Treat (ITT)	CACE (Model 1)	CACE (Model 2)
Treatment	971.1443	923.8793	1275.1953
	(2127.7790)	(2257.1809)	(2644.3343)
Pre-Treatment Value	1.0578^{***}	1.0576^{***}	1.0567^{***}
	(0.0814)	(0.0814)	(0.0815)
R^2	0.7071	0.7070	0.7064
Adj. R^2	0.7065	0.7064	0.7057
Num. obs.	891	872	848

$^{***}p < 0.001$; $^{**}p < 0.01$; $^{*}p < 0.05$

Table S17: Testing the Effect of the Intervention on Reliability Score of News Diet with Covariate-Adjusted Models (HC2 Robust standard errors) (Before July 1st) – Duration Weighted

	Intent-To-Treat (ITT)	CACE (Model 1)	CACE (Model 2)
Treatment	0.5953	0.6506	0.6352
	(0.4026)	(0.4217)	(0.4853)
Party ID	-0.2751^{*}	-0.2491^{*}	-0.2762^{*}
	(0.1245)	(0.1240)	(0.1260)
Ideology	-0.2606	-0.2860	-0.2650
	(0.1488)	(0.1466)	(0.1494)
Pre-Treatment Value	0.7466^{***}	0.7497^{***}	0.7469^{***}
	(0.0464)	(0.0466)	(0.0481)
R^2	0.5843	0.5867	0.5778
Adj. R^2	0.5823	0.5846	0.5756
Num. obs.	821	806	786

$^{***}p < 0.001$; $^{**}p < 0.01$; $^{*}p < 0.05$

SC.4 Behavioral Measures - Weighted by Duration (After July 1st)

Table S18: Testing the Effect of the Intervention on Proportion of News Diet That is Unreliable with Covariate-Adjusted Models (HC2 Robust standard errors) (After July 1st) – Duration Weighted

	Intent-To-Treat (ITT)	CACE (Model 1)	CACE (Model 2)
Treatment	−0.0060	−0.0065	−0.0055
	(0.0046)	(0.0049)	(0.0056)
Pre-Treatment Value	0.8418***	0.8438***	0.8648***
	(0.0577)	(0.0577)	(0.0563)
R^2	0.7064	0.7076	0.7158
Adj. R^2	0.7056	0.7068	0.7150
Num. obs.	761	748	727

$^{***}p < 0.001$; $^{**}p < 0.01$; $^{*}p < 0.05$

Table S19: Testing the Effect of the Intervention on Proportion of News Diet That is Reliable with Covariate-Adjusted Models (HC2 Robust standard errors) (After July 1st) – Duration Weighted

	Intent-To-Treat (ITT)	CACE (Model 1)	CACE (Model 2)
Treatment	0.0011	0.0042	0.0059
	(0.0176)	(0.0186)	(0.0210)
Pre-Treatment Value	0.6997***	0.6945***	0.7142***
	(0.0283)	(0.0288)	(0.0286)
R^2	0.5011	0.4939	0.5142
Adj. R^2	0.4998	0.4925	0.5128
Num. obs.	761	748	727

$^{***}p < 0.001$; $^{**}p < 0.01$; $^{*}p < 0.05$

Table S20: Testing the Effect of the Intervention on Count of Unreliable News Consumed with Covariate-Adjusted Models (HC2 Robust standard errors) (After July 1st) – Duration Weighted

	Intent-To-Treat (ITT)	CACE (Model 1)	CACE (Model 2)
Treatment	−123.5642	−124.8044	−140.8097
	(162.4097)	(171.1623)	(201.6918)
R^2	0.0010	0.0005	−0.0004
Adj. R^2	−0.0002	−0.0006	−0.0016
Num. obs.	884	867	839

$^{***}p < 0.001$; $^{**}p < 0.01$; $^{*}p < 0.05$

Table S21: Testing the Effect of the Intervention on Count of Reliable News Consumed with Covariate-Adjusted Models (HC2 Robust standard errors) (After July 1st) – Duration Weighted

	Intent-To-Treat (ITT)	CACE (Model 1)	CACE (Model 2)
Treatment	2073.2925	2200.9569	2575.8879
	(1475.2341)	(1566.0089)	(1842.3836)
Pre-Treatment Value	0.4019***	0.4015***	0.4009***
	(0.1035)	(0.1037)	(0.1039)
R^2	0.5724	0.5722	0.5721
Adj. R^2	0.5714	0.5712	0.5711
Num. obs.	846	829	805

$^{***}p < 0.001$; $^{**}p < 0.01$; $^{*}p < 0.05$

Table S22: Testing the Effect of the Intervention on Reliability Score of News Diet with Covariate-Adjusted Models (HC2 Robust standard errors) (After July 1st) – Duration Weighted

	Intent-To-Treat (ITT)	CACE (Model 1)	CACE (Model 2)
Treatment	0.6736	0.7206	0.7293
	(0.4559)	(0.4726)	(0.5387)
Pre-Treatment Value	0.7395***	0.7531***	0.7561***
	(0.0509)	(0.0507)	(0.0522)
R^2	0.5309	0.5440	0.5411
Adj. R^2	0.5297	0.5427	0.5399
Num. obs.	761	748	727

$^{***}p < 0.001$; $^{**}p < 0.01$; $^{*}p < 0.05$

SC.5 Behavioral Measures - Referrals from Social Media Sites and Search Engines (Before July 1st)

Table S23: Testing the Effect of the Intervention on Proportion of News Diet of Referrals From Search Engines and Social Media (Google, Twitter, and Facebook) That is Unreliable with Covariate-Adjusted Models (HC2 Robust standard errors) (Before July 1st)

	Intent-To-Treat (ITT)	CACE (Model 1)	CACE (Model 2)
Treatment	−0.0035	−0.0046	−0.0055
	(0.0068)	(0.0072)	(0.0083)
Pre-Treatment Value	0.6561***	0.6589***	0.6272***
	(0.0933)	(0.0938)	(0.0959)
R^2	0.4071	0.4089	0.3717
Adj. R^2	0.4053	0.4070	0.3696
Num. obs.	649	638	620

$^{***}p < 0.001$; $^{**}p < 0.01$; $^{*}p < 0.05$

Table S24: Testing the Effect of the Intervention on Proportion of News Diet of Referrals From Search Engines and Social Media (Google, Twitter, and Facebook) That is Reliable with Covariate-Adjusted Models (HC2 Robust standard errors) (Before July 1st)

	Intent-To-Treat (ITT)	CACE (Model 1)	CACE (Model 2)
Treatment	−0.0295	−0.0283	−0.0351
	(0.0166)	(0.0174)	(0.0201)
Pre-Treatment Value	0.6616***	0.6568***	0.6504***
	(0.0337)	(0.0345)	(0.0360)
R^2	0.4471	0.4384	0.4275
Adj. R^2	0.4454	0.4367	0.4257
Num. obs.	649	638	620

$^{***}p < 0.001$; $^{**}p < 0.01$; $^{*}p < 0.05$

Table S25: Testing the Effect of the Intervention on Count of Unreliable News Referred From Search Engines and Social Media (Google, Twitter, and Facebook) with Covariate-Adjusted Models (HC2 Robust standard errors) (Before July 1st)

	Intent-To-Treat (ITT)	CACE (Model 1)	CACE (Model 2)
Treatment	−0.0050	−0.0078	−0.0086
	(0.0167)	(0.0174)	(0.0202)
Log of news viewed	0.0237***	0.0233***	0.0228***
	(0.0046)	(0.0047)	(0.0047)
Pre-Treatment Value	0.8034***	0.7996***	0.7902***
	(0.0525)	(0.0530)	(0.0537)
R^2	0.6074	0.6041	0.5963
Adj. R^2	0.6057	0.6024	0.5945
Num. obs.	700	689	668

$^{***}p < 0.001$; $^{**}p < 0.01$; $^{*}p < 0.05$

Table S26: Testing the Effect of the Intervention on Count of Reliable News Referred From Search Engines and Social Media (Google, Twitter, and Facebook) with Covariate-Adjusted Models (HC2 Robust standard errors) (Before July 1st)

	Intent-To-Treat (ITT)	CACE (Model 1)	CACE (Model 2)
Treatment	-0.0105	-0.0130	-0.0140
	(0.0241)	(0.0251)	(0.0290)
Log of news viewed	0.0848^{***}	0.0837^{***}	0.0846^{***}
	(0.0112)	(0.0113)	(0.0115)
Pre-Treatment Value	0.7563^{***}	0.7564^{***}	0.7626^{***}
	(0.0255)	(0.0258)	(0.0264)
R^2	0.7794	0.7781	0.7828
Adj. R^2	0.7785	0.7772	0.7818
Num. obs.	700	689	668

$^{***}p < 0.001$; $^{**}p < 0.01$; $^{*}p < 0.05$

Table S27: Testing the Effect of the Intervention on Reliability Score of News Referred From Search Engines and Social Media (Google, Twitter, and Facebook) with Covariate-Adjusted Models (HC2 Robust standard errors) (Before July 1st)

	Intent-To-Treat (ITT)	CACE (Model 1)	CACE (Model 2)
Treatment	0.8349	0.8852	0.9550
	(0.6286)	(0.6595)	(0.7581)
Pre-Treatment Value	0.5965^{***}	0.5869^{***}	0.5808^{***}
	(0.0711)	(0.0714)	(0.0742)
R^2	0.3999	0.3933	0.3792
Adj. R^2	0.3980	0.3914	0.3772
Num. obs.	649	638	620

$^{***}p < 0.001$; $^{**}p < 0.01$; $^{*}p < 0.05$

SC.6 Behavioral Measures - Referrals from Social Media Sites and Search Engines (After July 1st)

Table S28: Testing the Effect of the Intervention on Proportion of News Diet of Referrals From Search Engines and Social Media (Google, Twitter, and Facebook) That is Unreliable with Covariate-Adjusted Models (HC2 Robust standard errors) (After July 1st)

	Intent-To-Treat (ITT)	CACE (Model 1)	CACE (Model 2)
Treatment	0.0037	0.0037	0.0048
	(0.0067)	(0.0072)	(0.0083)
Pre-Treatment Value	0.6389***	0.6386***	0.6342***
	(0.0889)	(0.0891)	(0.0971)
R^2	0.4539	0.4535	0.4310
Adj. R^2	0.4519	0.4514	0.4288
Num. obs.	548	538	522

$^{***}p < 0.001$; $^{**}p < 0.01$; $^{*}p < 0.05$

Table S29: Testing the Effect of the Intervention on Proportion News Diet of Referrals From Search Engines and Social Media (Google, Twitter, and Facebook) That is Reliable with Covariate-Adjusted Models (HC2 Robust standard errors) (After July 1st)

	Intent-To-Treat (ITT)	CACE (Model 1)	CACE (Model 2)
Treatment	0.0126	0.0166	0.0102
	(0.0195)	(0.0206)	(0.0228)
Pre-Treatment Value	0.6370***	0.6310***	0.6546***
	(0.0382)	(0.0393)	(0.0396)
R^2	0.3934	0.3832	0.4026
Adj. R^2	0.3912	0.3809	0.4003
Num. obs.	548	538	522

$^{***}p < 0.001$; $^{**}p < 0.01$; $^{*}p < 0.05$

Table S30: Testing the Effect of the Intervention on Count of Unreliable News Referred From Search Engines and Social Media (Google, Twitter, and Facebook) with Covariate-Adjusted Models (HC2 Robust standard errors) (After July 1st)

	Intent-To-Treat (ITT)	CACE (Model 1)	CACE (Model 2)
Treatment	0.0128	0.0097	0.0156
	(0.0159)	(0.0166)	(0.0193)
Pre-Treatment Value	0.5838***	0.5868***	0.5773***
	(0.0490)	(0.0499)	(0.0514)
R^2	0.5015	0.5028	0.4885
Adj. R^2	0.4999	0.5011	0.4868
Num. obs.	612	601	582

$^{***}p < 0.001$; $^{**}p < 0.01$; $^{*}p < 0.05$

Table S31: Testing the Effect of the Intervention on Count of Reliable News Referred From Search Engines and Social Media (Google, Twitter, and Facebook) with Covariate-Adjusted Models (HC2 Robust standard errors) (After July 1st)

	Intent-To-Treat (ITT)	CACE (Model 1)	CACE (Model 2)
Treatment	0.0135	0.0164	0.0057
	(0.0306)	(0.0323)	(0.0370)
Age	0.0042***	0.0043***	0.0043***
	(0.0012)	(0.0012)	(0.0012)
Log of news viewed	0.0504***	0.0495***	0.0531***
	(0.0123)	(0.0126)	(0.0129)
Pre-Treatment Value	0.7207***	0.7204***	0.7231***
	(0.0279)	(0.0283)	(0.0291)
R^2	0.6932	0.6908	0.6928
Adj. R^2	0.6912	0.6887	0.6907
Num. obs.	608	597	578

$^{***}p < 0.001$; $^{**}p < 0.01$; $^{*}p < 0.05$

Table S32: Testing the Effect of the Intervention on Reliability Score of News Referred From Search Engines and Social Media (Google, Twitter, and Facebook) with Covariate-Adjusted Models (HC2 Robust standard errors) (After July 1st)

	Intent-To-Treat (ITT)	CACE (Model 1)	CACE (Model 2)
Treatment	0.1765	0.2247	0.2338
	(0.6905)	(0.7352)	(0.8291)
Pre-Treatment Value	0.5535***	0.5430***	0.5196***
	(0.1019)	(0.1026)	(0.1051)
R^2	0.3256	0.3159	0.2925
Adj. R^2	0.3231	0.3133	0.2898
Num. obs.	548	538	522

$^{***}p < 0.001$; $^{**}p < 0.01$; $^{*}p < 0.05$

SC.7 Attitudinal Measures

Table S33: Testing Effect of Intervention on Belief in Misinformation about the Black Lives Matter Movement with Covariate-Adjusted Models (HC2 Robust standard errors)

	Intent-To-Treat (ITT)	CACE (Model 1)	CACE (Model 2)
Treatment	−0.0206	−0.0219	−0.0225
	(0.0297)	(0.0317)	(0.0380)
Web Browser (Safari)	−0.1440***	−0.1481***	−0.1391***
	(0.0409)	(0.0411)	(0.0421)
Party ID	0.0135	0.0142	0.0133
	(0.0118)	(0.0120)	(0.0124)
Education	−0.0291**	−0.0337**	−0.0325**
	(0.0110)	(0.0111)	(0.0113)
Gender	0.0756*	0.0731*	0.0624*
	(0.0301)	(0.0304)	(0.0310)
Age	0.0018	0.0021	0.0015
	(0.0011)	(0.0011)	(0.0012)
Trust in Media	−0.1892***	−0.1786***	−0.1695***
	(0.0229)	(0.0231)	(0.0236)
Ideology	0.1027***	0.1039***	0.1054***
	(0.0122)	(0.0125)	(0.0129)
News consumption (network news)	0.0218*	0.0212*	0.0253*
	(0.0107)	(0.0108)	(0.0110)
News consumption (print news)	0.0543***	0.0507***	0.0472***
	(0.0130)	(0.0133)	(0.0135)
News consumption (talk radio)	0.0601***	0.0580***	0.0616***
	(0.0128)	(0.0129)	(0.0133)
News consumption (news on desktop)	−0.0535***	−0.0535***	−0.0587***
	(0.0128)	(0.0128)	(0.0133)
News consumption (news on mobile)	−0.0282**	−0.0278**	−0.0278*
	(0.0105)	(0.0106)	(0.0108)
Trust of news in newspapers_sm	0.0035***	0.0034***	0.0032***
	(0.0007)	(0.0007)	(0.0007)
Trust of news in newspapers	−0.1067***	−0.1091***	−0.1099***
	(0.0184)	(0.0185)	(0.0189)
R^2	0.2916	0.2926	0.2928
Adj. R^2	0.2875	0.2885	0.2885
Num. obs.	2662	2588	2491

$^{***}p < 0.001$; $^{**}p < 0.01$; $^{*}p < 0.05$

Table S34: Testing Effect of Intervention on Belief in True Information about the Black Lives Matter Movement with Covariate-Adjusted Models (HC2 Robust standard errors)

	Intent-To-Treat (ITT)	CACE (Model 1)	CACE (Model 2)
Treatment	-0.0115	-0.0016	-0.0041
	(0.0265)	(0.0284)	(0.0340)
Party ID	-0.0159	-0.0164	-0.0180
	(0.0098)	(0.0100)	(0.0101)
Trust in Media	0.1577^{***}	0.1600^{***}	0.1629^{***}
	(0.0197)	(0.0200)	(0.0202)
Ideology	-0.0786^{***}	-0.0759^{***}	-0.0745^{***}
	(0.0101)	(0.0103)	(0.0105)
R^2	0.1390	0.1370	0.1421
Adj. R^2	0.1379	0.1359	0.1410
Num. obs.	3193	3104	2982

$^{***}p < 0.001$; $^{**}p < 0.01$; $^{*}p < 0.05$

Table S35: Testing Effect of Intervention on Belief in Misinformation about Covid-19 with Covariate-Adjusted Models (HC2 Robust standard errors)

	Intent-To-Treat (ITT)	CACE (Model 1)	CACE (Model 2)
Treatment	0.0012	0.0034	0.0187
	(0.0267)	(0.0281)	(0.0334)
Party ID	0.0381***	0.0413***	0.0385**
	(0.0114)	(0.0115)	(0.0117)
Race/Ethnicity	−0.1640***	−0.1844***	−0.1947***
	(0.0351)	(0.0354)	(0.0369)
Education	−0.0436***	−0.0472***	−0.0477***
	(0.0096)	(0.0096)	(0.0097)
Trust in Media	−0.2358***	−0.2272***	−0.2158***
	(0.0199)	(0.0198)	(0.0203)
Ideology	0.0695***	0.0728***	0.0754***
	(0.0117)	(0.0118)	(0.0121)
News consumption (print news)	0.0606***	0.0562***	0.0529***
	(0.0111)	(0.0110)	(0.0112)
News consumption (talk radio)	0.0500***	0.0455***	0.0456***
	(0.0116)	(0.0116)	(0.0119)
Trust of news in newspapers_sm	0.0055***	0.0051***	0.0052***
	(0.0006)	(0.0006)	(0.0006)
Trust of news in newspapers	−0.0882***	−0.0826***	−0.0844***
	(0.0161)	(0.0161)	(0.0161)
R^2	0.3182	0.3254	0.3214
Adj. R^2	0.3157	0.3229	0.3188
Num. obs.	2729	2652	2549

$^{***}p < 0.001$; $^{**}p < 0.01$; $^{*}p < 0.05$

Table S36: Testing Effect of Intervention on Belief in True Information about Covid-19 with Covariate-Adjusted Models (HC2 Robust standard errors)

	Intent-To-Treat (ITT)	CACE (Model 1)	CACE (Model 2)
Treatment	0.0004	0.0134	0.0119
	(0.0183)	(0.0195)	(0.0233)
Trust in Media	0.0896^{***}	0.0855^{***}	0.0781^{***}
	(0.0144)	(0.0143)	(0.0145)
Ideology	-0.0259^{***}	-0.0291^{***}	-0.0293^{***}
	(0.0056)	(0.0055)	(0.0056)
News consumption (talk radio)	-0.0428^{***}	-0.0403^{***}	-0.0439^{***}
	(0.0082)	(0.0083)	(0.0084)
Trust of news in newspapers	0.0719^{***}	0.0689^{***}	0.0696^{***}
	(0.0113)	(0.0113)	(0.0115)
R^2	0.1122	0.1129	0.1135
Adj. R^2	0.1108	0.1115	0.1121
Num. obs.	3267	3172	3048

$^{***}p < 0.001$; $^{**}p < 0.01$; $^{*}p < 0.05$

Table S37: Testing Effect of Intervention on Trust in Media with Covariate-Adjusted Models (HC2 Robust standard errors)

	Intent-To-Treat (ITT)	CACE (Model 1)	CACE (Model 2)
Treatment	0.0303	0.0263	0.0373
	(0.0200)	(0.0214)	(0.0258)
Party ID	-0.0550^{***}	-0.0569^{***}	-0.0590^{***}
	(0.0076)	(0.0078)	(0.0079)
Trust in Media	0.5555^{***}	0.5529^{***}	0.5635^{***}
	(0.0154)	(0.0156)	(0.0160)
Ideology	-0.0564^{***}	-0.0537^{***}	-0.0503^{***}
	(0.0075)	(0.0076)	(0.0077)
Trust of news in newspapers	0.0771^{***}	0.0813^{***}	0.0750^{***}
	(0.0115)	(0.0116)	(0.0118)
R^2	0.6003	0.6006	0.6061
Adj. R^2	0.5997	0.5999	0.6054
Num. obs.	3335	3237	3108

$^{***}p < 0.001$; $^{**}p < 0.01$; $^{*}p < 0.05$

Table S38: Testing Effect of Intervention on Affective Polarization with Covariate-Adjusted Models (HC2 Robust standard errors)

	Intent-To-Treat (ITT)	CACE (Model 1)	CACE (Model 2)
Treatment	0.0276	0.0602	0.0811
	(0.5763)	(0.6108)	(0.7286)
Pre-Treatment Value	0.8702***	0.8675***	0.8678***
	(0.0095)	(0.0097)	(0.0098)
R^2	0.7159	0.7173	0.7198
Adj. R^2	0.7157	0.7172	0.7196
Num. obs.	3193	3103	2987

$^{***}p < 0.001$; $^{**}p < 0.01$; $^{*}p < 0.05$

Table S39: Testing Effect of Intervention on Whether They Believe "Fake News is a Problem" with Covariate-Adjusted Models (HC2 Robust standard errors)

	Intent-To-Treat (ITT)	CACE (Model 1)	CACE (Model 2)
Pre-Treatment Value	0.5486^{***}	0.5483^{***}	0.5582^{***}
	(0.0175)	(0.0177)	(0.0181)
Treatment	−0.0093	−0.0122	−0.0118
	(0.0224)	(0.0239)	(0.0289)
Party ID	0.0159	0.0198^{*}	0.0201^{*}
	(0.0084)	(0.0084)	(0.0086)
Trust in Media	-0.1056^{***}	-0.1039^{***}	-0.1055^{***}
	(0.0172)	(0.0175)	(0.0180)
Ideology	0.0317^{***}	0.0292^{***}	0.0271^{**}
	(0.0088)	(0.0088)	(0.0091)
R^2	0.3984	0.4013	0.4109
Adj. R^2	0.3975	0.4004	0.4099
Num. obs.	3335	3237	3108

$^{***}p < 0.001$; $^{**}p < 0.01$; $^{*}p < 0.05$

Table S40: Testing Effect of Intervention on Whether They Believe "Fake News is a Problem in the Mainstream Media" with Covariate-Adjusted Models (HC2 Robust standard errors)

	Intent-To-Treat (ITT)	CACE (Model 1)	CACE (Model 2)
Pre-Treatment Value	0.5867***	0.5917***	0.5935***
	(0.0205)	(0.0209)	(0.0213)
Treatment	−0.0426	−0.0416	−0.0361
	(0.0293)	(0.0313)	(0.0376)
Party ID	0.0635***	0.0608***	0.0618***
	(0.0127)	(0.0130)	(0.0130)
Trust in Media	−0.2554***	−0.2570***	−0.2579***
	(0.0275)	(0.0278)	(0.0283)
Ideology	0.0869***	0.0844***	0.0801***
	(0.0129)	(0.0131)	(0.0134)
Trust of news in newspapers	−0.1062***	−0.1059***	−0.1089***
	(0.0192)	(0.0195)	(0.0199)
R^2	0.7355	0.7362	0.7397
Adj. R^2	0.7350	0.7357	0.7392
Num. obs.	3334	3236	3107

$^{***}p < 0.001$; $^{**}p < 0.01$; $^{*}p < 0.05$

Table S41: Testing Effect of Intervention on Trust in Institutions with Covariate-Adjusted Models (HC2 Robust standard errors)

	Intent-To-Treat (ITT)	CACE (Model 1)	CACE (Model 2)
Treatment	0.2431	0.3362	0.2293
	(0.3756)	(0.3984)	(0.4781)
Pre-Treatment Value	0.7389***	0.7419***	0.7438***
	(0.0133)	(0.0131)	(0.0134)
R^2	0.5870	0.5937	0.5922
Adj. R^2	0.5867	0.5934	0.5919
Num. obs.	3007	2922	2815

$^{***}p < 0.001$; $^{**}p < 0.01$; $^{*}p < 0.05$

Table S42: Testing Effect of Intervention on Trust in CBS with Covariate-Adjusted Models (HC2 Robust standard errors)

	Intent-To-Treat (ITT)	CACE (Model 1)	CACE (Model 2)
Treatment	0.0440	0.0411	0.0440
	(0.0281)	(0.0299)	(0.0358)
Pre-Treatment Value	0.4610^{***}	0.4626^{***}	0.4567^{***}
	(0.0214)	(0.0216)	(0.0220)
Party ID	-0.0417^{***}	-0.0415^{***}	-0.0448^{***}
	(0.0105)	(0.0105)	(0.0107)
Trust in Media	0.1899^{***}	0.1903^{***}	0.1818^{***}
	(0.0215)	(0.0217)	(0.0222)
Ideology	-0.0485^{***}	-0.0471^{***}	-0.0460^{***}
	(0.0111)	(0.0113)	(0.0115)
News consumption (network news)	0.0711^{***}	0.0733^{***}	0.0719^{***}
	(0.0101)	(0.0102)	(0.0104)
Trust of news in newspapers_sm	0.0031^{***}	0.0032^{***}	0.0031^{***}
	(0.0006)	(0.0006)	(0.0007)
Trust of news in newspapers	0.0485^{**}	0.0497^{**}	0.0603^{**}
	(0.0187)	(0.0189)	(0.0193)
R^2	0.5739	0.5767	0.5770
Adj. R^2	0.5726	0.5753	0.5755
Num. obs.	2472	2401	2304

$^{***}p < 0.001$; $^{**}p < 0.01$; $^{*}p < 0.05$

Table S43: Testing Effect of Intervention on Trust in ABC with Covariate-Adjusted Models (HC2 Robust standard errors)

	Intent-To-Treat (ITT)	CACE (Model 1)	CACE (Model 2)
Treatment	0.0303	0.0301	0.0349
	(0.0284)	(0.0300)	(0.0361)
Pre-Treatment Value	0.4644***	0.4719***	0.4748***
	(0.0222)	(0.0223)	(0.0228)
Party ID	−0.0554***	−0.0539***	−0.0585***
	(0.0108)	(0.0108)	(0.0111)
Trust in Media	0.1521***	0.1522***	0.1329***
	(0.0229)	(0.0228)	(0.0230)
Ideology	−0.0526***	−0.0520***	−0.0513***
	(0.0114)	(0.0116)	(0.0119)
News consumption (network news)	0.0636***	0.0653***	0.0646***
	(0.0103)	(0.0104)	(0.0106)
Trust of news in newspapers_sm	0.0033***	0.0033***	0.0033***
	(0.0006)	(0.0006)	(0.0006)
Trust of news in newspapers	0.0347	0.0334	0.0408*
	(0.0194)	(0.0196)	(0.0199)
R^2	0.5648	0.5709	0.5755
Adj. R^2	0.5634	0.5694	0.5740
Num. obs.	2451	2382	2289

$^{***}p < 0.001$; $^{**}p < 0.01$; $^{*}p < 0.05$

Table S44: Testing Effect of Intervention on Trust in NBC with Covariate-Adjusted Models (HC2 Robust standard errors)

	Intent-To-Treat (ITT)	CACE (Model 1)	CACE (Model 2)
Treatment	0.0238	0.0283	0.0296
	(0.0272)	(0.0287)	(0.0346)
Pre-Treatment Value	0.4703***	0.4756***	0.4762***
	(0.0220)	(0.0220)	(0.0223)
Party ID	−0.0536***	−0.0514***	−0.0537***
	(0.0105)	(0.0106)	(0.0106)
Trust in Media	0.1856***	0.1971***	0.1806***
	(0.0226)	(0.0230)	(0.0233)
Ideology	−0.0515***	−0.0499***	−0.0503***
	(0.0112)	(0.0114)	(0.0116)
News consumption (network news)	0.0614***	0.0613***	0.0611***
	(0.0099)	(0.0099)	(0.0101)
Trust of news in newspapers_sm	0.0031***	0.0031***	0.0030***
	(0.0006)	(0.0006)	(0.0006)
Trust of news in newspapers	0.0440*	0.0422*	0.0507**
	(0.0184)	(0.0184)	(0.0188)
R^2	0.6064	0.6137	0.6149
Adj. R^2	0.6051	0.6124	0.6136
Num. obs.	2447	2379	2287

$^{***}p < 0.001$; $^{**}p < 0.01$; $^{*}p < 0.05$

Table S45: Testing Effect of Intervention on Trust in CNN with Covariate-Adjusted Models (HC2 Robust standard errors)

	Intent-To-Treat (ITT)	CACE (Model 1)	CACE (Model 2)
Treatment	0.0149	0.0196	0.0248
	(0.0249)	(0.0266)	(0.0320)
Pre-Treatment Value	0.5751^{***}	0.5744^{***}	0.5639^{***}
	(0.0182)	(0.0183)	(0.0188)
Party ID	-0.0753^{***}	-0.0757^{***}	-0.0782^{***}
	(0.0104)	(0.0106)	(0.0109)
Trust in Media	0.1922^{***}	0.1935^{***}	0.2006^{***}
	(0.0200)	(0.0203)	(0.0209)
Ideology	-0.0375^{***}	-0.0376^{***}	-0.0373^{**}
	(0.0108)	(0.0111)	(0.0113)
R^2	0.6601	0.6618	0.6636
Adj. R^2	0.6595	0.6612	0.6630
Num. obs.	2887	2806	2691

$^{***}p < 0.001$; $^{**}p < 0.01$; $^{*}p < 0.05$

Table S46: Testing Effect of Intervention on Trust in Fox News with Covariate-Adjusted Models (HC2 Robust standard errors)

	Intent-To-Treat (ITT)	CACE (Model 1)	CACE (Model 2)
Treatment	0.0026	−0.0012	−0.0100
	(0.0231)	(0.0243)	(0.0294)
Pre-Treatment Value	0.6468***	0.6427***	0.6449***
	(0.0176)	(0.0179)	(0.0182)
Party ID	0.0263**	0.0277**	0.0254**
	(0.0086)	(0.0086)	(0.0088)
Ideology	0.0734***	0.0752***	0.0758***
	(0.0095)	(0.0097)	(0.0099)
R^2	0.6046	0.6078	0.6084
Adj. R^2	0.6040	0.6073	0.6079
Num. obs.	2883	2805	2692

$^{***}p < 0.001$; $^{**}p < 0.01$; $^{*}p < 0.05$

SD Results From All Covariate-Unadjusted Models

SD.1 Behavioral Measures (Before July 1st)

Table S47: Testing the Effect of the Intervention on Proportion of News Diet That is Unreliable with Covariate-Unadjusted Models (HC2 Robust standard errors) (Before July 1st)

	Intent-To-Treat (ITT)	CACE (Model 1)	CACE (Model 2)
Treatment	0.0005	0.0006	−0.0006
	(0.0071)	(0.0075)	(0.0086)
R^2	0.0000	0.0000	−0.0000
Adj. R^2	−0.0012	−0.0011	−0.0013
Num. obs.	857	841	818

$^{***}p < 0.001$; $^{**}p < 0.01$; $^{*}p < 0.05$

Table S48: Testing the Effect of the Intervention on Proportion of News Diet That is Reliable with Covariate-Unadjusted Models (HC2 Robust standard errors) (Before July 1st)

	Intent-To-Treat (ITT)	CACE (Model 1)	CACE (Model 2)
Treatment	−0.0110	−0.0105	−0.0185
	(0.0215)	(0.0225)	(0.0257)
R^2	0.0003	0.0005	0.0005
Adj. R^2	−0.0009	−0.0007	−0.0007
Num. obs.	857	841	818

$^{***}p < 0.001$; $^{**}p < 0.01$; $^{*}p < 0.05$

Table S49: Testing the Effect of the Intervention on Count of Unreliable News Consumed with Covariate-Unadjusted Models (HC2 Robust standard errors) (Before July 1st)

	Intent-To-Treat (ITT)	CACE (Model 1)	CACE (Model 2)
Treatment	0.0290	0.0338	0.0373
	(0.0395)	(0.0414)	(0.0475)
R^2	0.0006	0.0012	0.0015
Adj. R^2	−0.0005	0.0001	0.0004
Num. obs.	904	885	860

$^{***}p < 0.001$; $^{**}p < 0.01$; $^{*}p < 0.05$

Table S50: Testing the Effect of the Intervention on Count of Reliable News Consumed with Covariate-Unadjusted Models (HC2 Robust standard errors) (Before July 1st)

	Intent-To-Treat (ITT)	CACE (Model 1)	CACE (Model 2)
Treatment	−0.0161	−0.0029	−0.0034
	(0.0597)	(0.0623)	(0.0717)
R^2	0.0001	−0.0001	−0.0001
Adj. R^2	−0.0010	−0.0012	−0.0012
Num. obs.	904	885	860

$^{***}p < 0.001$; $^{**}p < 0.01$; $^{*}p < 0.05$

Table S51: Testing the Effect of the Intervention on Reliability Score of News Diet with Covariate-Unadjusted Models (HC2 Robust standard errors) (Before July 1st)

	Intent-To-Treat (ITT)	CACE (Model 1)	CACE (Model 2)
Treatment	0.0549	0.1503	0.1080
	(0.5771)	(0.6023)	(0.6867)
R^2	0.0000	0.0000	−0.0001
Adj. R^2	−0.0012	−0.0012	−0.0013
Num. obs.	857	841	818

$^{***}p < 0.001$; $^{**}p < 0.01$; $^{*}p < 0.05$

SD.2 Behavioral Measures (After July 1st)

Table S52: Testing the Effect of the Intervention on Proportion of News Diet That is Unreliable with Covariate-Unadjusted Models (HC2 Robust standard errors) (After July 1st)

	Intent-To-Treat (ITT)	CACE (Model 1)	CACE (Model 2)
Treatment	−0.0016	−0.0013	−0.0010
	(0.0074)	(0.0078)	(0.0091)
R^2	0.0001	−0.0000	0.0002
Adj. R^2	−0.0012	−0.0013	−0.0010
Num. obs.	813	800	774

$^{***}p < 0.001$; $^{**}p < 0.01$; $^{*}p < 0.05$

Table S53: Testing the Effect of the Intervention on Proportion of News Diet That is Reliable with Covariate-Unadjusted Models (HC2 Robust standard errors) (After July 1st)

	Intent-To-Treat (ITT)	CACE (Model 1)	CACE (Model 2)
Treatment	0.0111	0.0129	0.0094
	(0.0223)	(0.0233)	(0.0269)
R^2	0.0003	−0.0001	0.0002
Adj. R^2	−0.0009	−0.0013	−0.0011
Num. obs.	813	800	774

$^{***}p < 0.001$; $^{**}p < 0.01$; $^{*}p < 0.05$

Table S54: Testing the Effect of the Intervention on Count of Unreliable News Consumed with Covariate-Unadjusted Models (HC2 Robust standard errors) (After July 1st)

	Intent-To-Treat (ITT)	CACE (Model 1)	CACE (Model 2)
Treatment	0.0090	0.0114	0.0200
	(0.0326)	(0.0342)	(0.0395)
R^2	0.0001	0.0003	0.0000
Adj. R^2	−0.0010	−0.0008	−0.0012
Num. obs.	884	867	839

$^{***}p < 0.001$; $^{**}p < 0.01$; $^{*}p < 0.05$

Table S55: Testing the Effect of the Intervention on Count of Reliable News Consumed with Covariate-Unadjusted Models (HC2 Robust standard errors) (After July 1st)

	Intent-To-Treat (ITT)	CACE (Model 1)	CACE (Model 2)
Treatment	−0.0403	−0.0270	−0.0368
	(0.0578)	(0.0602)	(0.0701)
R^2	0.0005	−0.0002	−0.0011
Adj. R^2	−0.0006	−0.0013	−0.0023
Num. obs.	884	867	839

$^{***}p < 0.001$; $^{**}p < 0.01$; $^{*}p < 0.05$

Table S56: Testing the Effect of the Intervention on Reliability Score of News Diet with Covariate-Unadjusted Models (HC2 Robust standard errors) (After July 1st)

	Intent-To-Treat (ITT)	CACE (Model 1)	CACE (Model 2)
Treatment	0.1774	0.2296	0.1439
	(0.5990)	(0.6268)	(0.7222)
R^2	0.0001	0.0000	0.0004
Adj. R^2	−0.0011	−0.0012	−0.0009
Num. obs.	813	800	774

$^{***}p < 0.001$; $^{**}p < 0.01$; $^{*}p < 0.05$

SD.3 Behavioral Measures - Weighted by Duration (Before July 1st)

Table S57: Testing the Effect of the Intervention on Proportion of News Diet That is Unreliable with Covariate-Adjusted Models (HC2 Robust standard errors) (Before July 1st) – Duration Weighted

	Intent-To-Treat (ITT)	CACE (Model 1)	CACE (Model 2)
Treated	−0.0036	−0.0034	−0.0053
	(0.0075)	(0.0079)	(0.0090)
R^2	0.0003	−0.0001	−0.0003
Adj. R^2	−0.0009	−0.0013	−0.0015
Num. obs.	856	840	817

$^{***}p < 0.001$; $^{**}p < 0.01$; $^{*}p < 0.05$

Table S58: Testing the Effect of the Intervention on Proportion of News Diet That is Reliable with Covariate-Adjusted Models (HC2 Robust standard errors) (Before July 1st) – Duration Weighted

	Intent-To-Treat (ITT)	CACE (Model 1)	CACE (Model 2)
Treated	−0.0035	−0.0023	−0.0126
	(0.0232)	(0.0242)	(0.0276)
R^2	0.0000	0.0000	−0.0000
Adj. R^2	−0.0011	−0.0012	−0.0012
Num. obs.	856	840	817

$^{***}p < 0.001$; $^{**}p < 0.01$; $^{*}p < 0.05$

Table S59: Testing the Effect of the Intervention on Count of Unreliable News Consumed with Covariate-Adjusted Models (HC2 Robust standard errors) (Before July 1st) – Duration Weighted

	Intent-To-Treat (ITT)	CACE (Model 1)	CACE (Model 2)
Treated	−457.2273	−475.0275	−554.2217
	(350.1165)	(370.3970)	(433.1678)
R^2	0.0028	0.0020	−0.0004
Adj. R^2	0.0017	0.0008	−0.0016
Num. obs.	904	885	860

$^{***}p < 0.001$; $^{**}p < 0.01$; $^{*}p < 0.05$

Table S60: Testing the Effect of the Intervention on Count of Reliable News Consumed with Covariate-Adjusted Models (HC2 Robust standard errors) (Before July 1st) – Duration Weighted

	Intent-To-Treat (ITT)	CACE (Model 1)	CACE (Model 2)
Treated	1578.2085	1734.1659	2373.7616
	(4285.7158)	(4539.8499)	(5303.0171)
R^2	0.0001	0.0003	0.0008
Adj. R^2	−0.0010	−0.0008	−0.0004
Num. obs.	904	885	860

$^{***}p < 0.001$; $^{**}p < 0.01$; $^{*}p < 0.05$

Table S61: Testing the Effect of the Intervention on Reliability Score of News Diet with Covariate-Adjusted Models (HC2 Robust standard errors) (Before July 1st) – Duration Weighted

	Intent-To-Treat (ITT)	CACE (Model 1)	CACE (Model 2)
Treated	0.4935	0.5961	0.5397
	(0.6256)	(0.6540)	(0.7463)
R^2	0.0007	0.0011	0.0006
Adj. R^2	−0.0004	−0.0001	−0.0006
Num. obs.	856	840	817

$^{***}p < 0.001$; $^{**}p < 0.01$; $^{*}p < 0.05$

SD.4 Behavioral Measures - Weighted by Duration (After July 1st)

Table S62: Testing the Effect of the Intervention on Proportion of News Diet That is Unreliable with Covariate-Adjusted Models (HC2 Robust standard errors) (After July 1st) – Duration Weighted

	Intent-To-Treat (ITT)	CACE (Model 1)	CACE (Model 2)
Treated	−0.0049	−0.0050	−0.0055
	(0.0083)	(0.0087)	(0.0102)
R^2	0.0005	0.0000	0.0012
Adj. R^2	−0.0008	−0.0012	−0.0001
Num. obs.	811	798	772

$^{***}p < 0.001$; $^{**}p < 0.01$; $^{*}p < 0.05$

Table S63: Testing the Effect of the Intervention on Proportion of News Diet That is Reliable with Covariate-Adjusted Models (HC2 Robust standard errors) (After July 1st) – Duration Weighted

	Intent-To-Treat (ITT)	CACE (Model 1)	CACE (Model 2)
Treated	0.0138	0.0176	0.0137
	(0.0240)	(0.0250)	(0.0288)
R^2	0.0004	0.0003	0.0006
Adj. R^2	−0.0008	−0.0010	−0.0007
Num. obs.	811	798	772

$^{***}p < 0.001$; $^{**}p < 0.01$; $^{*}p < 0.05$

Table S64: Testing the Effect of the Intervention on Count of Unreliable News Consumed with Covariate-Adjusted Models (HC2 Robust standard errors) (After July 1st) – Duration Weighted

	Intent-To-Treat (ITT)	CACE (Model 1)	CACE (Model 2)
Treated	−123.5642	−124.8044	−140.8097
	(162.4097)	(171.1623)	(201.6918)
R^2	0.0010	0.0005	−0.0004
Adj. R^2	−0.0002	−0.0006	−0.0016
Num. obs.	884	867	839

$^{***}p < 0.001$; $^{**}p < 0.01$; $^{*}p < 0.05$

Table S65: Testing the Effect of the Intervention on Count of Reliable News Consumed with Covariate-Adjusted Models (HC2 Robust standard errors) (After July 1st) – Duration Weighted

	Intent-To-Treat (ITT)	CACE (Model 1)	CACE (Model 2)
Treated	1827.2807	2006.9349	2438.3461
	(1762.6270)	(1860.7421)	(2188.5690)
R^2	0.0010	0.0015	0.0031
Adj. R^2	−0.0001	0.0004	0.0019
Num. obs.	884	867	839

$^{***}p < 0.001$; $^{**}p < 0.01$; $^{*}p < 0.05$

Table S66: Testing the Effect of the Intervention on Reliability Score of News Diet with Covariate-Adjusted Models (HC2 Robust standard errors) (After July 1st) – Duration Weighted

	Intent-To-Treat (ITT)	CACE (Model 1)	CACE (Model 2)
Treated	0.5698	0.6748	0.6405
	(0.6663)	(0.6985)	(0.8020)
R^2	0.0009	0.0009	0.0024
Adj. R^2	-0.0003	-0.0004	0.0011
Num. obs.	811	798	772

$^{***}p < 0.001$; $^{**}p < 0.01$; $^{*}p < 0.05$

SD.5 Behavioral Measures - Referrals from Social Media Sites and Search Engines (Before July 1st)

Table S67: Testing the Effect of the Intervention on Proportion of News Diet of Referrals From Search Engines and Social Media (Google, Twitter, and Facebook) That is Unreliable with Covariate-Adjusted Models (HC2 Robust standard errors) (Before July 1st)

	Intent-To-Treat (ITT)	CACE (Model 1)	CACE (Model 2)
Treated	−0.0012	−0.0018	−0.0032
	(0.0082)	(0.0087)	(0.0098)
R^2	0.0000	0.0000	−0.0002
Adj. R^2	−0.0013	−0.0014	−0.0017
Num. obs.	733	721	700

$^{***}p < 0.001$; $^{**}p < 0.01$; $^{*}p < 0.05$

Table S68: Testing the Effect of the Intervention on Proportion of News Diet of Referrals From Search Engines and Social Media (Google, Twitter, and Facebook) That is Reliable with Covariate-Adjusted Models (HC2 Robust standard errors) (Before July 1st)

	Intent-To-Treat (ITT)	CACE (Model 1)	CACE (Model 2)
Treated	−0.0284	−0.0273	−0.0364
	(0.0216)	(0.0225)	(0.0257)
R^2	0.0023	0.0019	0.0018
Adj. R^2	0.0010	0.0005	0.0003
Num. obs.	733	721	700

$^{***}p < 0.001$; $^{**}p < 0.01$; $^{*}p < 0.05$

Table S69: Testing the Effect of the Intervention on Count of Unreliable News Referred From Search Engines and Social Media (Google, Twitter, and Facebook) with Covariate-Adjusted Models (HC2 Robust standard errors) (Before July 1st)

	Intent-To-Treat (ITT)	CACE (Model 1)	CACE (Model 2)
Treated	−0.0117	−0.0139	−0.0147
	(0.0252)	(0.0262)	(0.0298)
R^2	0.0003	0.0001	−0.0003
Adj. R^2	−0.0010	−0.0012	−0.0017
Num. obs.	770	757	735

$^{***}p < 0.001$; $^{**}p < 0.01$; $^{*}p < 0.05$

Table S70: Testing the Effect of the Intervention on Count of Reliable News Referred From Search Engines and Social Media (Google, Twitter, and Facebook) with Covariate-Adjusted Models (HC2 Robust standard errors) (Before July 1st)

	Intent-To-Treat (ITT)	CACE (Model 1)	CACE (Model 2)
Treated	−0.0481	−0.0447	−0.0604
	(0.0497)	(0.0516)	(0.0595)
R^2	0.0012	0.0010	0.0029
Adj. R^2	−0.0001	−0.0003	0.0015
Num. obs.	770	757	735

$^{***}p < 0.001$; $^{**}p < 0.01$; $^{*}p < 0.05$

Table S71: Testing the Effect of the Intervention on Reliability Score of News Referred From Search Engines and Social Media (Google, Twitter, and Facebook) with Covariate-Adjusted Models (HC2 Robust standard errors) (Before July 1st)

	Intent-To-Treat (ITT)	CACE (Model 1)	CACE (Model 2)
Treated	0.3393	0.4237	0.2932
	(0.7678)	(0.8001)	(0.9142)
R^2	0.0003	0.0009	0.0000
Adj. R^2	−0.0011	−0.0005	−0.0014
Num. obs.	733	721	700

$^{***}p < 0.001$; $^{**}p < 0.01$; $^{*}p < 0.05$

SD.6 Behavioral Measures - Referrals from Social Media Sites and Search Engines (After July 1st)

Table S72: Testing the Effect of the Intervention on Proportion of News Diet of Referrals From Search Engines and Social Media (Google, Twitter, and Facebook) That is Unreliable with Covariate-Adjusted Models (HC2 Robust standard errors) (After July 1st)

	Intent-To-Treat (ITT)	CACE (Model 1)	CACE (Model 2)
Treated	0.0088	0.0087	0.0093
	(0.0081)	(0.0085)	(0.0098)
R^2	0.0016	0.0023	0.0006
Adj. R^2	0.0000	0.0007	−0.0011
Num. obs.	630	620	598

$^{***}p < 0.001$; $^{**}p < 0.01$; $^{*}p < 0.05$

Table S73: Testing the Effect of the Intervention on Proportion News Diet of Referrals From Search Engines and Social Media (Google, Twitter, and Facebook) That is Reliable with Covariate-Adjusted Models (HC2 Robust standard errors) (After July 1st)

	Intent-To-Treat (ITT)	CACE (Model 1)	CACE (Model 2)
Treated	0.0117	0.0156	0.0108
	(0.0234)	(0.0243)	(0.0279)
R^2	0.0004	0.0002	0.0007
Adj. R^2	−0.0012	−0.0014	−0.0009
Num. obs.	630	620	598

$^{***}p < 0.001$; $^{**}p < 0.01$; $^{*}p < 0.05$

Table S74: Testing the Effect of the Intervention on Count of Unreliable News Referred From Search Engines and Social Media (Google, Twitter, and Facebook) with Covariate-Adjusted Models (HC2 Robust standard errors) (After July 1st)

	Intent-To-Treat (ITT)	CACE (Model 1)	CACE (Model 2)
Treated	0.0147	0.0118	0.0175
	(0.0210)	(0.0220)	(0.0251)
R^2	0.0008	0.0010	0.0006
Adj. R^2	−0.0007	−0.0005	−0.0009
Num. obs.	680	669	647

$^{***}p < 0.001$; $^{**}p < 0.01$; $^{*}p < 0.05$

Table S75: Testing the Effect of the Intervention on Count of Reliable News Referred From Search Engines and Social Media (Google, Twitter, and Facebook) with Covariate-Adjusted Models (HC2 Robust standard errors) (After July 1st)

	Intent-To-Treat (ITT)	CACE (Model 1)	CACE (Model 2)
Treated	−0.0462	−0.0410	−0.0679
	(0.0501)	(0.0520)	(0.0601)
R^2	0.0013	0.0016	0.0027
Adj. R^2	−0.0002	0.0001	0.0011
Num. obs.	680	669	647

$^{***}p < 0.001$; $^{**}p < 0.01$; $^{*}p < 0.05$

Table S76: Testing the Effect of the Intervention on Reliability Score of News Referred From Search Engines and Social Media (Google, Twitter, and Facebook) with Covariate-Adjusted Models (HC2 Robust standard errors) (After July 1st)

	Intent-To-Treat (ITT)	CACE (Model 1)	CACE (Model 2)
Treated	−0.5662	−0.4744	−0.6503
	(0.8032)	(0.8397)	(0.9523)
R^2	0.0007	0.0011	−0.0002
Adj. R^2	−0.0008	−0.0005	−0.0018
Num. obs.	630	620	598

$^{***}p < 0.001$; $^{**}p < 0.01$; $^{*}p < 0.05$

SD.7 Attitudinal Measures

Table S77: Testing Effect of Intervention on Belief in Misinformation about the Black Lives Matter Movement with Covariate-Unadjusted Models (HC2 Robust standard errors)

	Intent-To-Treat (ITT)	CACE (Model 1)	CACE (Model 2)
Treatment	-0.0728^{*}	-0.0764^{*}	-0.0939^{*}
	(0.0323)	(0.0344)	(0.0414)
R^2	0.0016	0.0032	0.0063
Adj. R^2	0.0013	0.0029	0.0060
Num. obs.	3170	3079	2962

$^{***}p < 0.001$; $^{**}p < 0.01$; $^{*}p < 0.05$

Table S78: Testing Effect of Intervention on Belief in True Information about the Black Lives Matter Movement with Covariate-Unadjusted Models (HC2 Robust standard errors)

	Intent-To-Treat (ITT)	CACE (Model 1)	CACE (Model 2)
Treatment	0.0004	0.0110	0.0162
	(0.0285)	(0.0305)	(0.0366)
R^2	0.0000	−0.0000	0.0002
Adj. R^2	−0.0003	−0.0003	−0.0001
Num. obs.	3195	3105	2983

$^{***}p < 0.001$; $^{**}p < 0.01$; $^{*}p < 0.05$

Table S79: Testing Effect of Intervention on Belief in Misinformation about Covid-19 with Covariate-Unadjusted Models (HC2 Robust standard errors)

	Intent-To-Treat (ITT)	CACE (Model 1)	CACE (Model 2)
Treatment	−0.0170	−0.0125	−0.0059
	(0.0288)	(0.0304)	(0.0363)
R^2	0.0001	0.0005	0.0005
Adj. R^2	−0.0002	0.0002	0.0001
Num. obs.	3263	3169	3044

$^{***}p < 0.001$; $^{**}p < 0.01$; $^{*}p < 0.05$

Table S80: Testing Effect of Intervention on Belief in True Information about Covid-19 with Covariate-Unadjusted Models (HC2 Robust standard errors)

	Intent-To-Treat (ITT)	CACE (Model 1)	CACE (Model 2)
Treatment	0.0076	0.0202	0.0218
	(0.0196)	(0.0209)	(0.0249)
R^2	0.0000	0.0007	0.0016
Adj. R^2	−0.0003	0.0004	0.0013
Num. obs.	3270	3174	3050

$^{***}p < 0.001$; $^{**}p < 0.01$; $^{*}p < 0.05$

Table S81: Testing Effect of Intervention on Trust in Media with Covariate-Unadjusted Models (HC2 Robust standard errors)

	Intent-To-Treat (ITT)	CACE (Model 1)	CACE (Model 2)
Treatment	0.0478	0.0413	0.0627
	(0.0316)	(0.0338)	(0.0410)
R^2	0.0007	0.0011	0.0022
Adj. R^2	0.0004	0.0008	0.0019
Num. obs.	3337	3238	3109

$^{***}p < 0.001$; $^{**}p < 0.01$; $^{*}p < 0.05$

Table S82: Testing Effect of Intervention on Affective Polarization with Covariate-Unadjusted Models (HC2 Robust standard errors)

	Intent-To-Treat (ITT)	CACE (Model 1)	CACE (Model 2)
Treatment	−0.3371	−0.3549	−0.8156
	(1.0770)	(1.1467)	(1.3733)
R^2	0.0000	0.0000	0.0002
Adj. R^2	−0.0003	−0.0003	−0.0001
Num. obs.	3237	3143	3021

$^{***}p < 0.001$; $^{**}p < 0.01$; $^{*}p < 0.05$

Table S83: Testing Effect of Intervention on Whether They Believe "Fake News is a Problem" with Covariate-Unadjusted Models (HC2 Robust standard errors)

	Intent-To-Treat (ITT)	CACE (Model 1)	CACE (Model 2)
Treatment	−0.0107	−0.0143	−0.0143
	(0.0288)	(0.0309)	(0.0375)
R^2	0.0000	0.0002	0.0002
Adj. R^2	−0.0003	−0.0001	−0.0002
Num. obs.	3337	3238	3109

$^{***}p < 0.001$; $^{**}p < 0.01$; $^{*}p < 0.05$

Table S84: Testing Effect of Intervention on Whether They Believe "Fake News is a Problem in the Mainstream Media" with Covariate-Unadjusted Models (HC2 Robust standard errors)

	Intent-To-Treat (ITT)	CACE (Model 1)	CACE (Model 2)
Treatment	−0.0455	−0.0449	−0.0564
	(0.0580)	(0.0623)	(0.0752)
R^2	0.0002	0.0005	0.0011
Adj. R^2	−0.0001	0.0002	0.0008
Num. obs.	3336	3237	3108

$^{***}p < 0.001$; $^{**}p < 0.01$; $^{*}p < 0.05$

Table S85: Testing Effect of Intervention on Trust in Institutions with Covariate-Unadjusted Models (HC2 Robust standard errors)

	Intent-To-Treat (ITT)	CACE (Model 1)	CACE (Model 2)
Treatment	-0.6346	-0.7537	-1.0348
	(0.5630)	(0.6021)	(0.7237)
R^2	0.0004	0.0010	0.0012
Adj. R^2	0.0001	0.0007	0.0009
Num. obs.	3134	3042	2928

$^{***}p < 0.001$; $^{**}p < 0.01$; $^{*}p < 0.05$

Table S86: Testing Effect of Intervention on Trust in CBS with Covariate-Unadjusted Models (HC2 Robust standard errors)

	Intent-To-Treat (ITT)	CACE (Model 1)	CACE (Model 2)
Treatment	0.0634	0.0663	0.0761
	(0.0371)	(0.0398)	(0.0480)
R^2	0.0009	0.0011	0.0017
Adj. R^2	0.0006	0.0007	0.0014
Num. obs.	3337	3238	3109

$^{***}p < 0.001$; $^{**}p < 0.01$; $^{*}p < 0.05$

Table S87: Testing Effect of Intervention on Trust in ABC with Covariate-Unadjusted Models (HC2 Robust standard errors)

	Intent-To-Treat (ITT)	CACE (Model 1)	CACE (Model 2)
Treatment	0.0569	0.0652	0.0825
	(0.0371)	(0.0397)	(0.0480)
R^2	0.0007	0.0007	0.0015
Adj. R^2	0.0004	0.0004	0.0012
Num. obs.	3337	3238	3109

$^{***}p < 0.001$; $^{**}p < 0.01$; $^{*}p < 0.05$

Table S88: Testing Effect of Intervention on Trust in NBC with Covariate-Unadjusted Models (HC2 Robust standard errors)

	Intent-To-Treat (ITT)	CACE (Model 1)	CACE (Model 2)
Treatment	0.0542	0.0588	0.0738
	(0.0378)	(0.0405)	(0.0489)
R^2	0.0006	0.0009	0.0019
Adj. R^2	0.0003	0.0006	0.0015
Num. obs.	3337	3238	3109

$^{***}p < 0.001$; $^{**}p < 0.01$; $^{*}p < 0.05$

Table S89: Testing Effect of Intervention on Trust in CNN with Covariate-Unadjusted Models (HC2 Robust standard errors)

	Intent-To-Treat (ITT)	CACE (Model 1)	CACE (Model 2)
Treatment	0.0338	0.0357	0.0462
	(0.0410)	(0.0440)	(0.0532)
R^2	0.0002	0.0003	0.0010
Adj. R^2	-0.0001	0.0000	0.0007
Num. obs.	3336	3237	3108

$^{***}p < 0.001$; $^{**}p < 0.01$; $^{*}p < 0.05$

Table S90: Testing Effect of Intervention on Trust in Fox News with Covariate-Unadjusted Models (HC2 Robust standard errors)

	Intent-To-Treat (ITT)	CACE (Model 1)	CACE (Model 2)
Treatment	-0.0108	-0.0162	-0.0285
	(0.0388)	(0.0415)	(0.0501)
R^2	0.0000	0.0005	0.0011
Adj. R^2	-0.0003	0.0001	0.0008
Num. obs.	3336	3237	3108

$^{***}p < 0.001$; $^{**}p < 0.01$; $^{*}p < 0.05$

SE Results From Covariate-Adjusted Model Testing Hypotheses About Moderators

Table S91: Testing Effect of Intervention Initial level of Affective Polarization Moderator on Effect on Affective Polarization (Covariate-Adjusted)

	Affective Polarization
Treatment	−0.6305
	(1.4245)
Moderator	0.8388***
	(0.0159)
Party ID	−0.2634
	(0.1764)
Race/Ethnicity	1.1935
	(0.7890)
Gender	0.2779
	(0.5622)
Age	0.1347***
	(0.0213)
Trust in Media	−0.4657
	(0.4451)
News consumption (cable news)	0.3519
	(0.1871)
News consumption (print news)	−0.6449**
	(0.2290)
News consumption (talk radio)	0.0024
	(0.2337)
News consumption (news on desktop)	0.5342*
	(0.2249)
Treatment*Moderator	0.0130
	(0.0193)
R^2	0.7224
Adj. R^2	0.7214
Num. obs.	3192

***$p < 0.001$; **$p < 0.01$; *$p < 0.05$

Table S92: Testing Effect of Intervention Initial level of Trust In Media (Inverse) Moderator on Effect on Trust In Media (Covariate-Adjusted)

	Trust in Media
Treatment	0.0104
	(0.0356)
Moderator	-0.5641^{***}
	(0.0206)
Party ID	-0.0550^{***}
	(0.0076)
Ideology	-0.0564^{***}
	(0.0075)
Trust of news in newspapers	0.0768^{***}
	(0.0115)
Treatment*Moderator	0.0145
	(0.0223)
R^2	0.6003
Adj. R^2	0.5996
Num. obs.	3335

$^{***}p < 0.001$; $^{**}p < 0.01$; $^{*}p < 0.05$

SF Results From Covariate-Unadjusted Model Testing Hypotheses Regarding Moderators

Table S93: Testing Effect of Intervention Initial level of Affective Polarization Moderator on Effect on Affective Polarization (Covariate-Adjusted)

	Affective Polarization
Treatment	-0.7565
	(1.4484)
Moderator	0.8626^{***}
	(0.0153)
Treatment*Moderator	0.0130
	(0.0196)
R^2	0.7159
Adj. R^2	0.7156
Num. obs.	3193

$^{***}p < 0.001$; $^{**}p < 0.01$; $^{*}p < 0.05$

Table S94: Testing Effect of Intervention Initial level of Trust In Media (Inverse) Moderator on Effect on Trust In Media (Covariate-Adjusted)

	Trust in Media
Treatment	0.0100
	(0.0366)
Moderator	-0.7614^{***}
	(0.0175)
Treatment*Moderator	0.0237
	(0.0228)
R^2	0.5481
Adj. R^2	0.5477
Num. obs.	3337

$^{***}p < 0.001$; $^{**}p < 0.01$; $^{*}p < 0.05$

SG NewsGuard Indicators of Source Reliability

Figure S4: Different NewsGuard Indicators of Source Quality

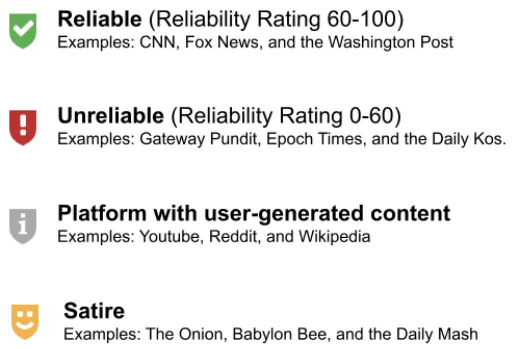

SH Minimum Detectable Effects for Covariate-adjusted ITT and CACE Models

Table S95: Minimum Detectable Effect of Attitudinal Measures assuming power=0.80 and 95 percent Statistical Significance in both the covariate-adjusted Intent-To-Treat and CACE model using the strongest measure

Variable	MDE (ITT)	MDE (CACE)
Affective Polarization	0.037	0.003
Whether They Believe "Fake News is a Problem"	0.053	0.140
Whether They Believe "Fake News is a Problem in the Mainstream Media"	0.035	0.052
Trust in Institutions	0.046	0.007
Trust in CBS	0.052	0.115
Trust in ABC	0.052	0.115
Trust in NBC	0.050	0.109
Trust in CNN	0.043	0.091
Trust in Fox News	0.046	0.111
Belief in Misinformation about the Black Lives Matter Movement	0.065	0.150
Belief in True Information about the Black Lives Matter Movement	0.065	0.160
Belief in Misinformation about Covid-19	0.062	0.163
Belief in True Information about Covid-19	0.065	0.233
Trust in Media	0.043	0.113

Table S96: Minimum Detectable Effect of Behavioral Measures assuming power=0.80 and 95 percent Statistical Significance in both the covariate-adjusted Intent-To-Treat and CACE model using the strongest measure

Variable	MDE (ITT)	MDE (CACE)
Proportion of News Diet That is Unreliable	0.060	1.349
Proportion of News Diet That is Reliable	0.080	0.576
Count of Unreliable News Consumed	0.072	0.272
Count of Reliable News Consumed	0.050	0.145
Avg. Reliability Score of News Diet	0.080	0.021
Proportion of News Diet That is Unreliable	0.070	1.534
Proportion of News Diet That is Reliable	0.089	0.630
Count of Unreliable News Consumed	0.081	0.379
Count of Reliable News Consumed	0.073	0.204
Avg. Reliability Score of News Diet	0.088	0.024

SI Effect of Intervention on Behavioral Measures Using Different Samples of Respondents

Table S97: Testing the Effect of the Intervention on Proportion of News Diet That is Unreliable with Covariate-Adjusted Models (HC2 Robust standard errors) (Before July 1st) on Different Samples of Respondents by Decile of their Quality of News Consumption Pre-Treatment (Average Reliability Score of News)

Decile	Threshold	ITT Model	CACE Model (Weak Compliance Measure)	CACE Model (Strong Compliance Measure)	Sample Size
Bottom 10 Percent	78.255	-0.021 (0.023)	-0.025 (0.025)	-0.03 (0.029)	78
Bottom 20 Percent	85.347	-0.011 (0.013)	-0.013 (0.014)	-0.015 (0.016)	157
Bottom 30 Percent	87.482	-0.007 (0.009)	-0.008 (0.009)	-0.009 (0.01)	237
Bottom 40 Percent	88.383	-0.006 (0.007)	-0.006 (0.007)	-0.007 (0.008)	315
Bottom 50 Percent	89.167	-0.004 (0.005)	-0.005 (0.005)	-0.005 (0.006)	396
Bottom 60 Percent	90	-0.003 (0.004)	-0.004 (0.004)	-0.004 (0.005)	493
Bottom 70 Percent	90.773	-0.003 (0.004)	-0.004 (0.004)	-0.004 (0.005)	549
Bottom 80 Percent	92.462	-0.003 (0.003)	-0.003 (0.003)	-0.004 (0.004)	629
Bottom 90 Percent	95	-0.003 (0.003)	-0.003 (0.003)	-0.004 (0.004)	712

$^{***}p < 0.001$; $^{**}p < 0.01$; $^{*}p < 0.05$

Table S98: Testing the Effect of the Intervention on Proportion of News Diet That is Reliable with Covariate-Adjusted Models (HC2 Robust standard errors) (Before July 1st) on Different Samples of Respondents by Decile of their Quality of News Consumption Pre-Treatment (Average Reliability Score of News)

Decile	Threshold	ITT Model	CACE Model (Weak Compliance Measure)	CACE Model (Strong Compliance Measure)	Sample Size
Bottom 10 Percent	78.255	-0.007 (0.03)	-0.004 (0.032)	-0.007 (0.037)	78
Bottom 20 Percent	85.347	0 (0.029)	0.004 (0.031)	0.003 (0.035)	157
Bottom 30 Percent	87.482	0.01 (0.022)	0.011 (0.023)	0.009 (0.026)	237
Bottom 40 Percent	88.383	0.029 (0.019)	0.032 (0.02)	0.029 (0.023)	315
Bottom 50 Percent	89.167	0.034 (0.017)*	0.036 (0.018)*	0.035 (0.02)	396
Bottom 60 Percent	90	0.013 (0.016)	0.015 (0.016)	0.015 (0.018)	493
Bottom 70 Percent	90.773	0.003 (0.015)	0.005 (0.016)	0.004 (0.018)	549
Bottom 80 Percent	92.462	0.001 (0.013)	0.003 (0.014)	0.001 (0.016)	629
Bottom 90 Percent	95	-0.008 (0.013)	-0.007 (0.013)	-0.008 (0.015)	712

$^{***}p < 0.001$; $^{**}p < 0.01$; $^{*}p < 0.05$

Table S99: Testing the Effect of the Intervention on Count of Unreliable News Consumed with Covariate-Adjusted Models (HC2 Robust standard errors) (Before July 1st) on Different Samples of Respondents by Decile of their Quality of News Consumption Pre-Treatment (Average Reliability Score of News)

Decile	Threshold	ITT Model	CACE Model (Weak Compliance Measure)	CACE Model (Strong Compliance Measure)	Sample Size
Bottom 10 Percent	78.255	0.029 (0.086)	0.02 (0.092)	0.029 (0.107)	79
Bottom 20 Percent	85.347	0.083 (0.068)	0.082 (0.072)	0.1 (0.081)	159
Bottom 30 Percent	87.482	0.076 (0.048)	0.076 (0.051)	0.088 (0.056)	239
Bottom 40 Percent	88.383	0.07 (0.041)	0.073 (0.043)	0.08 (0.049)	317
Bottom 50 Percent	89.167	0.04 (0.035)	0.041 (0.036)	0.044 (0.041)	398
Bottom 60 Percent	90	0.044 (0.03)	0.049 (0.032)	0.054 (0.036)	495
Bottom 70 Percent	90.773	0.037 (0.028)	0.041 (0.03)	0.045 (0.034)	551
Bottom 80 Percent	92.462	0.033 (0.026)	0.037 (0.027)	0.041 (0.031)	631
Bottom 90 Percent	95	0.035 (0.024)	0.039 (0.025)	0.044 (0.029)	714

$^{***}p < 0.001$; $^{**}p < 0.01$; $^{*}p < 0.05$

Table S100: Testing the Effect of the Intervention on Count of Reliable News Consumed with Covariate-Adjusted Models (HC2 Robust standard errors) (Before July 1st) on Different Samples of Respondents by Decile of their Quality of News Consumption Pre-Treatment (Average Reliability Score of News)

Decile	Threshold	ITT Model	CACE Model (Weak Compliance Measure)	CACE Model (Strong Compliance Measure)	Sample Size
Bottom 10 Percent	78.255	0.038 (0.051)	0.044 (0.056)	0.049 (0.065)	79
Bottom 20 Percent	85.347	0.02 (0.038)	0.023 (0.041)	0.024 (0.046)	159
Bottom 30 Percent	87.482	0.001 (0.034)	0 (0.036)	-0.009 (0.039)	239
Bottom 40 Percent	88.383	0.022 (0.029)	0.022 (0.03)	0.016 (0.034)	317
Bottom 50 Percent	89.167	0.029 (0.025)	0.03 (0.026)	0.025 (0.029)	398
Bottom 60 Percent	90	0.017 (0.023)	0.019 (0.024)	0.022 (0.027)	495
Bottom 70 Percent	90.773	0.009 (0.021)	0.01 (0.022)	0.011 (0.025)	551
Bottom 80 Percent	92.462	0.009 (0.02)	0.01 (0.021)	0.01 (0.023)	631
Bottom 90 Percent	95	-0.002 (0.019)	-0.002 (0.02)	-0.002 (0.022)	714

$^{***}p < 0.001$; $^{**}p < 0.01$; $^{*}p < 0.05$

Table S101: Testing the Effect of the Intervention on Reliability Score of News Diet with Covariate-Adjusted Models (HC2 Robust standard errors) (Before July 1st) on Different Samples of Respondents by Decile of their Quality of News Consumption Pre-Treatment (Average Reliability Score of News)

Decile	Threshold	ITT Model	CACE Model (Weak Compliance Measure)	CACE Model (Strong Compliance Measure)	Sample Size
Bottom 10 Percent	78.255	3.707 (1.824)*	4.129 (1.891)*	4.415 (2.139)*	78
Bottom 20 Percent	85.347	1.87 (1.201)	2.045 (1.238)	2.15 (1.386)	157
Bottom 30 Percent	87.482	1.19 (0.799)	1.277 (0.811)	1.351 (0.904)	237
Bottom 40 Percent	88.383	1.004 (0.614)	1.083 (0.63)	1.106 (0.714)	315
Bottom 50 Percent	89.167	0.599 (0.516)	0.644 (0.527)	0.63 (0.593)	396
Bottom 60 Percent	90	0.558 (0.423)	0.602 (0.436)	0.653 (0.497)	493
Bottom 70 Percent	90.773	0.44 (0.382)	0.476 (0.396)	0.515 (0.453)	549
Bottom 80 Percent	92.462	0.444 (0.343)	0.477 (0.354)	0.518 (0.404)	629
Bottom 90 Percent	95	0.619 (0.336)	0.655 (0.349)	0.714 (0.4)	712

$^{***}p < 0.001$; $^{**}p < 0.01$; $^{*}p < 0.05$

Table S102: Testing the Effect of the Intervention on Proportion of News Diet That is Unreliable with Covariate-Adjusted Models (HC2 Robust standard errors) (After July 1st) on Different Samples of Respondents by Decile of their Quality of News Consumption Pre-Treatment (Average Reliability Score of News)

Decile	Threshold	ITT Model	CACE Model (Weak Compliance Measure)	CACE Model (Strong Compliance Measure)	Sample Size
Bottom 10 Percent	78.255	-0.039 (0.027)	-0.039 (0.029)	-0.034 (0.033)	70
Bottom 20 Percent	85.347	-0.025 (0.016)	-0.026 (0.017)	-0.023 (0.019)	141
Bottom 30 Percent	87.482	-0.013 (0.011)	-0.012 (0.011)	-0.011 (0.012)	215
Bottom 40 Percent	88.383	-0.008 (0.008)	-0.007 (0.008)	-0.006 (0.01)	289
Bottom 50 Percent	89.167	-0.007 (0.006)	-0.007 (0.007)	-0.006 (0.008)	369
Bottom 60 Percent	90	-0.008 (0.005)	-0.008 (0.006)	-0.006 (0.006)	459
Bottom 70 Percent	90.773	-0.007 (0.005)	-0.007 (0.005)	-0.005 (0.006)	511
Bottom 80 Percent	92.462	-0.006 (0.004)	-0.006 (0.005)	-0.005 (0.005)	585
Bottom 90 Percent	95	-0.005 (0.004)	-0.005 (0.004)	-0.003 (0.005)	663

$^{***}p < 0.001$; $^{**}p < 0.01$; $^{*}p < 0.05$

Table S103: Testing the Effect of the Intervention on Proportion of News Diet That is Reliable with Covariate-Adjusted Models (HC2 Robust standard errors) (After July 1st) on Different Samples of Respondents by Decile of their Quality of News Consumption Pre-Treatment (Average Reliability Score of News)

Decile	Threshold	ITT Model	CACE Model (Weak Compliance Measure)	CACE Model (Strong Compliance Measure)	Sample Size
Bottom 10 Percent	78.255	0.036 (0.034)	0.038 (0.036)	0.023 (0.04)	70
Bottom 20 Percent	85.347	0.023 (0.033)	0.025 (0.035)	0.021 (0.039)	141
Bottom 30 Percent	87.482	0.036 (0.026)	0.037 (0.027)	0.033 (0.03)	215
Bottom 40 Percent	88.383	0.039 (0.021)	0.041 (0.022)	0.036 (0.025)	289
Bottom 50 Percent	89.167	0.06 (0.019)**	0.063 (0.02)**	0.063 (0.022)**	369
Bottom 60 Percent	90	0.034 (0.018)	0.036 (0.019)	0.035 (0.021)	459
Bottom 70 Percent	90.773	0.024 (0.017)	0.026 (0.018)	0.023 (0.02)	511
Bottom 80 Percent	92.462	0.01 (0.016)	0.011 (0.017)	0.007 (0.019)	585
Bottom 90 Percent	95	0.003 (0.015)	0.005 (0.015)	0 (0.018)	663

$^{***}p < 0.001$; $^{**}p < 0.01$; $^{*}p < 0.05$

Table S104: Testing the Effect of the Intervention on Count of Unreliable News Consumed with Covariate-Adjusted Models (HC2 Robust standard errors) (After July 1st) on Different Samples of Respondents by Decile of their Quality of News Consumption Pre-Treatment (Average Reliability Score of News)

Decile	Threshold	ITT Model	CACE Model (Weak Compliance Measure)	CACE Model (Strong Compliance Measure)	Sample Size
Bottom 10 Percent	78.255	-0.163 (0.104)	-0.174 (0.112)	-0.189 (0.127)	74
Bottom 20 Percent	85.347	-0.042 (0.072)	-0.044 (0.077)	-0.045 (0.085)	147
Bottom 30 Percent	87.482	0.001 (0.05)	0.001 (0.053)	0.004 (0.059)	226
Bottom 40 Percent	88.383	0.014 (0.04)	0.016 (0.043)	0.021 (0.049)	302
Bottom 50 Percent	89.167	-0.005 (0.033)	-0.006 (0.035)	-0.004 (0.04)	383
Bottom 60 Percent	90	-0.004 (0.028)	-0.005 (0.03)	0.005 (0.034)	479
Bottom 70 Percent	90.773	-0.007 (0.027)	-0.008 (0.028)	-0.001 (0.032)	531
Bottom 80 Percent	92.462	-0.001 (0.024)	-0.001 (0.025)	0.006 (0.029)	608
Bottom 90 Percent	95	0.007 (0.022)	0.007 (0.023)	0.016 (0.026)	687

$^{***}p < 0.001$; $^{**}p < 0.01$; $^{*}p < 0.05$

Table S105: Testing the Effect of the Intervention on Count of Reliable News Consumed with Covariate-Adjusted Models (HC2 Robust standard errors) (After July 1st) on Different Samples of Respondents by Decile of their Quality of News Consumption Pre-Treatment (Average Reliability Score of News)

Decile	Threshold	ITT Model	CACE Model (Weak Compliance Measure)	CACE Model (Strong Compliance Measure)	Sample Size
Bottom 10 Percent	78.255	-0.034 (0.094)	-0.044 (0.101)	-0.078 (0.114)	74
Bottom 20 Percent	85.347	-0.045 (0.055)	-0.052 (0.059)	-0.07 (0.065)	147
Bottom 30 Percent	87.482	0.012 (0.05)	0.004 (0.053)	-0.019 (0.056)	225
Bottom 40 Percent	88.383	0.02 (0.043)	0.014 (0.046)	-0.004 (0.051)	300
Bottom 50 Percent	89.167	0.009 (0.039)	0.003 (0.041)	-0.014 (0.046)	379
Bottom 60 Percent	90	0.004 (0.037)	0.002 (0.039)	-0.01 (0.042)	472
Bottom 70 Percent	90.773	0.01 (0.035)	0.007 (0.037)	-0.002 (0.04)	524
Bottom 80 Percent	92.462	0.007 (0.033)	0.004 (0.035)	-0.004 (0.038)	599
Bottom 90 Percent	95	-0.014 (0.031)	-0.016 (0.032)	-0.026 (0.036)	678

$^{***}p < 0.001$; $^{**}p < 0.01$; $^{*}p < 0.05$

Table S106: Testing the Effect of the Intervention on Reliability Score of News Diet with Covariate-Adjusted Models (HC2 Robust standard errors) (After July 1st) on Different Samples of Respondents by Decile of their Quality of News Consumption Pre-Treatment (Average Reliability Score of News)

Decile	Threshold	ITT Model	CACE Model (Weak Compliance Measure)	CACE Model (Strong Compliance Measure)	Sample Size
Bottom 10 Percent	78.255	5.863 (1.858)**	5.988 (1.892)**	5.813 (2.066)**	70
Bottom 20 Percent	85.347	3.187 (1.255)*	3.215 (1.284)*	3.22 (1.397)*	141
Bottom 30 Percent	87.482	1.694 (0.855)*	1.655 (0.863)	1.665 (0.948)	215
Bottom 40 Percent	88.383	1.277 (0.677)	1.261 (0.694)	1.281 (0.782)	289
Bottom 50 Percent	89.167	1.194 (0.555)*	1.192 (0.567)*	1.221 (0.63)	369
Bottom 60 Percent	90	0.933 (0.475)	0.941 (0.493)	1.006 (0.55)	459
Bottom 70 Percent	90.773	0.849 (0.435)	0.856 (0.452)	0.921 (0.507)	511
Bottom 80 Percent	92.462	0.711 (0.4)	0.712 (0.414)	0.757 (0.464)	585
Bottom 90 Percent	95	0.648 (0.376)	0.631 (0.391)	0.662 (0.44)	663

$^{***}p < 0.001$; $^{**}p < 0.01$; $^{*}p < 0.05$

SJ Distribution of Pre-Treatment Behavioral Measures

Figure S5: Distribution of Average NewsGuard Reliability Score of Online News Diet (Pre-Treatment)

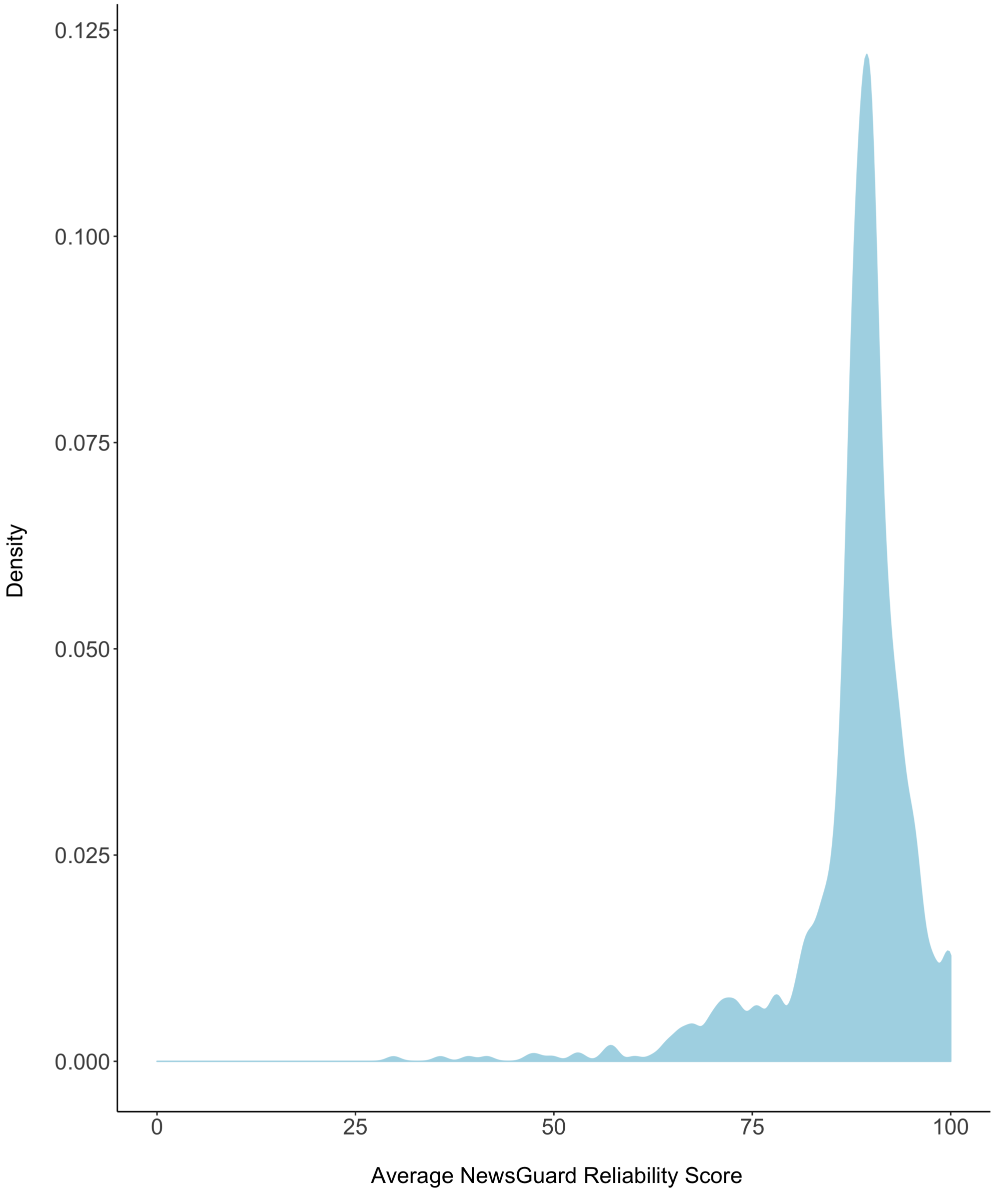

Figure S6: Distribution of Proportion of Unreliable News in Online News Diet (Pre-Treatment)

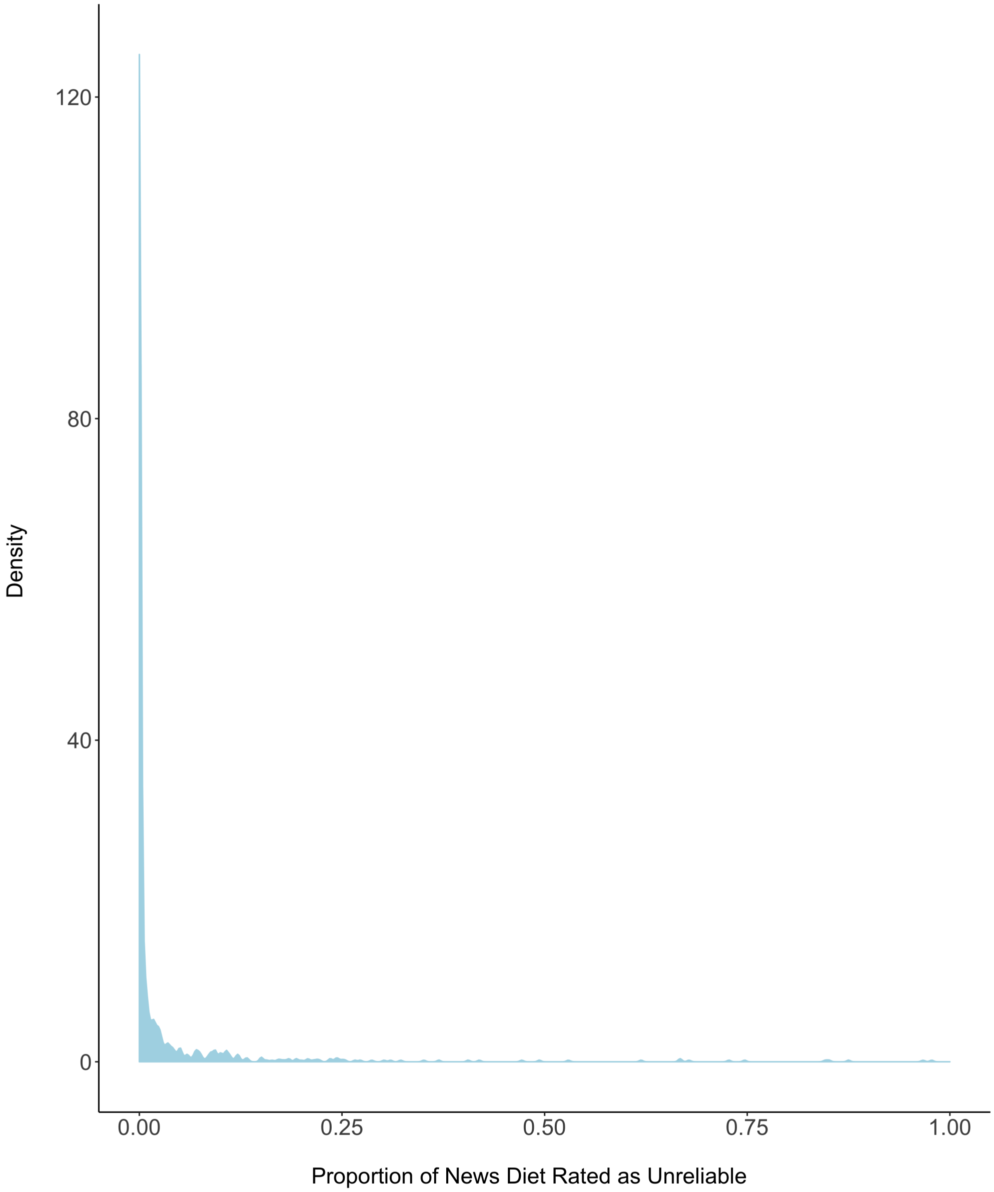

Figure S7: Distribution of Count of Visits to Online News Sites Rated by NewsGuard (Max=2,000)(Pre-Treatment)

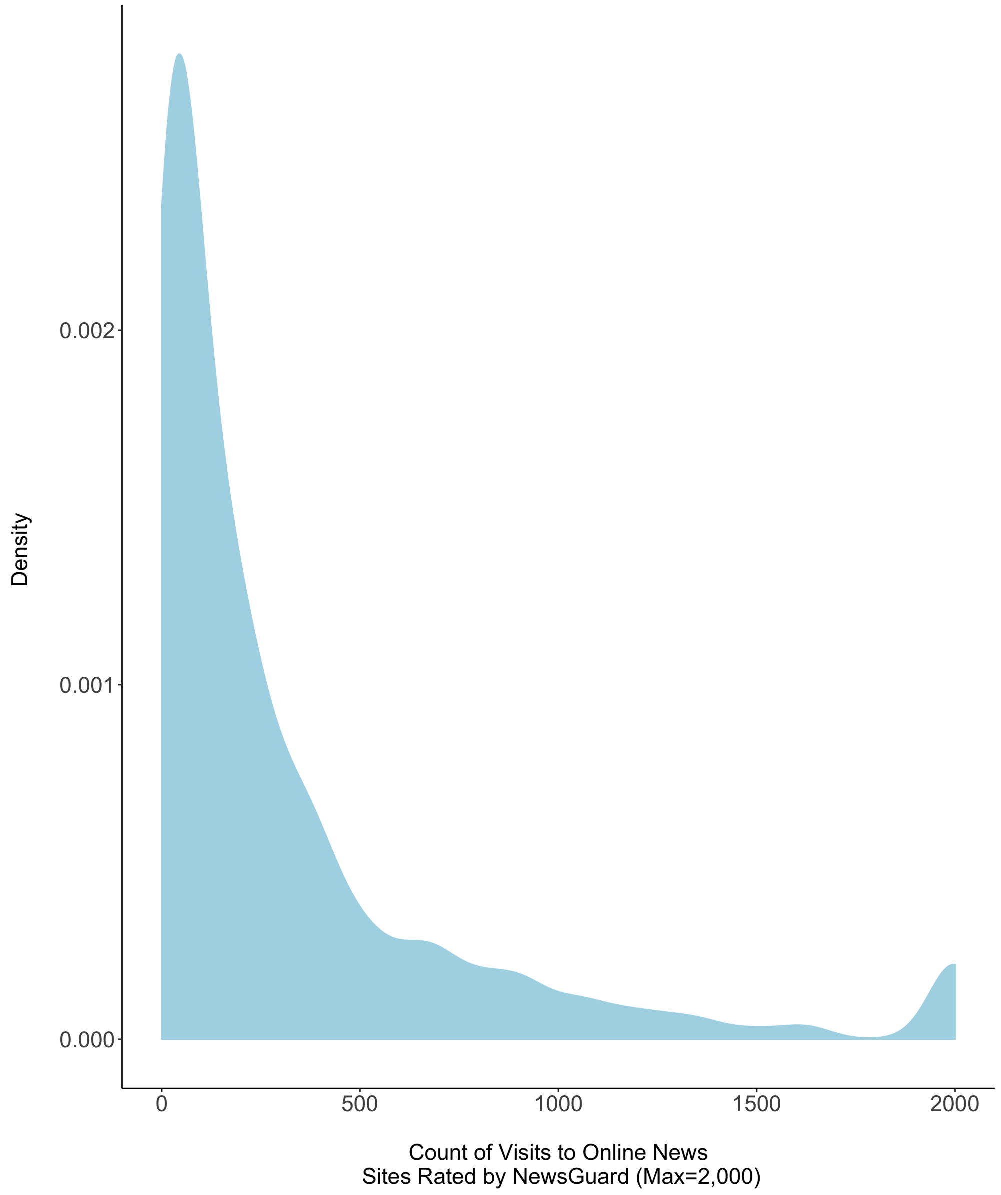

Figure S8: Distribution of Count of Google Search Referrals to an Online News Sites Rated by NewsGuard (Pre-Treatment)

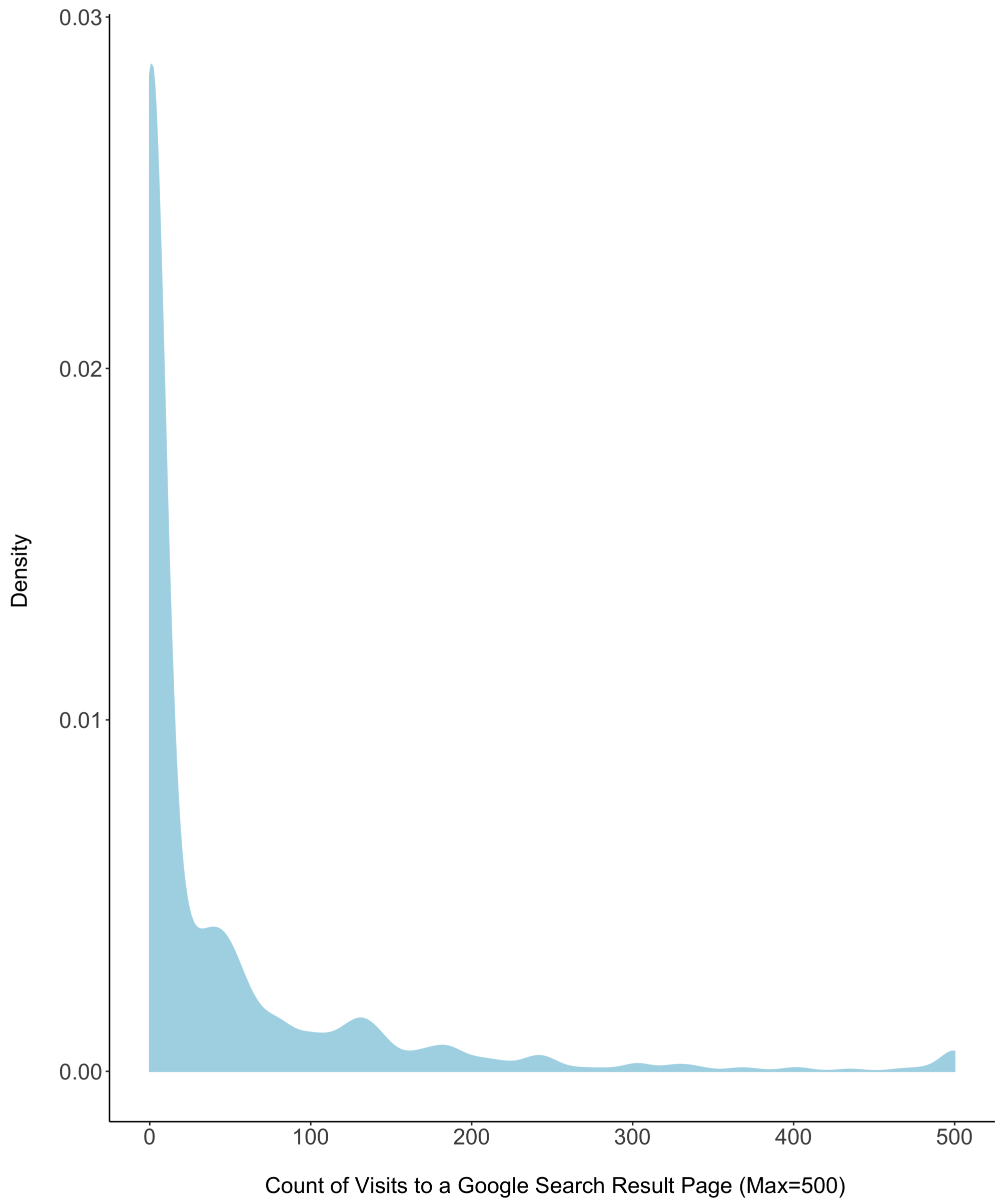

Figure S9: Distribution of Time Spent on Facebook (Pre-Treatment)

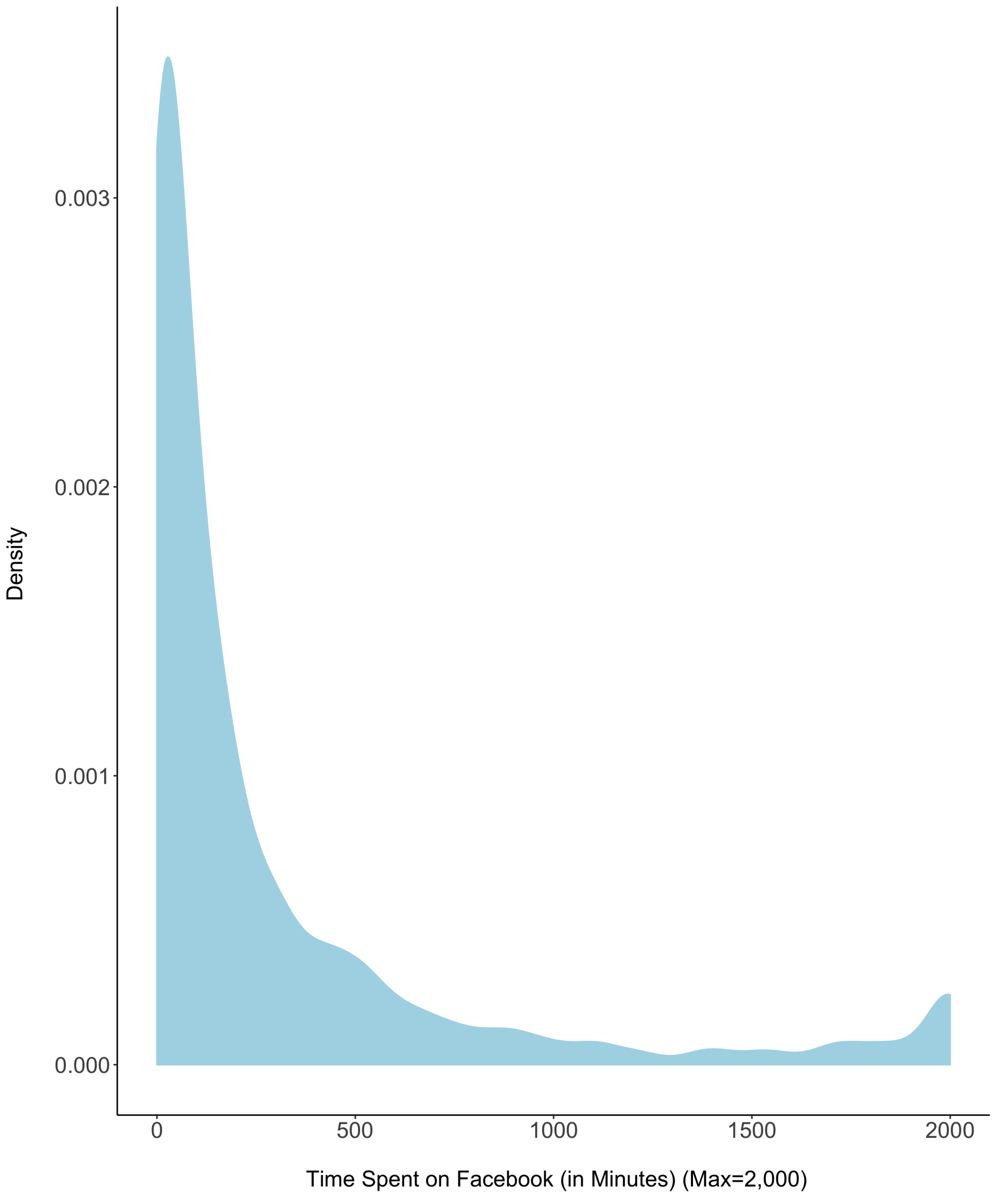

Figure S10: Distribution of Time Spent on Twitter (Pre-Treatment)

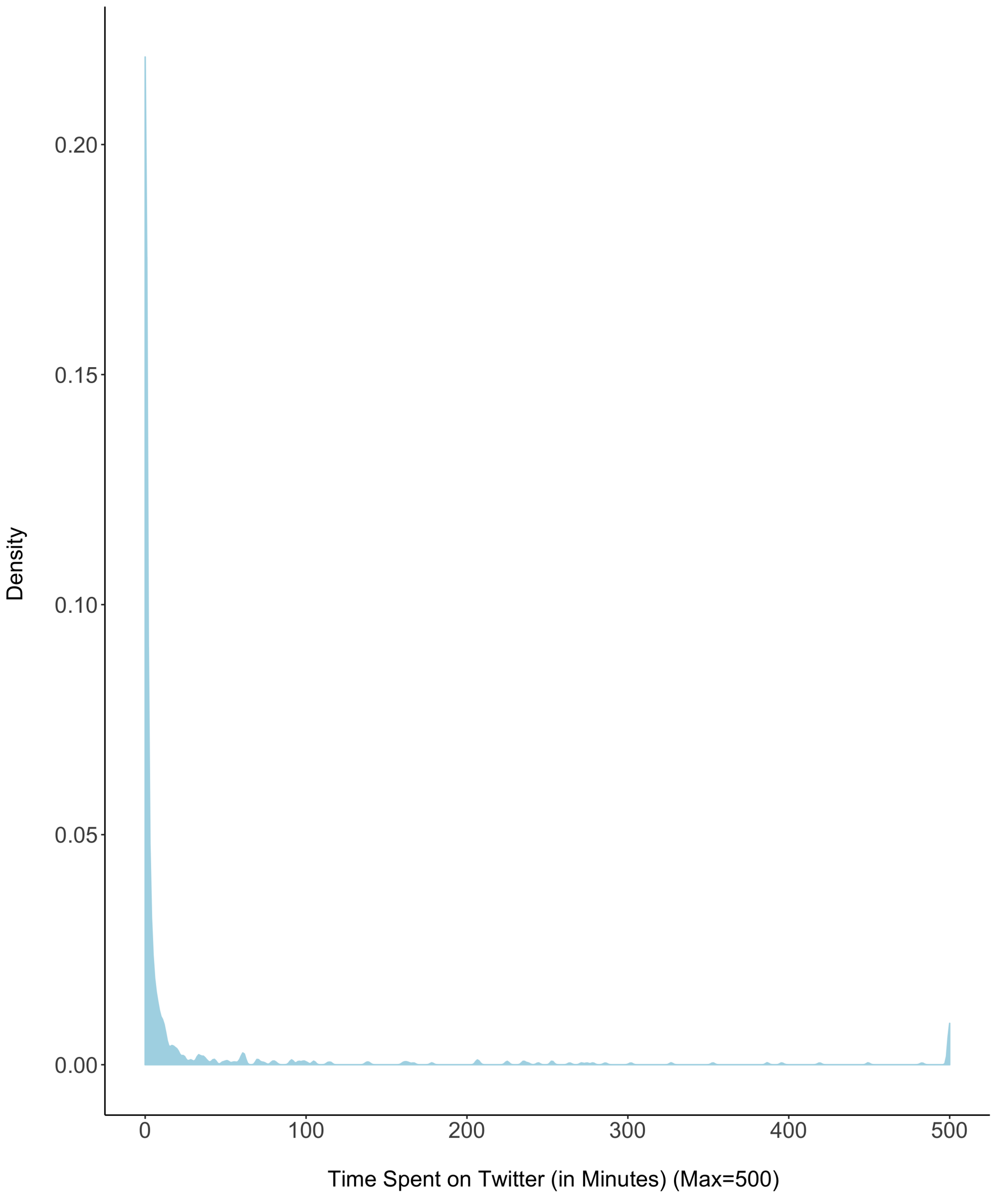

SK Figure: Effect of Intervention on Attitudinal Measures

Figure S11: This figure presents estimates of the effect of the intervention (with 95% confidence intervals) on our pre-registered attitudinal measures. The effect is reported in standard deviations of that measure (pre-treatment).

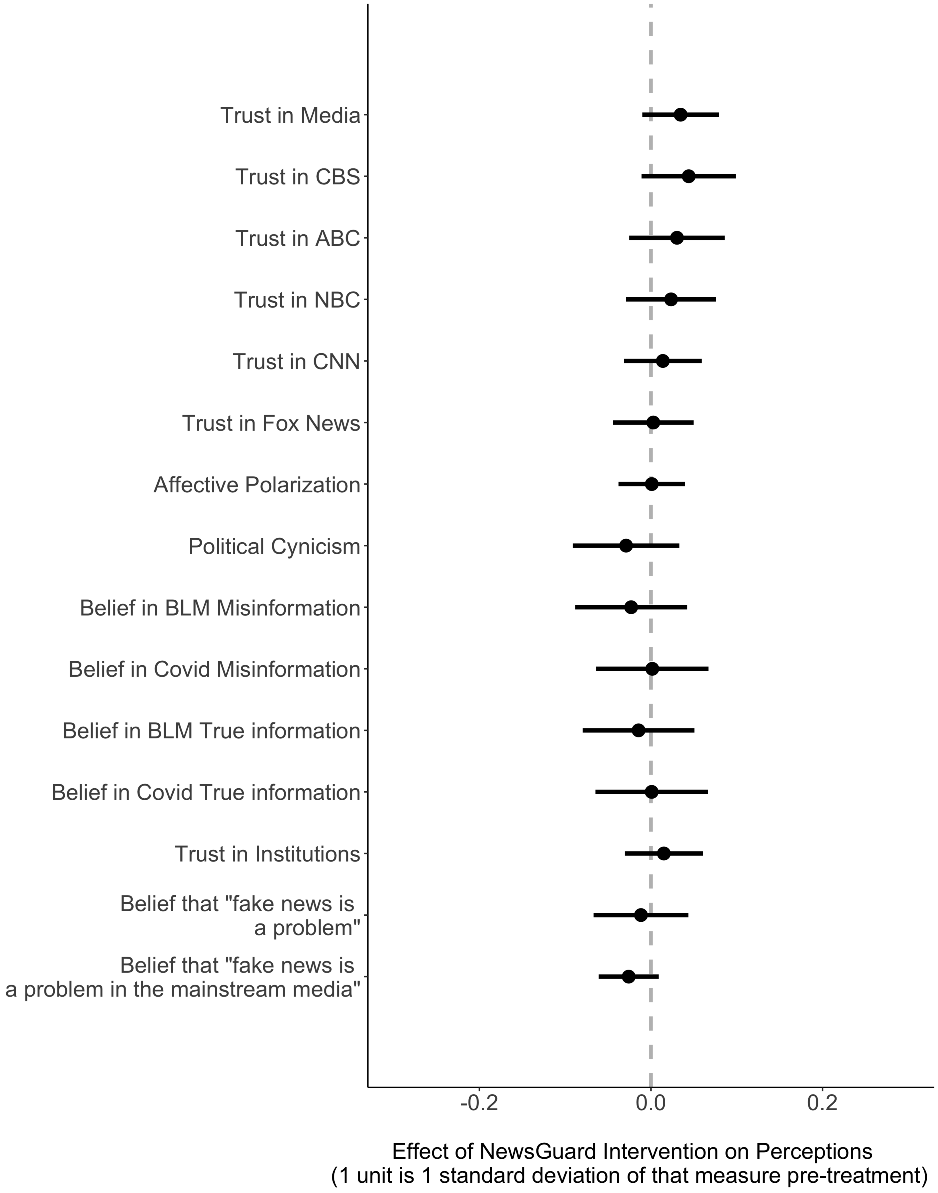

SL Figure: Proportion of the average daily proportion of unreliable news viewed by the treatment group

Figure S12: This figure presents the average daily proportion of unreliable news viewed by the treatment group across this study (with 95% confidence intervals)

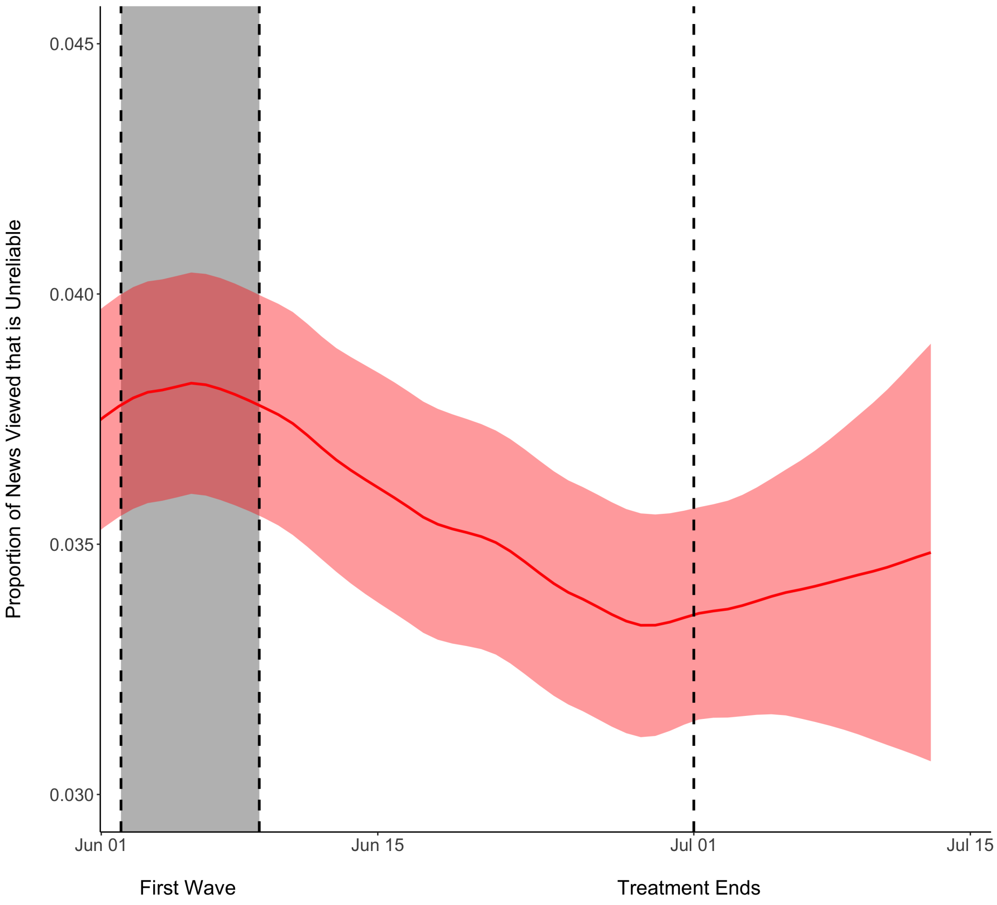

SM Balance Table and Density Plots

Table S107: Average Statistics for Behavioral Measures of Online News Quality by Control and Treatment Group in Each Time Period of Interest

	Control	Treatment	Difference (Treatment - Control)
Number of respondents	356	612	NA
Average Proportion of News Viewed that was "Unreliable" (Pre-Treatment period)	0.03143	0.03185	0.00042
Average Proportion of News Viewed that was "Unreliable" (Treatment period)	0.03054	0.03107	0.00053
Average Proportion of News Viewed that was "Unreliable" (Post-Treatment period)	0.03215	0.03057	-0.00159
Average Proportion of News Viewed that was "Reliable" (Pre-Treatment period)	0.67395	0.67513	0.00118
Average Proportion of News Viewed that was "Reliable" (Treatment period)	0.68441	0.67339	-0.01103
Average Proportion of News Viewed that was "Reliable" (Post-Treatment period)	0.70309	0.71415	0.01106
Average Count (log) of News Viewed that was "Unreliable" (Pre-Treatment period)	0.32938	0.31552	-0.01386
Average Count (log) of News Viewed that was "Unreliable" (Treatment period)	0.34902	0.37805	0.02902
Average Count (log) of News Viewed that was "Unreliable" (Post-Treatment period)	0.24346	0.25249	0.00904
Average Count (log) of News Viewed that was "Reliable" (Pre-Treatment period)	1.73446	1.73218	-0.00228
Average Count (log) of News Viewed that was "Reliable" (Treatment period)	1.85586	1.83976	-0.0161
Average Count (log) of News Viewed that was "Reliable" (Post-Treatment period)	1.56868	1.5284	-0.04028
Average Reliability Score of News Viewed (Pre-Treatment period)	87.7879	87.52881	-0.25909
Average Reliability Score of News Viewed (Treatment period)	87.26024	87.31514	0.0549
Average Reliability Score of News Viewed (Post-Treatment period)	87.11857	87.29597	0.17741

Figure S13: This figure presents the distribution of the proportion of news sites viewed that are unreliable among respondents in the treatment and control groups with a vertical dashed line indicating the mean reliability score for each group. Panels A, B, and C present these distributions during the pre-treatment period, treatment period, and post-treatment period respectively.

(a) Pre-Treatment Period

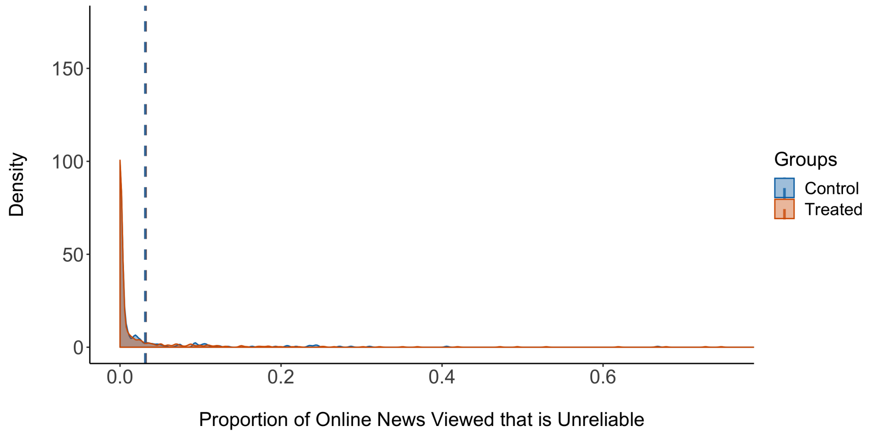

(b) Treatment Period

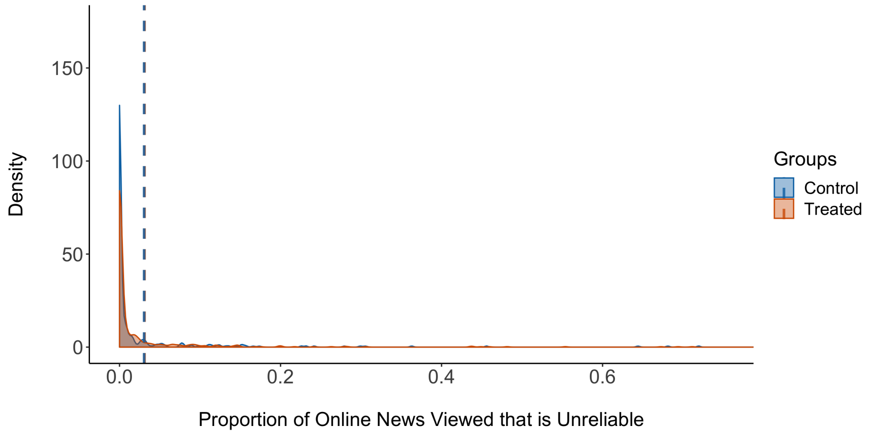

(c) Post-Treatment Period

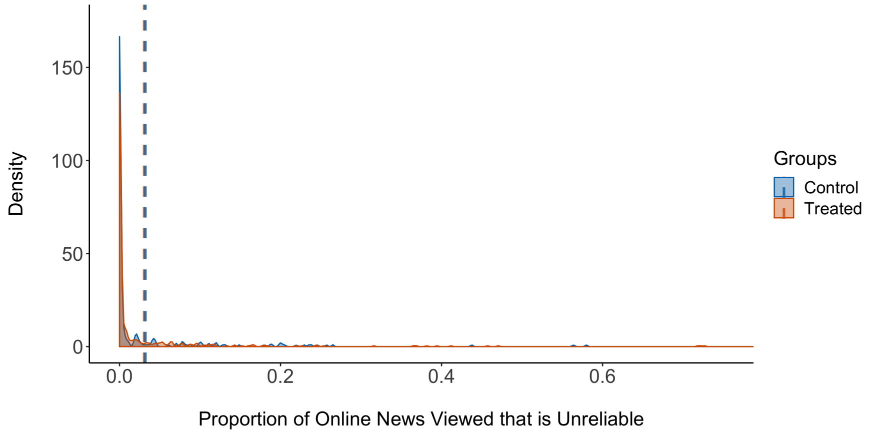

Figure S14: This figure presents the distribution of the proportion of news sites viewed that are reliable among respondents in the treatment and control groups with a vertical dashed line indicating the mean reliability score for each group. Panels A, B, and C present these distributions during the pre-treatment period, treatment period, and post-treatment period respectively.

(a) Pre-Treatment Period

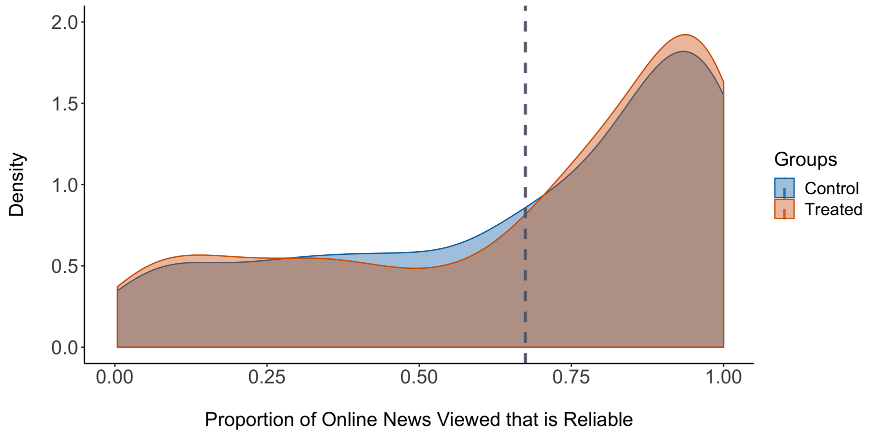

(b) Treatment Period

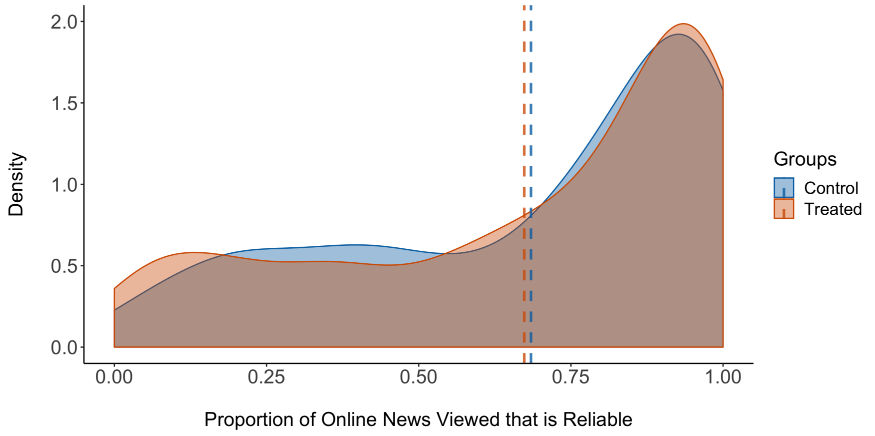

(c) Post-Treatment Period

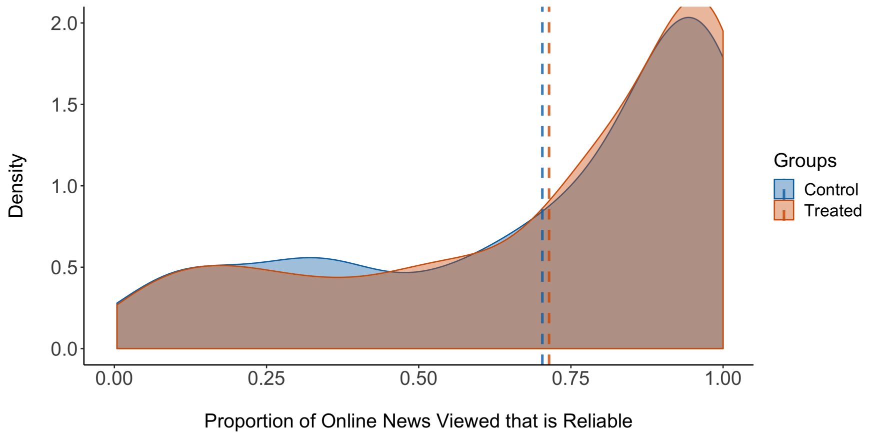

Figure S15: This figure presents the distribution of the count of unreliable news sites viewed (log) among respondents in the treatment and control groups with a vertical dashed line indicating the mean reliability score for each group. Panels A, B, and C present these distributions during the pre-treatment period, treatment period, and post-treatment period respectively.

(a) Pre-Treatment Period

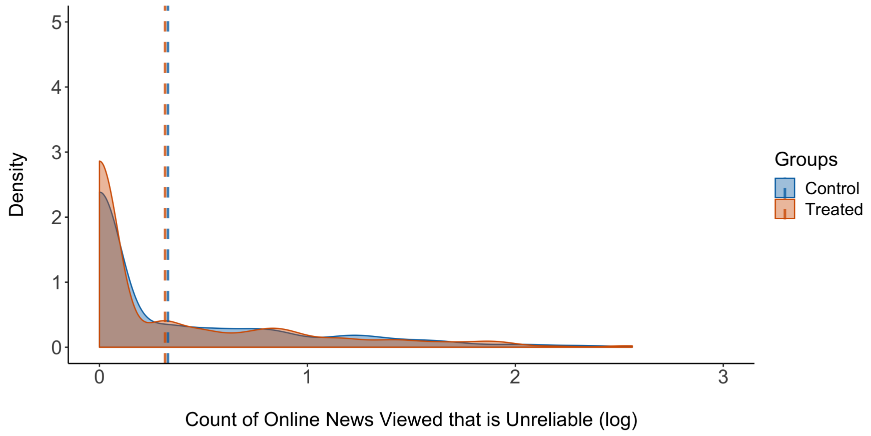

(b) Treatment Period

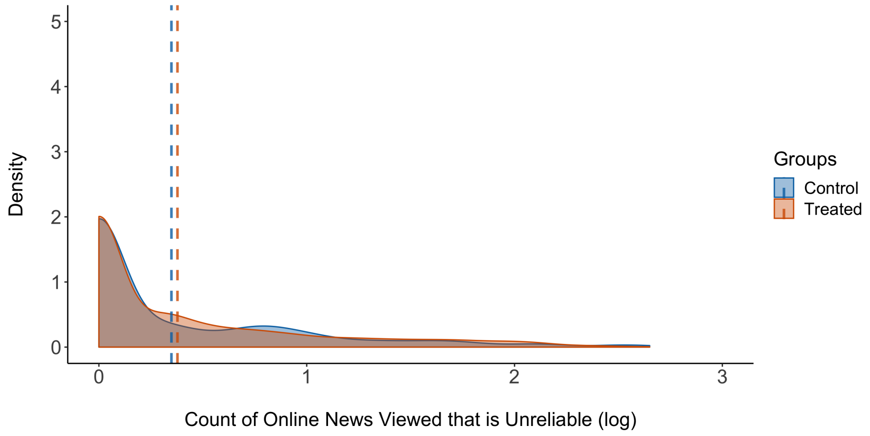

(c) Post-Treatment Period

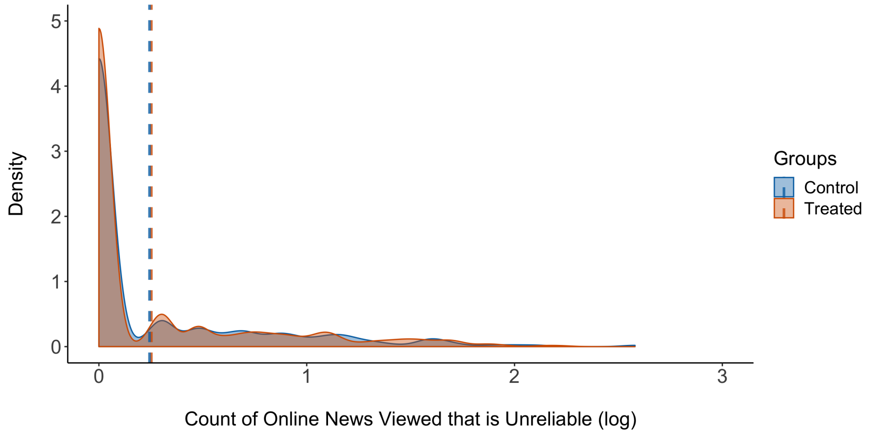

Figure S16: This figure presents the distribution of the count of reliable news sites viewed (log) among respondents in the treatment and control groups with a vertical dashed line indicating the mean reliability score for each group. Panels A, B, and C present these distributions during the pre-treatment period, treatment period, and post-treatment period respectively.

(a) Pre-Treatment Period

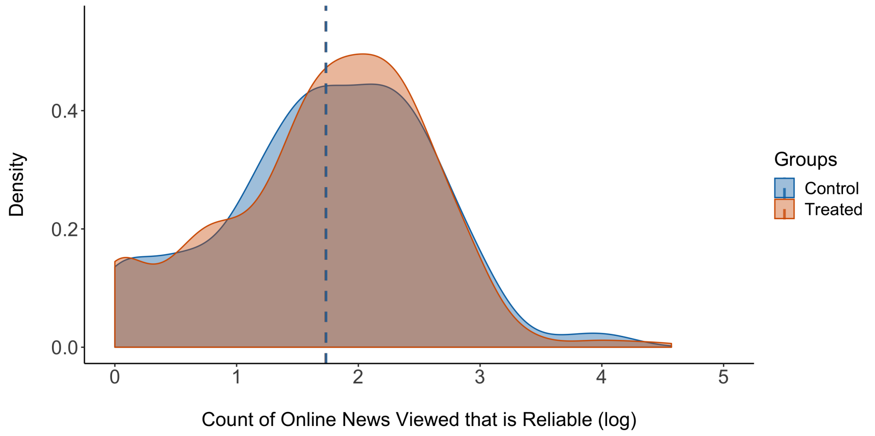

(b) Treatment Period

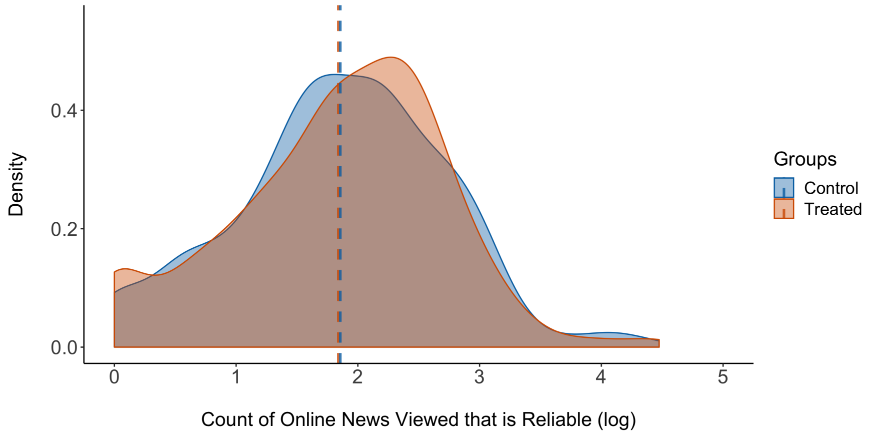

(c) Post-Treatment Period

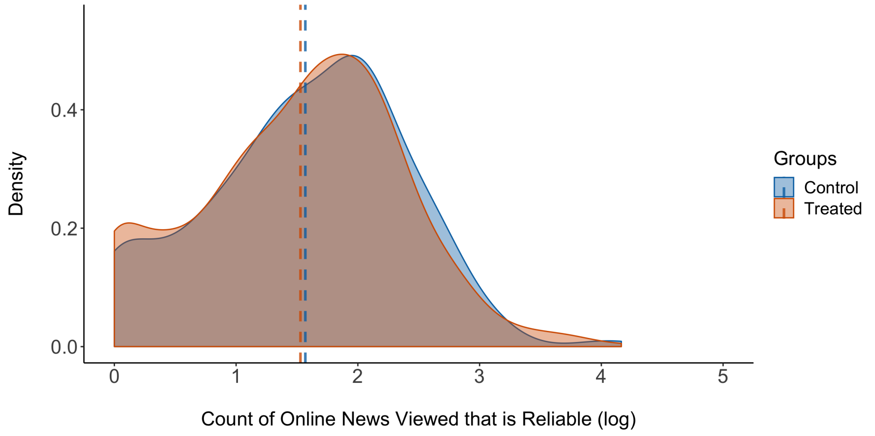

SN Results From Covariate-Adjusted Models using Mobile Behavioral Data

Figure S17: This figure presents estimates of the effect of the intervention (ITT, with 95% confidence intervals) on our mobile behavioral measures in the two periods after treatment assignment: before July 1 when the NewsGuard extension was freely available and the two-week period between July 1–13 when the NewsGuard extension was disabled. The effect is reported in standard deviations of that measure (pre-treatment).

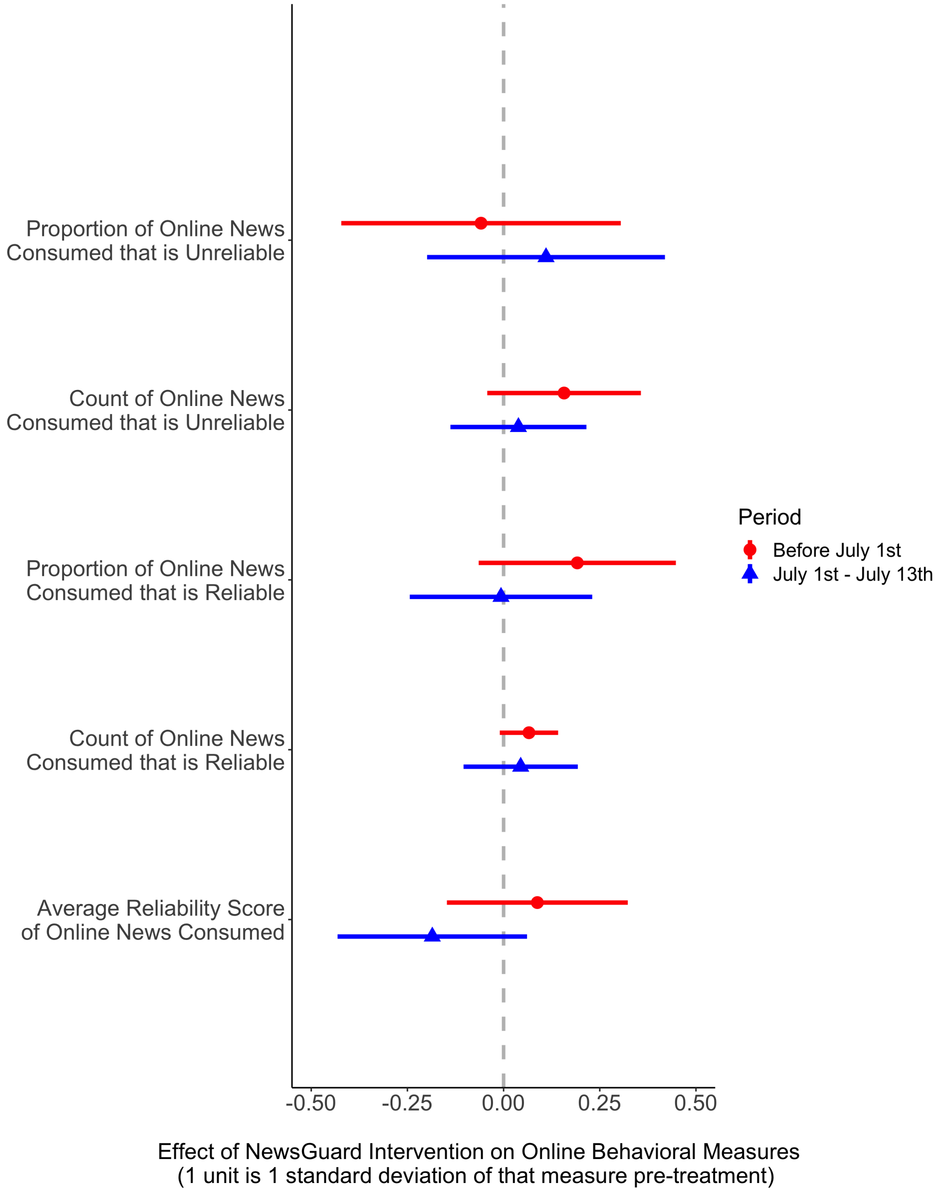

Table S108: This table presents the number and percentage of the Pulse sample for which we collected only web tracking data from their desktop/laptop, only from their mobile device, or from both

	Desktop/Laptop Only	Mobile Only	Both Desktop/Laptop and Mobile
Percentage of Pulse Sample	79.14	18.24	2.62
Number of Respondents	937	216	31

REFERENCES AND NOTES

1. D. M. J. Lazer, M. A. Baum, Y. Benkler, A. J. Berinsky, K. M. Greenhill, F. Menczer, M. J. Metzger, B. Nyhan, G. Pennycook, D. Rothschild, M. Schudson, S. A. Sloman, C. R. Sunstein, E. A. Thorson, D. J. Watts, J. L. Zittrain, The science of fake news. *Science*, **359**, 1094–1096 (2018).

2. S. Vosoughi, D. Roy, S. Aral, The spread of true and false news online. *Science*, **359**, 1146–1151 (2018).

3. N. Grinberg, J. Kenneth, L. Friedland, B. Swire-Thompson, D. Lazer, Fake news on Twitter during the 2016 U.S. presidential election. *Science*, **363**, 374–378 (2019).

4. A. Guess, J. Nagler, J. Tucker, Less than you think: Prevalence and predictors of fake news dissemination on Facebook. *Sci. Adv.*, **5**, eaau4586 (2019).

5. M. Osmundsen, A. Bor, P. B. Vahlstrup, A. Bechmann, M. B. Petersen, Partisan polarization is the primary psychological motivation behind political fake news sharing on Twitter. *Am. Polit. Sci. Rev.*, **115**, 999–1015 (2021).

6. D. J. Flynn, B. Nyhan, J. Reifler, The nature and origins of misperceptions: Understanding false and unsupported beliefs about politics. *Polit. Psychol.*, **38**, 127–150 (2017).

7. N. Anspach, N. M. Jennings, T. Jay, K. Arceneaux, A little bit of knowledge: Facebook's News Feed and self-perceptions of knowledge. *Research & Politics*, **6**, 127–150 (2019).

8. A. Guess, P. Barber, S. Munzert, J. Yang, The consequences of online partisan media. *Proc. Natl. Acad. Sci. U.S.A.* **118**, e2013464118 (2021).

9. B. J. Scott, S. Felix, P. N. Howard, R. K. Nielsen, "Sample matching: Representative sampling from internet panels" (Reuters Institute Report, 2020).

10. U. K. H. Ecker, S. Lewandowsky, D. T. W. Tang, Explicit warnings reduce but do not eliminate the continued influence of misinformation. *Mem. Cognit.*, **38**, 1087–1100 (2010).

11. K. Clayton, S. Blair, J. A. Busam, S. Forstner, J. Glance, G. Green, A. Kawata, A. Kovvuri, J. Martin, E. Morgan, M. Sandhu, R. Sang, R. Scholz-Bright, A. T. Welch, A. G. Wolff, A. Zhou, B. Nyhan, Real solutions for fake news? Measuring the effectiveness of general warnings and fact-check tags in reducing belief in false stories on social media. *Polit. Behav.*, **42**, 1073–1095 (2020).

12. A. M. Guess, M. Lerner, B. Lyons, J. M. Montgomery, B. Nyhan, J. Reifler, N. Sircar, A digital media literacy intervention increases discernment between mainstream and false news in the United States and India. *Proc. Natl. Acad. Sci. U.S.A.*, **117**, 15536–15545 (2020).

13. S. Badrinathan, Educative interventions to combat misinformation: Evidence from a field experiment in India. *Am. Polit. Sci. Rev.* **115**, 1325–1341 (2021).

14. G. Pennycook, A. Bear, E. T. Collins, D. G. Rand, The implied truth effect: Attaching warnings to a subset of fake news headlines increases perceived accuracy of headlines without warnings. *Manage. Sci.*, **66**, 4944–4957 (2020).

15. B. Nyhan, J. Reifler, P. A. Ubel, The hazards of correcting myths about health care reform. *Med. Care*, **51**, 127–132 (2013).

16. P. Lorenz-Spreen, S. Lewandowsky, R. C. Sunstein, R. Hertwig, How behavioural sciences can promote truth, autonomy and democratic discourse online. *Nat. Hum. Behav.* **4**, 1102–1109 (2020).

17. G. Gigerenzer, R. Selten, *Bounded Rationality: The Adaptive Toolbox* (MIT Press, 2002).

18. G. Pennycook, D. G. Rand, Fighting misinformation on social media using crowdsourced judgments of news source quality. *Proc. Natl. Acad. Sci. U.S.A.* **116**, 2521–2526 (2019).

19. S. Messing, S. J. Westwood, Selective exposure in the age of social media: Endorsements trump partisan source affiliation when selecting news online. *Communic. Res.*, **41**, 1042–1063 (2014).

20. S. Munzert, P. BarberÁ, A. Guess, J. H. Yang, Do online voter guides empower citizens? Evidence from a field experiment with digital trace data. *Public Opin. Q.*, **84**, 675–698 (2021).

21. S. Munzert, P. Selb, A. Gohdes, L. F. Stoetzer, W. Lowe, Tracking and promoting the usage of a COVID-19 contact tracing app. *Nat. Hum. Behav.*, **5**, 247–255 (2021).

22. A. Kim, P. L. Moravec, A. R. Dennis, Combating fake news on social media with source ratings: The effects of user and expert reputation ratings. *J. Manag. Inf. Syst.* **36**, 931–968 (2019).

23. A. Kim, A. R. Dennis, Says who? The effects of presentation format and source rating on fake news in social media. *MIS Q.* **43**, 1025–1039 (2019).

24. Gallup and Knight Foundation, "Assessing the effect of news source ratings on news content" (2018); https://knightfoundation.org/reports/assessing-the-effect-of-news-source-ratings-on-news-content/.

25. S. Iyengar, K. S. Hahn, Red media, blue media: Evidence of ideological selectivity in media use. *J. Commun.*, **59**, 19–39 (2009).

26. K. Clayton, J. Davis, K. Hinckley, Y. Horiuchi, Partisan motivated reasoning and misinformation in the media: Is news from ideologically uncongenial sources more suspicious? *Jpn. J. Political Sci.* **20**, 129–142 (2019).

27. N. Dias, G. Pennycook, D. G. Rand, Emphasizing publishers does not effectively reduce susceptibility to misinformation on social media. *Harv. Kennedy Sch. Misinformation Rev.* **1** 10.37016/mr-2020-001 (2020).

28. NewsGuard's Online Source Rating Tool: User Experience (Gallup, 2019).

29. C. I. Hovland, I. L. Janis, H. H. Kelley, *Communication and Persuasion* (Yale Univ. Press, 1953).

30. A. J. Flanagin, M. J. Metzger, Perceptions of internet information credibility. *Journal Mass Commun. Q.* **77**, 515–540 (2000).

31. N. Walter, S. T. Murphy, How to unring the bell: A meta-analytic approach to correction of misinformation. *Commun. Monogr.* **85**, 423–441 (2018).

32. A. M. Guess, D. Lockett, B. Lyons, J. M. Montgomery, B. Nyhan, J. Reifler, "Fake news" may have limited effects beyond increasing beliefs in false claims. *Harv. Kennedy Sch. Misinformation Rev.* **1** 10.37016/mr-2020-004 (2020).

33. S. Loomba, A. de Figueiredo, S. J. Piatek, K. de Graaf, H. J. Larson, Measuring the impact of COVID-19 vaccine misinformation on vaccination intent in the UK and USA. *Nat. Hum. Behav.*, **5**, 337–348 (2021).

34. H. Wasserman, D. Madrid-Morales, An exploratory study of "fake news" and media trust in Kenya, Nigeria and South Africa. *Afr. Journal. Stud.* **40** 107–123 (2019).

35. K. Ognyanova, D. Lazer, R. E. Robertson, C. Wilson, Misinformation in action: Fake news exposure is linked to lower trust in media, higher trust in government when your side is in power. *Harv. Kennedy Sch. Misinformation Rev.* **1** 10.37016/mr-2020-024 (2020).

36. R. R. Lau, D. J. Andersen, T. M. Ditonto, M. S. Kleinberg, D. P. Redlawsk, Effect of media environment diversity and advertising tone on information search, selective exposure, and affective polarization. *Polit. Behav.*, **39** 231–255 (2017).

37. E. Suhay, E. Bello-Pardo, B. Maurer, The polarizing effects of online partisan criticism: Evidence from two experiments. *Int. J. Press Polit.*, **23**, 95–115 (2018).

38. Y. Tsfati, L. Nir, Frames and reasoning: Two pathways from selective exposure to affective polarization. *Int. J. Commun.*, **11**, 301–322 (2017).

39. A. M. Guess, B. Nyhan, J. Reifler, Exposure to untrustworthy websites in the 2016 US election. *Nat. Hum. Behav.*, **4**, 472–480 (2020).

40. A. S. Gerber, J. G. Gimpel, D. P. Green, D. R. Shaw, How large and long-lasting are the persuasive effects of televised campaign ads? Results from a randomized field experiment. *Am. Polit. Sci. Rev.* **105**, 135–150 (2011).

41. A. Coppock, E. Ekins, D. Kirby, The long-lasting effects of newspaper op-eds on public opinion. *Q. J. Polit. Sci.*, **13**, 59–87 (2018).

42. B. Rainey, Arguing for a negligible effect. *Am. J. Pol. Sci.*, **58**, 1083–1091 (2014).

43. D. Lakens, Equivalence tests: A practical primer for *t* tests, correlations, and meta-analyses. *Soc. Psychol. Personal. Sci.*, **8**, 355–362 (2017).

44. E. Hartman, D. F. Hidalgo, An equivalence approach to balance and placebo tests. *Am. J. Pol. Sci.*, **62**, 1000–1013 (2018).

45. J. Cohen, *Statistical Power Analysis for the Behavioral Sciences* (Lawrence Erlbaum Associates, 1969).

46. G. W. Imbens, D. B. Rubin, *Causal Inference in Statistics, Social, and Biomedical Sciences* (Cambridge Univ. Press, 2015).

47. Z. Guan, E. Cutrell, An eye tracking study of the effect of target rank on web search, in *Proceedings of the SIGCHI Conference on Human Factors in Computing Systems*, San Jose, CA, 28 April to 3 May 2007 (Association for Computing Machinery, 2007), pp. 417–420.

48. S. Chaiken, The heuristic model of persuasion, in *Social Influence: The Ontario Symposium* (Lawrence Erlbaum Associates Inc., 1987), vol. 5, pp. 3–39.

49. M. Jurkowitz, A. Mitchell, E. Shearer, M. Walker, "U.S. media polarization and the 2020 election: A nation divided" (Pew Research Center, 2020); www.pewresearch.org/journalism/2020/01/24/u-s-media-polarization-and-the-2020-election-a-nation-divided/.

50. G. Pennycook, Z. Epstein, M. Mosleh, A. Arechar, D. Eckles, D. G. Rand, Shifting attention to accuracy can reduce misinformation online. *Nature*, **592**, 590–595 (2021).

51. S. Altay, A. Hacquin, H. Mercier, Why do so few people share fake news? It hurts their reputation. *New Media Soc.* 10.1177/1461444820969893 (2019).

52. J. Allen, B. Howland, M. Mobius, D. Rothschild, D. J. Watts, Evaluating the fake news problem at the scale of the information ecosystem. *Sci. Adv.* **6**, eaay3539 (2020).

53. H. Allcott, M. Gentzkow, C. Yu, Trends in the diffusion of misinformation on social media. *Res. Polit.* **6** 10.1177/2053168019848554 (2019).

54. A. M. Guess, (Almost) everything in moderation: New evidence on Americans' online media diets. *Am. J. Pol. Sci.*, **65**, 1007–1022, (2021).

55. J. Hainmueller, J. Mummolo, Y. Xu, How much should we trust estimates from multiplicative interaction models? Simple tools to improve empirical practice. *Polit. Anal.*, **27**, 163–192 (2019).

56. American Trends Panel (Pew, 2017).

57. A. M. Guess, B. Nyhan, J. Reifler, *American Trends Panel* (Poynter Media Trust Survey, 2019).

58. L. Dancey, The consequences of political cynicism: How cynicism shapes citizens' reactions to political scandals. *Polit. Behav.*, **34**, 411–423 (2012).

59. J. M. Miller, K. L. Saunders, C. E. Farhart, Conspiracy endorsement as motivated reasoning: The moderating roles of political knowledge and trust. *Am. J. Pol. Sci.*, **60**, 824–844 (2016).
